# An Advanced, Risk-Driven Sexual Health Curriculum for First-Year Internal Medicine Residents

**DOI:** 10.15766/mep_2374-8265.11287

**Published:** 2022-12-09

**Authors:** Maria Elizabeth Maldonado, Jennifer Rusiecki

**Affiliations:** 1 Assistant Professor, Department of Medicine, Baylor College of Medicine; 2 Assistant Professor, Department of Medicine, University of Chicago Medicine

**Keywords:** Safe Sex Education, Sexually Transmitted Illnesses, STIs, Ambulatory Care, History Taking, Human Sexuality, Infectious Disease, Primary Care, Case-Based Learning

## Abstract

**Introduction:**

Despite recommendations for annual chlamydia screening for young, sexually active females, chlamydia rates continue to increase nationally. Medical school students often receive limited training in obtaining comprehensive, risk-based sexual histories, leading to less screening for sexually transmitted illnesses (STIs). Consequently, many internal medicine (IM) residents feel unprepared for advanced sexual history taking and site-specific STI testing based on sexual practices.

**Methods:**

We developed a case-based interactive didactic session for IM and med/peds residents. We focused on more advanced topics, such as comprehensive sexual history taking, secondary site testing for STIs, and patient counseling.

**Results:**

Based on pre- and postcurriculum surveys, interns reported increases in comfort (scale: 1 = *very low comfort,* 5 = *very high comfort*) with counseling after positive STI results (*M*s: pre = 2.9, post = 3.5, *p* < .01) and providing education on safe sex practices (*M*s: pre = 3.0, post = 3.6, *p* < .01) and modest improvement in comfort with expedited partner therapy (EPT; *M*s: pre = 2.1, post = 2.5, *p* = .06). An increase in self-reported confidence with collecting site-specific testing (composite *M*s: pre = 2.7, post = 3.5, *p* < .01) was also seen. Percentage correct on knowledge questions increased from 48% to 78% postcurriculum (*p* = .01).

**Discussion:**

This curriculum demonstrated improvement in knowledge and comfort with sexual history taking, STI screening, and counseling. Comfort also improved with EPT counseling, but not significantly, which could be addressed in future iterations of the curriculum.

## Educational Objectives

By the end of this activity, learners will be able to:

1. Describe the steps to obtain a comprehensive sexual history and tailor this based on a patient's level of risk.
2. Perform complete, appropriate screenings for sexually transmitted illnesses based on sexual practices risk groups and patient characteristics.
3. Counsel patients on positive sexually transmitted illness results and safe sex practices and refer patients appropriately for pre-exposure prophylaxis for HIV.
4. Treat sexually transmitted illness and offer expedited partner therapy when appropriate.

## Introduction

Sexually transmitted illnesses (STIs) such as gonorrhea, syphilis, and chlamydia have been experiencing a resurgence. In 2020, 2.4 million cases of STIs were reported in the United States, with 1.6 million new cases of *Chlamydia trachomatis* and 677,000 new cases of gonorrhea reported nationally.^1^ The prevalence and morbidity of STIs are higher among low-income patients as well as among Black, American Indian/Alaska Native, Native Hawaiian/Other Pacific Islander, and Hispanic females.^2,3^

Of note, we use the terms *female* and *male* throughout this discussion in reference to biologic sex, inclusive of all gender identities including cisgender women, transgender men, and nonbinary individuals. In general, screening of transgender and gender diverse persons should be adapted based on anatomy and sexual practices.^4^

Sexual histories during primary care visits provide important details to help guide testing, diagnosis, and treatment of STIs as well as an opportunity to offer prevention-focused behavioral counseling in accordance with the United State Preventive Services Task Force (USPSTF) guidelines.^5,6^ A survey of 3,300 adults found that only one-quarter of patients reported being asked about sexual health or STIs by their primary care physicians during their last visit.^7^ One unique issue in primary care STI screening is that patients with varying levels of STI risk are seen. Providers must walk the line between performing a comprehensive history, time management, and patient expectations. They must identify appropriate screening and counseling while maintaining a therapeutic relationship with patients of all backgrounds. A risk-driven approach to sexual history taking and STI screening can help clinicians to tailor time and resources appropriately.

Most people infected with chlamydia are asymptomatic, leading to many missed diagnoses with symptom-driven testing.^8^ In addition, there is emerging evidence that extragenital infections of chlamydia may play an important role as reservoirs for ongoing transmission. A study conducted at an STI clinic in Missouri found that around one-third of chlamydia in females would have been missed if only endocervical samples had been collected, rather than extragenital testing including pharyngeal and rectal samples.^9^ While public health guidelines recognize the need for site-specific testing, resident education on this topic is limited, and providers are often uncomfortable with these expanded options.^10^

Untreated endocervical chlamydia infections in females can lead to pelvic inflammatory disease, infertility, and increased risk of HIV contraction.^11,12^ The CDC recommends screening all sexually active females under 25 years of age, and the USPSTF recommends annual chlamydia screening for all sexually active females ages 15–24 years.^2,13^ Despite this, chlamydia screening rates remain low nationally. According to the Healthcare Effectiveness Data and Information Set, among sexually active females between 16 and 24 years, only 48% of commercial preferred provider organization patients, 51% of commercial health maintenance organization patients, and 58% of Medicaid patients were screened for chlamydia in 2018.^11^

To determine whether our clinic followed CDC guidelines, we performed a baseline assessment of the resident and attending panels of internal medicine (IM) and med/peds providers utilizing the Epic SlicerDicer tool and found that only 31% of the 999 female patients between ages 13 and 24 seen over the last year in the primary care clinic had been screened for chlamydia. Patients between ages 20 and 24 were the most likely to be screened (38% screened). Although we were not able to query for whether patients were sexually active, based on national trends around 40%-50% of females ages 15–19 are sexually active (with higher rates at ages 20–24); these results showed a severe underutilization of screening of sexually active young females in our primary care clinic.^14^

To address our low screening rates, we created a sexual health curriculum for residents. We searched *MedEdPORTAL* and PubMed for sexual health and STI resources, using terms such as *sexual health, sexual history taking, sexually transmitted disease, sexually transmitted illnesses, sexual medicine, chlamydia,* and *chlamydia screening. MedEdPORTAL* has multiple publications related to teaching comprehensive sexual health for medical students but a lack of graduate medical education tools.^15–20^ These publications include an introduction to comprehensive sexual history taking, practicing LGBTQ inclusive sexual history questions with standardized patients, and discussion of provider bias and social determinants of sexual health for second- and third-year medical students. All are imagined for either preclinical or core clerkship education. Building off the introduction of sexual health in medical school, our curriculum serves as a refresher on principles of inclusive comprehensive sexual history taking, with additional instruction on tailoring history taking to a patient's individual risk. The curriculum also provides practical tips for more advanced sexual health in the clinical setting, including diagnosis and management of STIs (including secondary sites), expedited partner therapy (EPT), counseling, and indications for pre-exposure prophylaxis (PrEP). We believe by again addressing sexual health in first-year residency, learners can increase their comfort with comprehensive sexual histories and integrate appropriate sexual health interventions while they solidify their workflow as future clinicians. Additionally, by increasing comfort and knowledge of sexual health, we hope to improve chlamydia screening rates among young sexually active females.

To tailor our curriculum, we performed a precurriculum survey for IM and med/peds residents with a response rate of 21 out of 41 (51%). We used these survey data as a needs assessment. Only 29% of respondents reported feeling very or extremely comfortable with taking a sexual history overall, but respondents felt more comfortable asking about number of sexual partners (38%) or gender of partners (33%) as compared with nature of sexual contact (e.g., oral, anal, genital), which they were very or extremely comfortable with only 5% of the time. Regarding STI screening modalities, interns reported high or very high levels of comfort with urine sample for gonorrhea/chlamydia polymerase chain reaction (GC/CT PCR) testing (62%) and physician-collected cervical samples for GC/CT PCR (43%) but less comfort with rectal (2%), pharyngeal (10%), or patient-collected vaginal (10%) swabs for GC/CT PCR. There were low rates of feeling very or extremely comfortable with counseling for a positive GC/CT test (10%), providing EPT (5%), and providing safe sexual practice education to patients (14%). Respondents correctly answered 48% of knowledge-based questions related to STI screening and sexual health counseling.

Based on these results, we developed a curriculum for first-year IM and med/peds residents focused on advanced sexual history taking and indications and instructions regarding screening at secondary sites (pharyngeal and rectal). We also included education and resources for counseling on safe sex practices, for positive chlamydia testing, and for EPT.

## Methods

We developed our curriculum to fit within the preexisting, first-year ambulatory program for IM residents to help establish fundamentals of sexual history taking early in a learner's clinical practice. We secured protected time for this curriculum, leadership support and audiovisual equipment for the viewing of curricular material. This project, including precurriculum and postcurriculum surveys, was discussed with the quality improvement board at the University of Chicago, and it was decided that the project fell under quality improvement and did not require institutional review board approval. We initially planned for the curriculum to take place in a large classroom with tables arranged for group discussion and scheduled it for spring 2020. Due to the ongoing COVID-19 pandemic, implementation had to be adapted to an online platform. We used Zoom's screen share feature for slides and its chat function with directed questions to promote an interactive learning environment. The curriculum presented here is suitable for both in-person and online education.

This curriculum was intended either as an independent curriculum or as an addition to an existing women's health or ambulatory program. Although our target audience was first-year IM residents, the curriculum could be adapted for use with advanced medical students, faculty, or residents of other primary care–based specialties, such as family medicine, pediatrics, or OB/GYN. The curriculum assumed the learners had a basic understanding of sexual history taking and clinical testing for STIs, such as physician-collected cervical samples for GC/CT PCR, although some of these principles were reviewed. The facilitator role was intended for a core faculty member, a chief or senior resident with sexual health expertise. The facilitator leading the curriculum ideally had experience with the resident clinic to be better able to guide learners with practicalities such as placement of equipment and how to best coordinate with other staff members about obtaining and properly submitting samples for STIs.

Based on precurriculum survey data from first-year IM residents, we tailored our curriculum to more advanced topics such as efficient comprehensive sexual history taking, how to screen secondary sites for STIs (pharyngeal and rectal), and appropriate sexual health counseling. The curriculum consisted of a case-based didactic teaching session (Appendix A) offering examples of screening and counseling to be provided to different patient populations. The session took approximately 60 minutes. We encouraged resident participation throughout the session. Residents were also provided with a sexual health pocket guide (Appendix B) at the beginning of the session for reference during the session and future clinical encounters. The algorithm for comprehensive sexual history taking, which tailored questions based on patient characteristics for more efficient sexual history taking, was adapted from tool kits developed by the CDC and the National LGBT Health Education Center.^12,21^

To measure and trend trainee comfort, knowledge, and attitude regarding sexual history taking and STI screening, we conducted postcurriculum assessments for comparison with our precurriculum surveys (Appendix C). We compared cohorts using a two-sample *t* test assuming equal variance. To increase participation, we ended the didactic curriculum slightly early and allowed 5–10 minutes for survey completion directly following the end of the session. Although the survey was optional, we include it here since it serves as a tool to measure the effectiveness of a particular lecture with various learners.

## Results

We administered a precurriculum survey to IM and med/peds PGY 1 residents in spring 2019 and received responses from 21 out of 41 (51%). After curriculum development, we implemented our curriculum in fall 2020 and administered postcurriculum surveys to the incoming PGY 1 IM and med/peds class. We received 19 out of 33 responses (58%). Comparing the precurriculum and postcurriculum cohorts, we found no significant differences between them (Table 1).

**Table 1.**
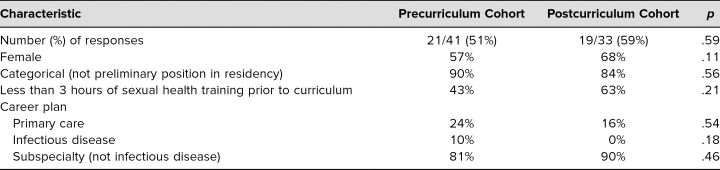
Pre- and Postcurriculum Cohort Comparison

**Figure 1.**
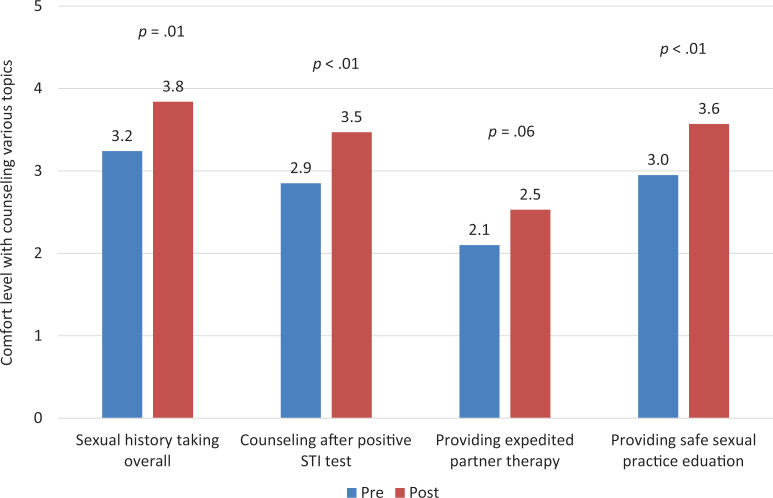
Comfort level of respondents with counseling various topics, precurriculum (*n* = 21) and postcurriculum (*n* = 19). Comfort was rated by respondents on a 5-point Likert scale (1 = *not at all,* 2 = *slightly,* 3 = *moderately,* 4 = *very,* 5 = *extremely*). Abbreviation: STI, sexually transmitted infection.

We assessed self-reported comfort level with counseling using a 5-point Likert scale (1 = *not at all,* 2 = *slightly,* 3 = *moderately,* 4 = *very,* 5 = *extremely*) for multiple areas related to sexual health. Following our curriculum, we found significant improvement in comfort with sexual history taking overall (*M*s: pre = 3.2, post = 3.8, *p* = .01), counseling after positive STI test (*M*s: pre = 2.9, post = 3.5, *p* < .01), and providing safe sexual practice education (*M*s: pre = 3.0, post = 3.6, *p* <.01). There was no significant change in comfort with providing EPT (*M*s: pre = 2.1, post = 2.5, *p* = .06; Figure 1).

**Figure 2.**
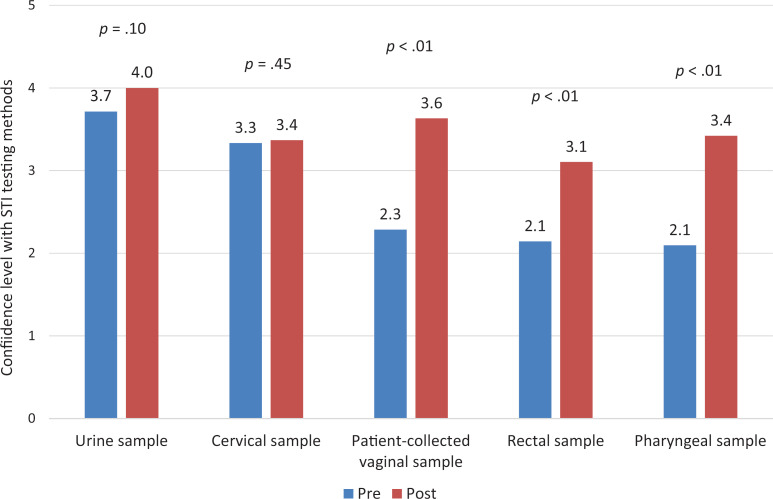
Confidence level of respondents with site-specific testing for STI screening, precurriculum (*n* = 21) and postcurriculum (*n* = 19). Confidence level was rated by respondents on a 5-point Likert scale (1 = *very low,* 2 = *low,* 3 = *moderately,* 4 = *high,* 5 = *very high*). Abbreviation: STI, sexually transmitted infection.

We assessed self-reported confidence with collecting site-specific testing using a 5-point Likert scale (1 = *very low,* 2 = *low,* 3 = *moderately,* 4 = *high,* 5 = *very high*). We found that confidence levels improved for patient-collected vaginal swabs (*M*s: pre = 2.3, post = 3.6, *p* < .01), rectal swabs (*M*s: pre = 2.1, post = 3.1, *p* < .01), and pharyngeal swabs (*M*s: pre = 2.1, post = 3.4, *p* < .01). We found no significant increase in confidence with urine sample (*M*s: pre = 3.7, post = 4.0, *p* = .10) or cervical swabs (*M*s: pre = 3.3, post = 3.4, *p* = .45). These methods were minimally addressed in the curriculum given high levels of confidence on the precurriculum surveys (Figure 2).

Respondents improved on knowledge questions, with percentage correct increasing from 48% to 78% postcurriculum. There was an increase in percentage correct in concepts related to asymptomatic females such as screening (pre = 67%, post = 90%, *p* = .04), specimen collection (pre = 24%, post = 74%, *p* = .01), and rescreening timing if chlamydia positive (pre = 19%, post = 58%, *p* = .01). We also found improvement in percentage correct for questions related to history taking (pre = 81%, post = 100%, *p* = .02) and specimen collection (pre = 19%, post = 84%, *p* < .01), as well as a question related to informing partners and offering EPT to chlamydia-positive patients (pre = 48%, post = 90%, *p* < .01). We found no significant improvement in correct answers for questions related to screening timing for symptomatic females (pre = 86%, post = 79%, *p* = .29) and rescreening for chlamydia-positive males (pre = 43%, post = 47%, *p* = .39; Table 2). We asked our respondents about the usefulness of the pocket card as a companion to the curriculum and found that 73% reported they were likely to use the pocket card. Respondents thought the pocket card would be helpful with offering risk-based STI screening (78%), identifying patients who needed screening including extragenital collection (63%), and identifying patients appropriate for PrEP (63%).

**Table 2.**
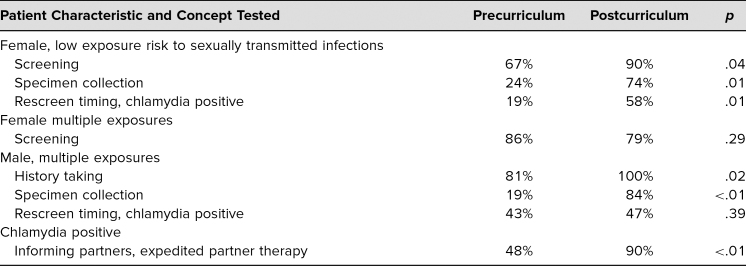
Clinical Management Questions: Percentage Correct

## Discussion

Although many residents enter residency with some basic knowledge of sexual history taking and STI screening, there is little uniformity in sexual health education in either undergraduate or graduate medical education. These concepts should be built upon early in residency education to solidify the importance of sexual health in patients’ overall health. Our initiative assessed baseline knowledge and comfort of the first-year residents at our institution and built upon those concepts for a more comprehensive sexual health. Through this curricular intervention, we were able to show improvement in self-reported levels of comfort with sexual health counseling and confidence with STI testing methods, as well as improved percentage correct on clinical management questions related to sexual health.

There were several limitations to our curriculum. Responses for both precurriculum and postcurriculum assessments were close to 50%, lower than we had hoped, perhaps due to lack of comfort with sexual health as well as time constraints. During instruction, we found that some residents were much less comfortable with sexual health than others and sometimes required more prompting for participation. We were also limited in terms of time and number of sessions; we feel that a more longitudinal sexual health curriculum would help solidify some more complex sexual health topics. For those residents interested, we often moved questions to outside of curriculum time or offered supplemental resources.

The curriculum was also limited to a didactic, case-based format. This format reaches some learners for some concepts, but videos, standardized patients, or patient-centered education in clinic can additionally bolster complex topics. For example, although responses to knowledge-based questions improved significantly related to EPT for sexual partners following positive STI testing, reported comfort with EPT counseling did not significantly improve. This indicates a need for additional education on the communication skills needed for EPT counseling. More counseling-based concepts, such as EPT, may be difficult to completely cover in a didactic session and instead may be better addressed by standardized patient encounters and/or role-playing exercises. It may be of interest to reevaluate this endpoint after interns have had more exposure to ambulatory clinical practice and, ideally, a chance to provide EPT in the clinic. Additionally, we originally planned to compare chlamydia screening rates pre- and postcurriculum. However, due to the baseline rates obtained prior to the COVID-19 epidemic and increased use of telehealth, we deferred comparison of this iteration of our curriculum given the high likelihood of confounding variables.

Sexual health is a complex topic that requires education and practice for physician comfort. We imagine our curriculum as part of a larger continuing education for sexual health that begins in medical school and continues throughout an individual's career. Health care providers should continue to learn more about inclusive, comprehensive care from continuing medical education as well as from patients themselves. This curriculum provides learners with a risk-based framework for discussing sexual history and screening for STIs, as well as direction on treatment of STIs. We have demonstrated that this curriculum can improve learners’ knowledge of key sexual health topics and comfort with history taking, screening and treatment of STIs, and sexual health counseling.

## Appendices


Sexual Health Lecture.pptxSexual Health Pocket Card.pptxSexual Health Pre- and Postcurriculum Survey.docx
*All appendices are peer reviewed as integral parts of the Original Publication.*
