# Supplementary material for: An Advanced, Risk-Driven Sexual Health Curriculum for First-Year Internal Medicine Residents
Source: MedEdPORTAL. 2022 Dec 9;18:11287. doi: 10.15766/mep_2374-8265.11287 (PMC9732138; doi:10.15766/mep_2374-8265.11287)
Supplement: Supplementary file 1 — Sexual Health Lecture.pptxSexual Health Pocket Card.pptxSexual Health Pre- and Postcurriculum Survey.docx [file mep_2374-8265.11287-s001.zip › B. Sexual Health Pocket Card.pptx]

## Slide 1
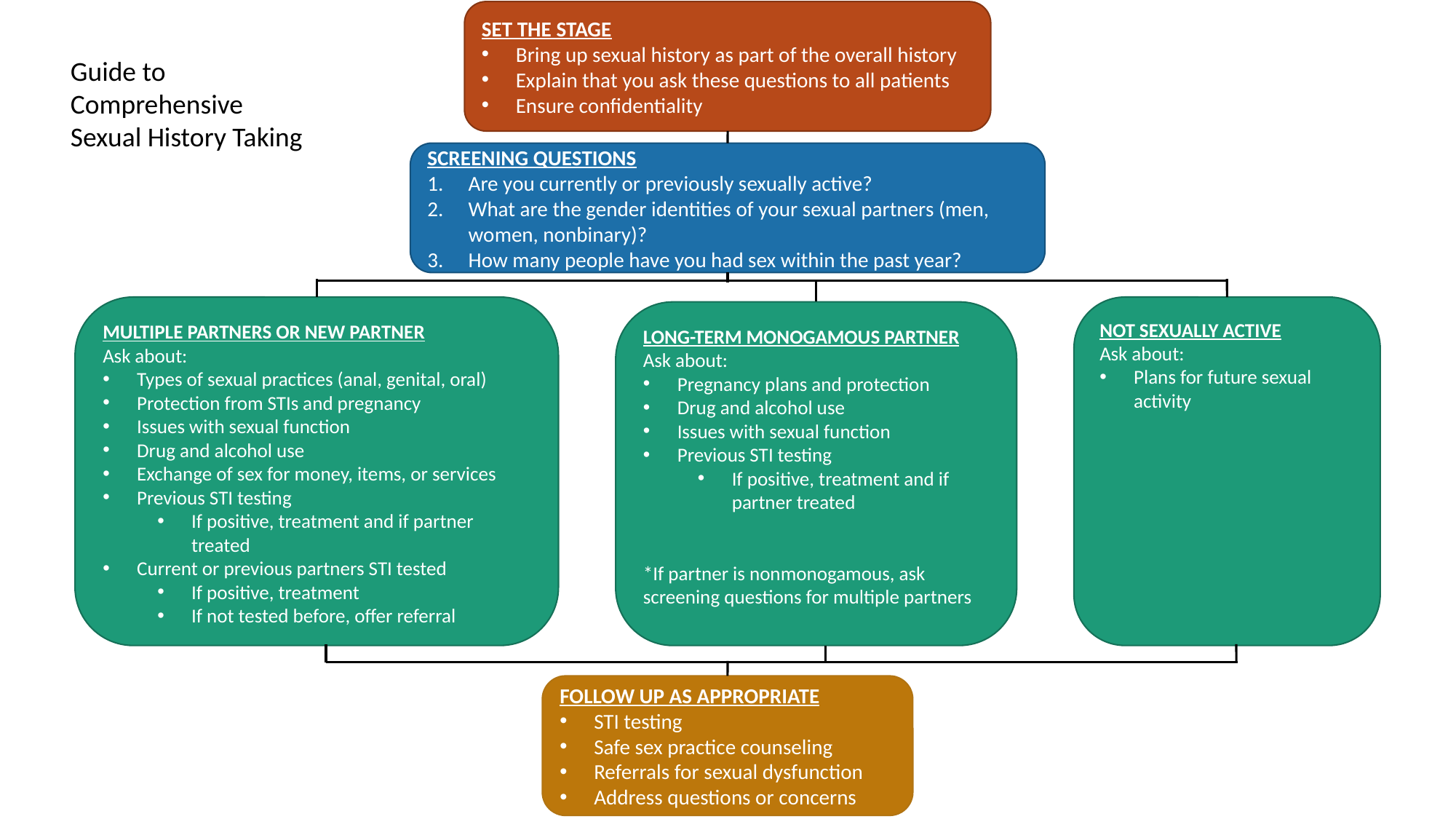

SET THE STAGE
Bring up sexual history as part of the overall history
Explain that you ask these questions to all patients
Ensure confidentiality
SCREENING QUESTIONS
Are you currently or previously sexually active?
What are the gender identities of your sexual partners (men, women, nonbinary)?
How many people have you had sex within the past year?
MULTIPLE PARTNERS OR NEW PARTNER
Ask about:
Types of sexual practices (anal, genital, oral)
Protection from STIs and pregnancy
Issues with sexual function
Drug and alcohol use
Exchange of sex for money, items, or services
Previous STI testing
If positive, treatment and if partner treated
Current or previous partners STI tested
If positive, treatment
If not tested before, offer referral
NOT SEXUALLY ACTIVE
Ask about:
Plans for future sexual activity
LONG-TERM MONOGAMOUS PARTNER
Ask about:
Pregnancy plans and protection
Drug and alcohol use
Issues with sexual function
Previous STI testing
If positive, treatment and if partner treated
*If partner is nonmonogamous, ask screening questions for multiple partners
FOLLOW UP AS APPROPRIATE
STI testing
Safe sex practice counseling
Referrals for sexual dysfunction
Address questions or concerns
Guide to Comprehensive Sexual History Taking

## Slide 2
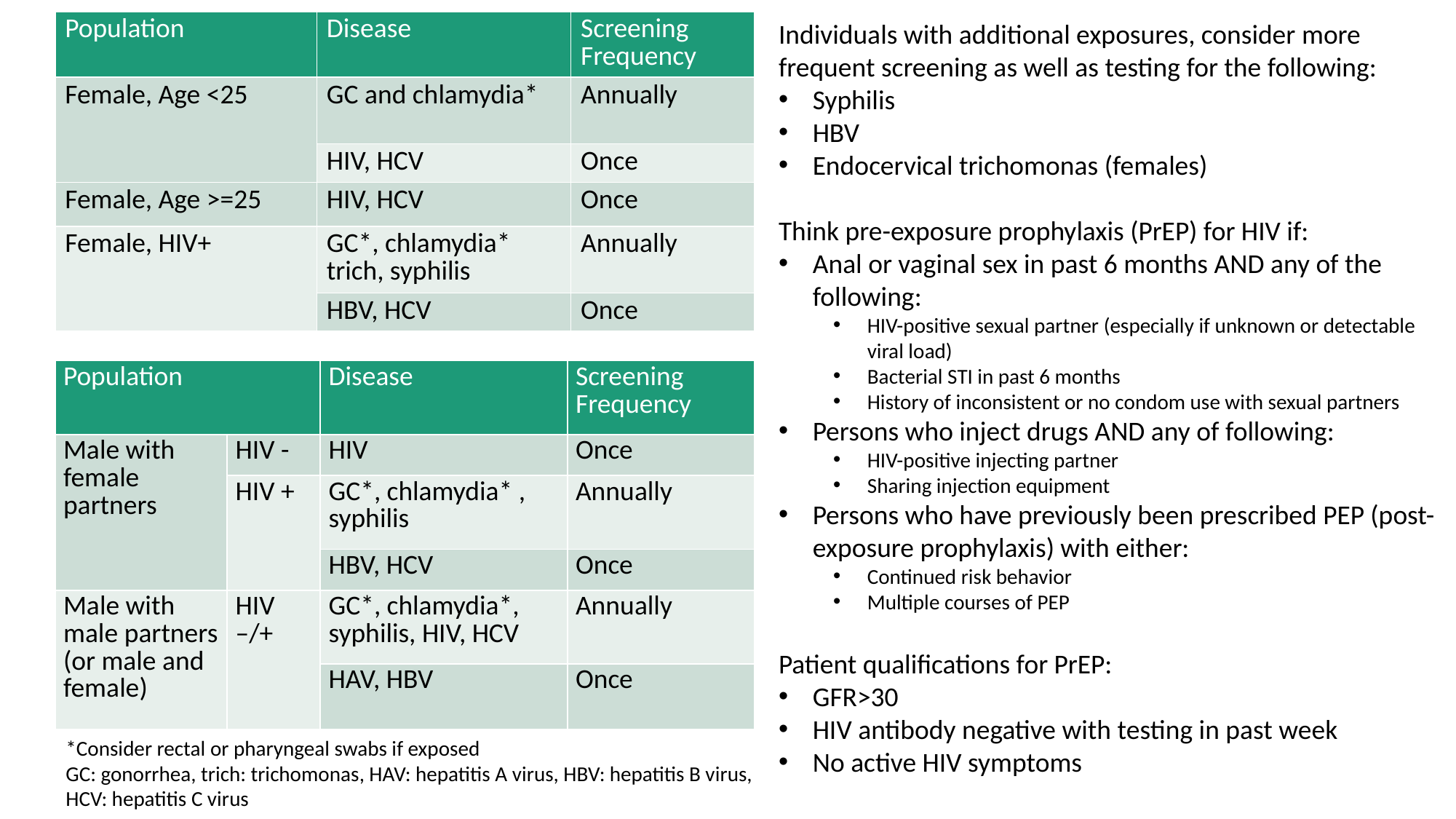

| Population | Disease | Screening Frequency |
| --- | --- | --- |
| Female, Age <25 | GC and chlamydia\* | Annually |
| | HIV, HCV | Once |
| Female, Age >=25 | HIV, HCV | Once |
| Female, HIV+ | GC\*, chlamydia\* trich, syphilis | Annually |
| | HBV, HCV | Once |
Individuals with additional exposures, consider more frequent screening as well as testing for the following:
Syphilis
HBV
Endocervical trichomonas (females)
Think pre-exposure prophylaxis (PrEP) for HIV if:
Anal or vaginal sex in past 6 months AND any of the following:
HIV-positive sexual partner (especially if unknown or detectable viral load)
Bacterial STI in past 6 months
History of inconsistent or no condom use with sexual partners
Persons who inject drugs AND any of following:
HIV-positive injecting partner
Sharing injection equipment
Persons who have previously been prescribed PEP (post-exposure prophylaxis) with either:
Continued risk behavior
Multiple courses of PEP
Patient qualifications for PrEP:
GFR>30
HIV antibody negative with testing in past week
No active HIV symptoms
| Population | | Disease | Screening Frequency |
| --- | --- | --- | --- |
| Male with female partners | HIV - | HIV | Once |
| | HIV + | GC\*, chlamydia\* , syphilis | Annually |
| | | HBV, HCV | Once |
| Male with male partners (or male and female) | HIV –/+ | GC\*, chlamydia\*, syphilis, HIV, HCV | Annually |
| | | HAV, HBV | Once |
*Consider rectal or pharyngeal swabs if exposed
GC: gonorrhea, trich: trichomonas, HAV: hepatitis A virus, HBV: hepatitis B virus, HCV: hepatitis C virus
