# Supplementary material for: An Advanced, Risk-Driven Sexual Health Curriculum for First-Year Internal Medicine Residents
Source: MedEdPORTAL. 2022 Dec 9;18:11287. doi: 10.15766/mep_2374-8265.11287 (PMC9732138; doi:10.15766/mep_2374-8265.11287)
Supplement: Supplementary file 1 — Sexual Health Lecture.pptxSexual Health Pocket Card.pptxSexual Health Pre- and Postcurriculum Survey.docx [file mep_2374-8265.11287-s001.zip › C. Sexual Health Pre- and Postcurriculum Survey.docx]

**Sexual Health Pre- and Post-Survey**

This survey was created to gather information regarding your knowledge and experience with obtaining a sexual history and counseling in regards to sexually transmitted infections during your **PRIMARY CARE** visits. Survey should take 5-10 minutes to complete

**Section 1:**

The following are related to your knowledge and comfort with taking a sexual history.

We define sexual history as a discussion of sexual health issues pertaining to a particular patient such as risk factors for sexually transmitted infections, reproductive disorders, fertility, and avoidance of unwanted pregnancies.

1. Within your residency education for primary care, how important to you are skills related with sexual health?
   1. Not at all
   2. Slightly
   3. Moderately
   4. Very
   5. Extremely
2. How important do you think patients perceive their own sexual health?
   1. Not at all
   2. Slightly
   3. Moderately
   4. Very
   5. Extremely
3. How often in future clinic appointments do predict you will ask about sexual history (any component)?
   1. Rarely (<20%)
   2. Sometimes (21-49%)
   3. Often (50-79%)
   4. Routinely (>80%)
4. Which of the following are essential elements of sexual history as defined by the CDC? Identify all the apply.
   1. Partners (number, genders)
   2. Practices (vaginal, oral, anal intercourse)
   3. Privacy (respect for patient’s cultural values and avoid any patient discomfort by asking only targeted questions about sexual history)
   4. Protection from STDs (condoms, PREP, ect)
   5. Pornography (do they view pornography, how often and what type of content)
   6. Past History of STDs (diagnosis and treatment)
   7. Prevention of unplanned pregnancy (review contraception)
5. A 45 year-old male comes in for his annual exam. He is married to a female partner for the last 10 years but upon asking a sexual history, you learn he has had multiple sexual partners, male and female, over the last 12 months. What is your next step?
   1. Ask about specific sites of intercourse to guide where to obtain screening samples for gonorrhea and chlamydia
   2. Find out if his wife knows. She’s a patient in the clinic and it is your responsibility to ensure she also receives appropriate screening.
   3. You already have enough information to direct your screening.
   4. Discuss the importance of monogamy from a health standpoint using neutral language
6. How confident do you feel taking a sexual history overall?
   1. Not at all
   2. Slightly
   3. Moderately
   4. Very
   5. Extremely
7. Please rate your comfort level in obtaining the following aspects of a sexual history:

| **Aspect of Sexual History** | **Very low** | **Low** | **Moderate** | **High** | **Very high** |
| --- | --- | --- | --- | --- | --- |
| If sexually active | 1 | 2 | 3 | 4 | 5 |
| Number of sexual partners | 1 | 2 | 3 | 4 | 5 |
| Biologic sex of sexual partners | 1 | 2 | 3 | 4 | 5 |
| A patient’s sexual concerns | 1 | 2 | 3 | 4 | 5 |
| Nature of sexual contact (e.g. oral, genital, anal) | 1 | 2 | 3 | 4 | 5 |
| Use of protection from sexually transmitted illnesses | 1 | 2 | 3 | 4 | 5 |
| Past history of sexually transmitted illnesses | 1 | 2 | 3 | 4 | 5 |
| Family Planning | 1 | 2 | 3 | 4 | 5 |
| Partner violence | 1 | 2 | 3 | 4 | 5 |

1. Please estimate the amount of training you have previously received in regards to sexual history taking?
   1. None
   2. Less than 1 hour
   3. Less than 3 hours
   4. More than 3 hours
2. Which of the following is among the reasons preventing you from taking a sexual history in clinic? Select all that apply.
   1. Sexual History not pertinent to this patient’s illness
   2. Patient did not bring it up
   3. Feels too intrusive into patient’s personal life
   4. I do not know the STI screening guidelines to use as a guide for sexual history review
   5. There is no clinical reminder within the electronic medical system
   6. Lack of comfort with how to take a sexual history
   7. Lack of comfort with how to counsel patients
   8. Lack of resources for patients on safe sex practices
   9. Personal embarrassment
   10. Time constraints
   11. I do not know the guidelines regarding sexual history taking
   12. There is nothing preventing me from taking a sexual history in clinic
   13. Other: _______________________

**Section 2:** The following are related to your knowledge and comfort with sexually transmitted infections (STIs) which include HIV, syphilis, gonorrhea, chlamydia, HSV, HPV and trichomonas.

1. Cindy, a 20 year-old female graduate student in physics presents for her school mandated physical. You are reviewing her health maintenance. Upon review of her sexual history, you find she was previously sexually active but has been abstinent for past 3 months. She denies vaginal discharge, dysuria, rash or other genitourinary complaints. Which of the following should be obtained?
   1. Chlamydia screening
   2. Trichomonas screening
   3. HSV screening
   4. Syphilis screening
   5. HPV screening
   6. None of the above; she is not currently sexually active
2. For chlamydia screening, which of the following is the preferred manner of collection of samples:
   1. Urine sample for chlamydia PCR testing
   2. Patient-collected vaginal self-swab for chlamydia PCR testing
   3. Physician-collected cervical swab for chlamydia PCR testing
   4. Either B or C are equally preferred
   5. All of the above are equally preferred
3. Cindy’s chlamydia test is positive. You prescribe azithromycin for Cindy. What else should you do?
   1. Ask about marital status
   2. Order a trichomonas test; the two diseases are associated
   3. Schedule follow-up appointment in 1 month for retesting
   4. Schedule follow-up appointment in 3 months for retesting
   5. Rescreen in 1 year for chlamydia
4. Which of the following is an appropriate option for informing Cindy’s partners?
   1. You do not need to discuss this because the laboratory automatically reports to the state which will contact the partners for her.
   2. Advise her to contact either her most recent partner or all of her partners over the last 60 days, whichever is more inclusive, and inform them to be tested
   3. If she still has contact with her recent partner provide Cindy with an azithromycin script for her partner
   4. Only A is correct
   5. B and C are correct
5. A 19 year old female presents complaining of pelvic pain for past week which has been persistent and slowly worsening. She does have some associated nausea but denies vomiting or change in bowel movements. She is sexually active and was recently tested for STIs 6 months ago, including HIV, gonorrhea, and chlamydia. She has a history of a surgical abortion 1 year prior. Which of the following should you include during your visit?
   1. Hemoglobin A1c; this could be gastroparesis
   2. Urine sample for chlamydia PCR testing
   3. Vaginal self-collected swab for chlamydia PCR testing
   4. Ultrasound for ovarian torsion
   5. Endocervical chlamydia swab obtained during a pelvic exam
   6. Refer to gynecology; this could be related with her previous surgical abortion
6. A 41 year old male presents who has a history of 10 male sexual partners over the last year. He participates in both insertive and receptive oral intercourse as well as receptive anal intercourse. After practicing shared-decision making, you elect to screen for HIV, syphilis, chlamydia and gonorrhea. Which testing do you order (check all that apply)?
   1. Urine sample for chlamydia and gonorrhea PCR testing; you make sure to explain how to get a mid-stream collection
   2. Urine sample for chlamydia and gonorrhea PCR testing; you make sure to explain how to get a first-catch urine sample
   3. Rectal swab for gonorrhea and chlamydia PCR testing
   4. Pharyngeal swab for gonorrhea and chlamydia PCR testing
7. (follow up to #7) Presuming his testing today is negative when should the patient be rescreened for STDs?
   1. In 1 year per the CDC recommendations for asymptomatic individuals
   2. Screen every 3 to 6 months for HIV, syphilis, chlamydia and gonorrhea
   3. Testing for gonorrhea and chlamydia only in accordance with CDC guidelines – HIV and syphilis are recommended at most annually for asymptomatic individuals
   4. Test for HSV antibody in the serum – he is high risk and this test should have been ordered at initial PCP visit and was missed
8. What is your comfort level with appropriate use and collection of the following STI screening techniques? *gonorrhea/chlamydia abbreviated as GC/CT

|  | **Very low** | **Low** | **Moderate** | **High** | **Very high** |
| --- | --- | --- | --- | --- | --- |
| Urine sample for GC/CT PCR | 1 | 2 | 3 | 4 | 5 |
| Cervical GC/CT PCR | 1 | 2 | 3 | 4 | 5 |
| Vaginal swab for GC/CT PCR | 1 | 2 | 3 | 4 | 5 |
| Rectal GC/CT PCR | 1 | 2 | 3 | 4 | 5 |
| Pharyngeal PG/CT PCR | 1 | 2 | 3 | 4 | 5 |

1. Please rate your comfort level with providing counseling for positive testing for gonorrhea or chlamydia.
   1. Not at all
   2. Slightly
   3. Moderately
   4. Very
   5. Extremely
2. What is your comfort level with providing expedited partner therapy (treatment of STIs to partners of patients without a clinic visit)?
   1. Not at all
   2. Slightly
   3. Moderately
   4. Very
   5. Extremely
3. What is your comfort level in providing safe sexual practice eduation to patients, either verbally or in written form?
   1. Not at all
   2. Slightly
   3. Moderately
   4. Very
   5. Extremely

**FACILITATORS GUIDE:**

Correct answers for knowledge-based questions:

Section 1:

4. A, B, D, F, G

5. A (ask about site specific exposures to guide STI testing methods)

Section 2:

1. A (as sexually active female <25 years of age, she meets criteria for annual chlamydia screening)

2. D (urine samples for chlamydia PCR in women are less sensitive than vaginal or cervical swabs)

3. D (scheduled follow-up in 3 months for re-screening – not a test of cure but to ensure she did not contract again)

4. E (inform patient to inform their partners and prescribe expedited partner therapy for recent partner, if possible)

5. E (given pelvic pain with possible PID, needs pelvic exam in addition to STI testing)

6. B (first catch sample for urine more sensitive than mid stream catch), C (given history of receptive ana intercourse), D (given history of receptive oral intercourse)

7. B (given multiple sexually partners, should be considered for more frequent screening)
