# Supplementary material for: An Advanced, Risk-Driven Sexual Health Curriculum for First-Year Internal Medicine Residents
Source: MedEdPORTAL. 2022 Dec 9;18:11287. doi: 10.15766/mep_2374-8265.11287 (PMC9732138; doi:10.15766/mep_2374-8265.11287)
Supplement: Supplementary file 1 — Sexual Health Lecture.pptxSexual Health Pocket Card.pptxSexual Health Pre- and Postcurriculum Survey.docx [file mep_2374-8265.11287-s001.zip › A. Sexual Health Lecture.pptx]

## Slide 1
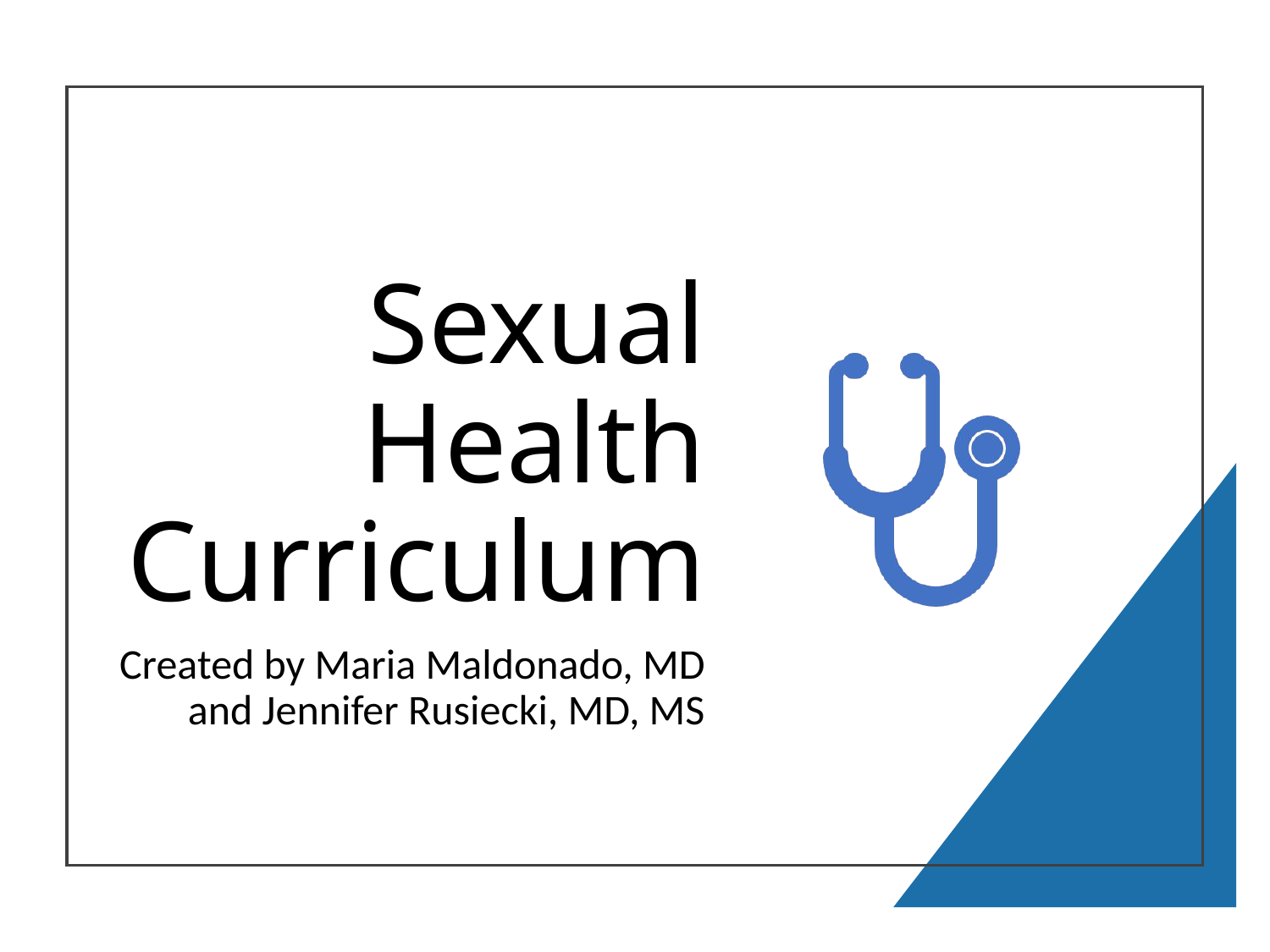

# Sexual Health Curriculum
Created by Maria Maldonado, MD and Jennifer Rusiecki, MD, MS

## Slide 2
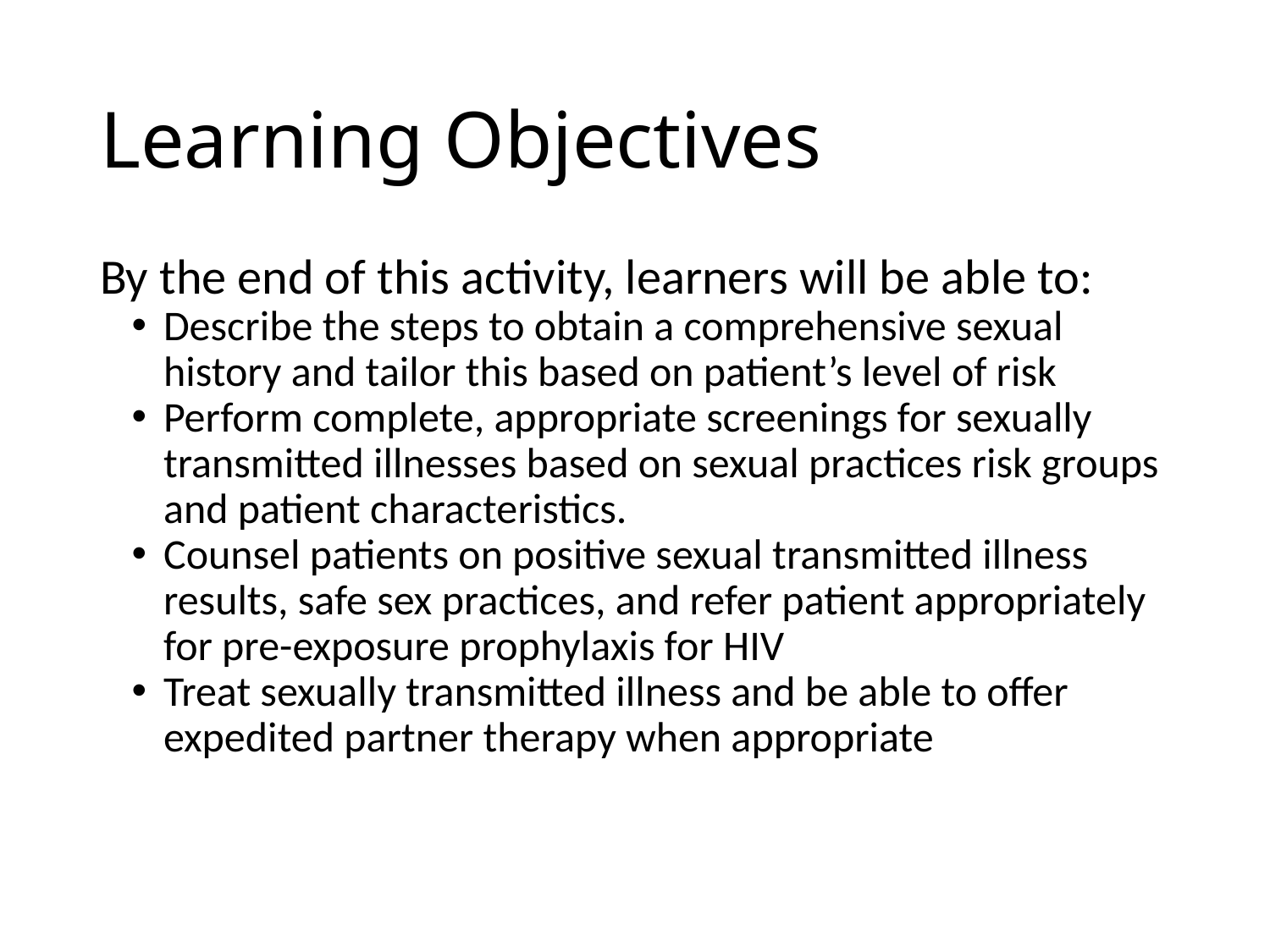

# Learning Objectives
By the end of this activity, learners will be able to:
Describe the steps to obtain a comprehensive sexual history and tailor this based on patient’s level of risk
Perform complete, appropriate screenings for sexually transmitted illnesses based on sexual practices risk groups and patient characteristics.
Counsel patients on positive sexual transmitted illness results, safe sex practices, and refer patient appropriately for pre-exposure prophylaxis for HIV
Treat sexually transmitted illness and be able to offer expedited partner therapy when appropriate

## Slide 3
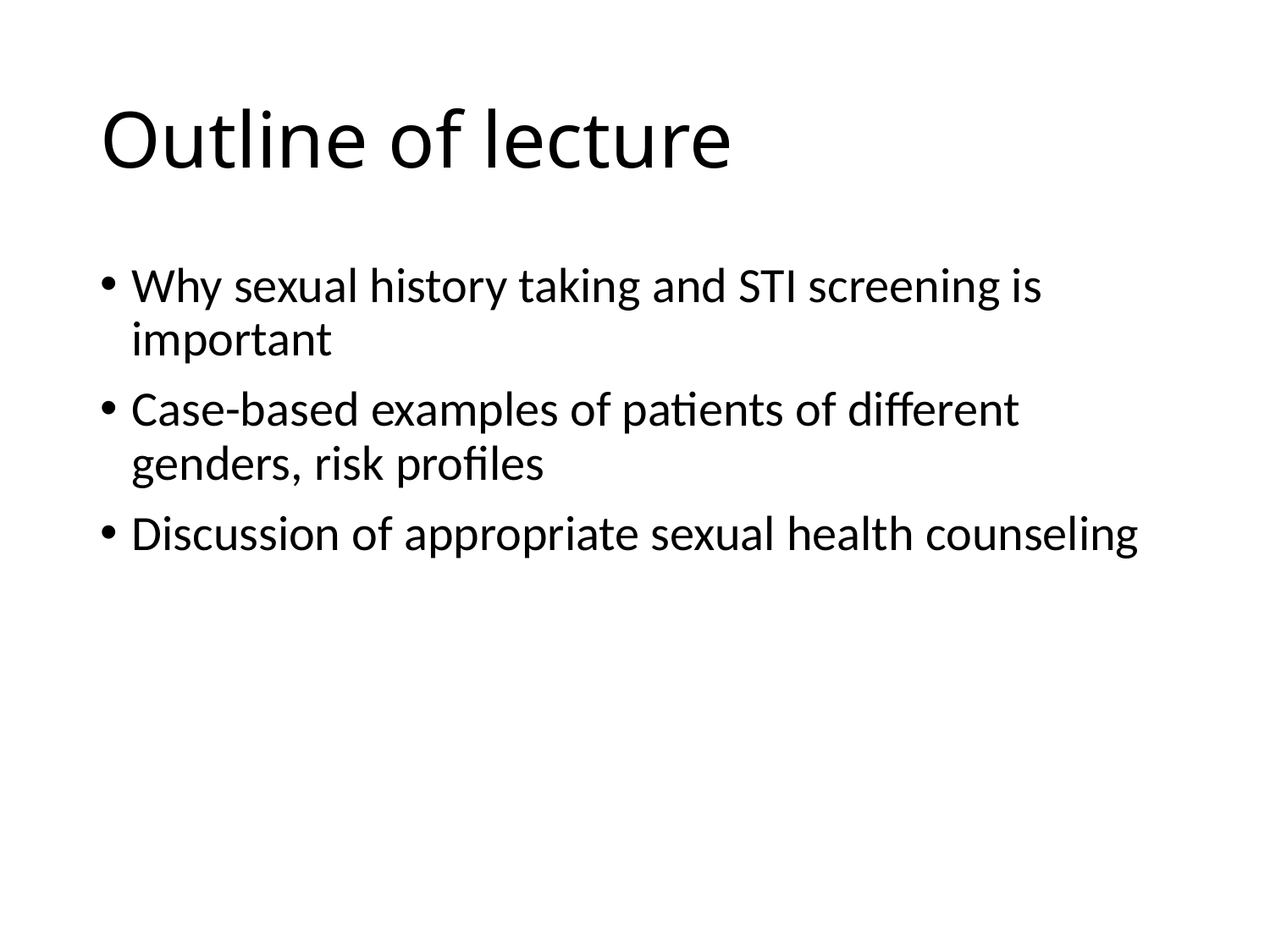

# Outline of lecture
Why sexual history taking and STI screening is important
Case-based examples of patients of different genders, risk profiles
Discussion of appropriate sexual health counseling

## Slide 4
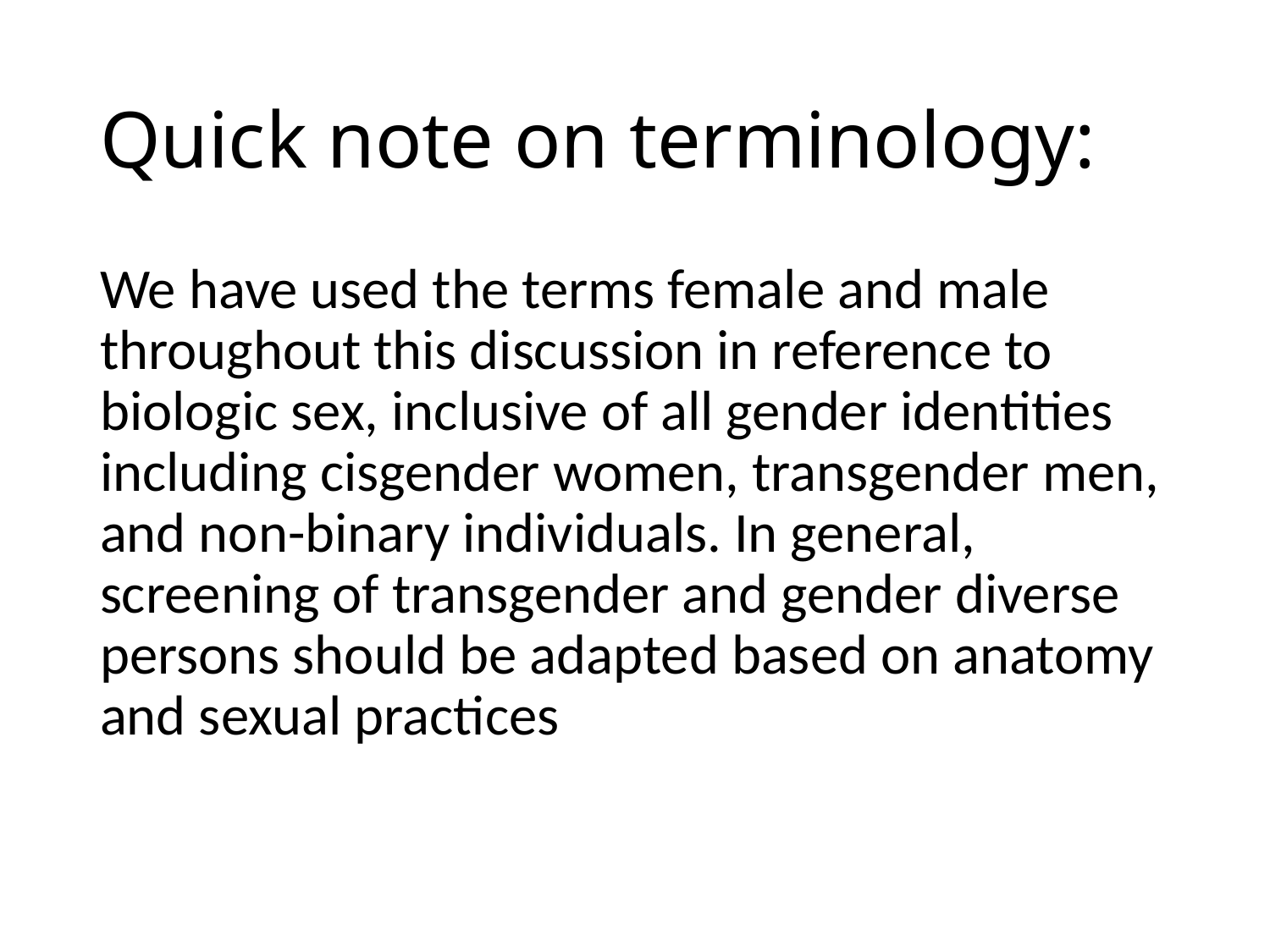

# Quick note on terminology:
We have used the terms female and male throughout this discussion in reference to biologic sex, inclusive of all gender identities including cisgender women, transgender men, and non-binary individuals. In general, screening of transgender and gender diverse persons should be adapted based on anatomy and sexual practices

## Slide 5
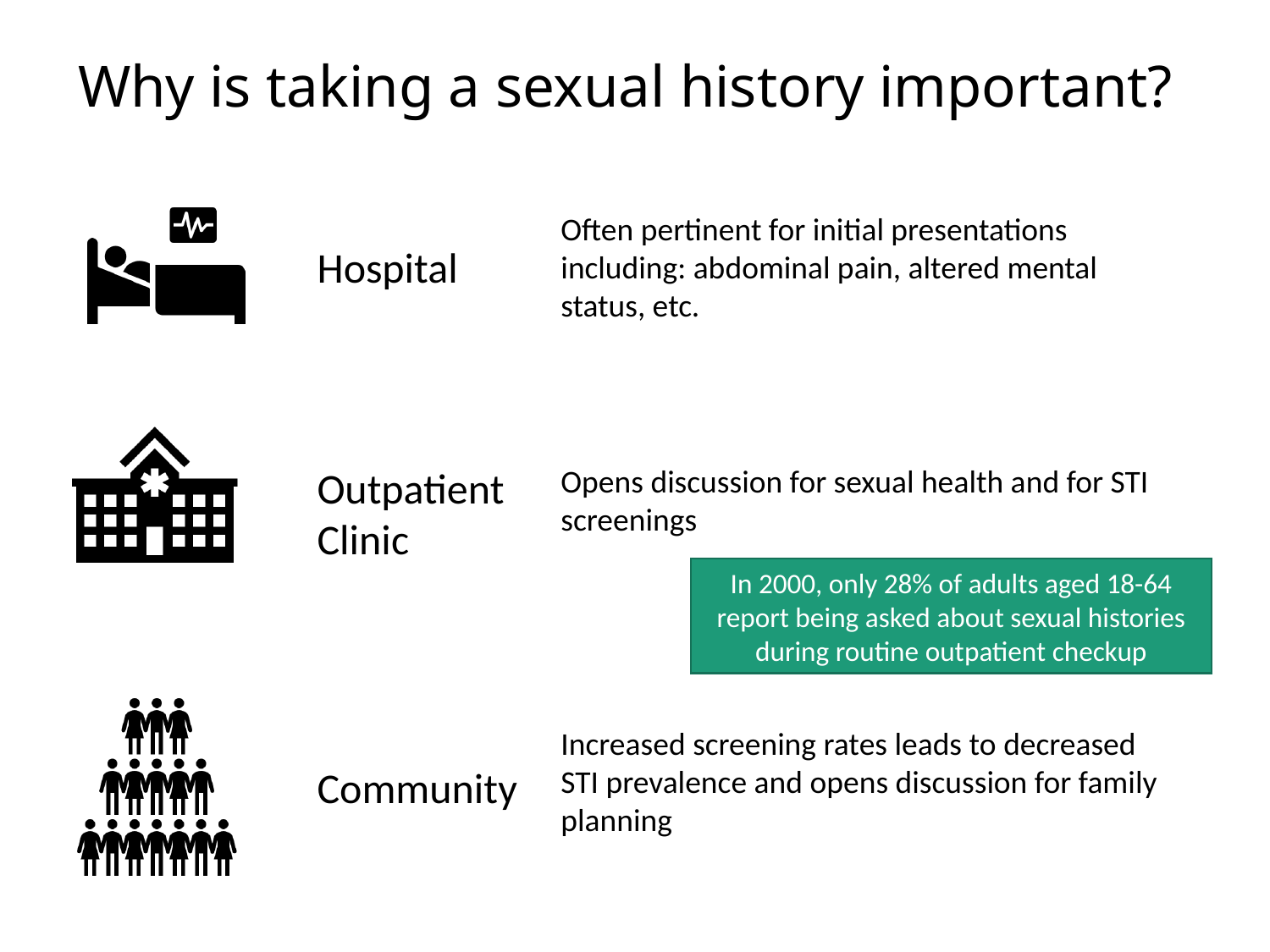

# Why is taking a sexual history important?
Often pertinent for initial presentations including: abdominal pain, altered mental status, etc.
Hospital
Outpatient Clinic
Opens discussion for sexual health and for STI screenings
In 2000, only 28% of adults aged 18-64 report being asked about sexual histories during routine outpatient checkup
Increased screening rates leads to decreased STI prevalence and opens discussion for family planning
Community

## Slide 6
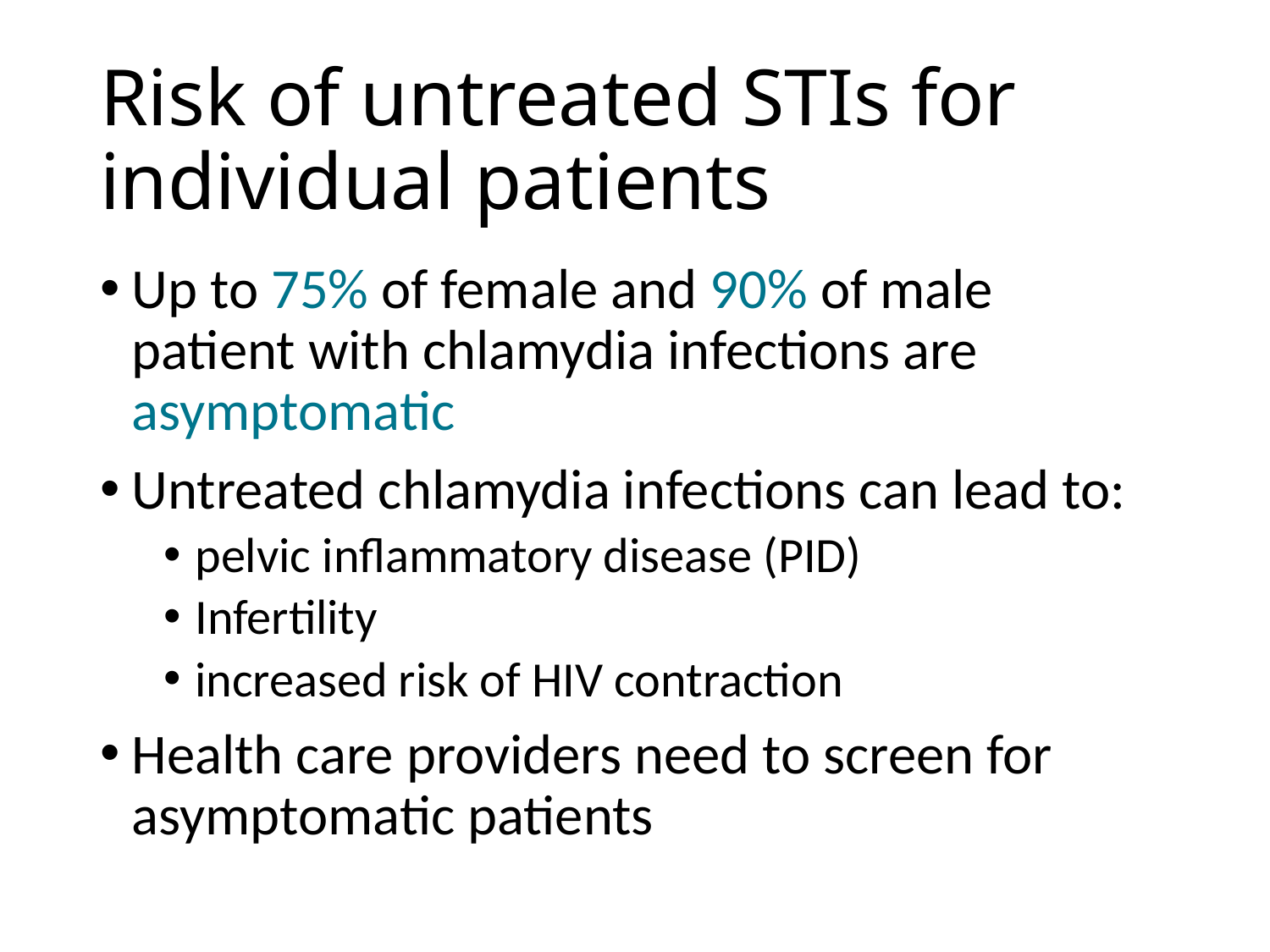

# Risk of untreated STIs for individual patients
Up to 75% of female and 90% of male patient with chlamydia infections are asymptomatic
Untreated chlamydia infections can lead to:
pelvic inflammatory disease (PID)
Infertility
increased risk of HIV contraction
Health care providers need to screen for asymptomatic patients

## Slide 7
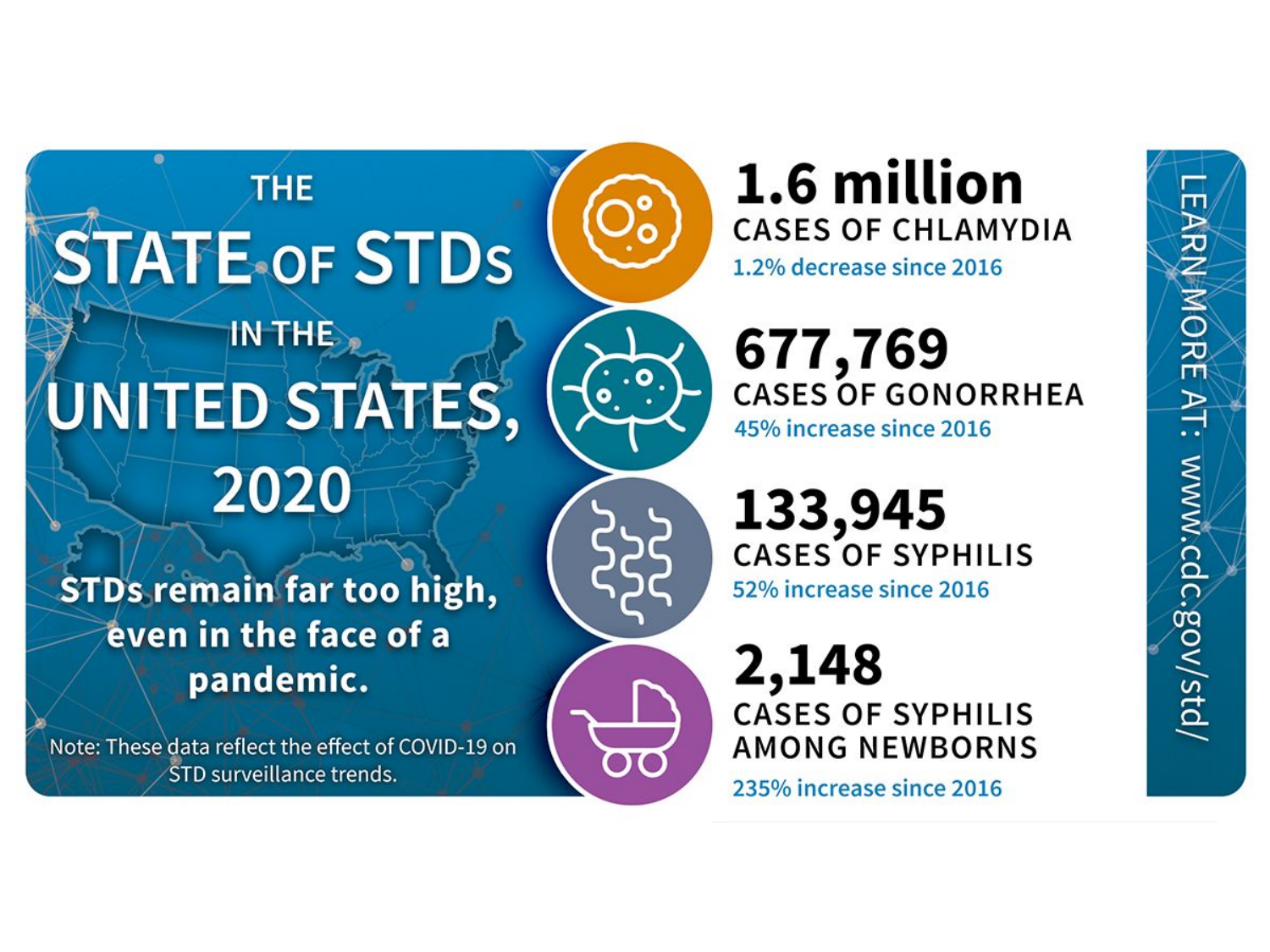

## Slide 8
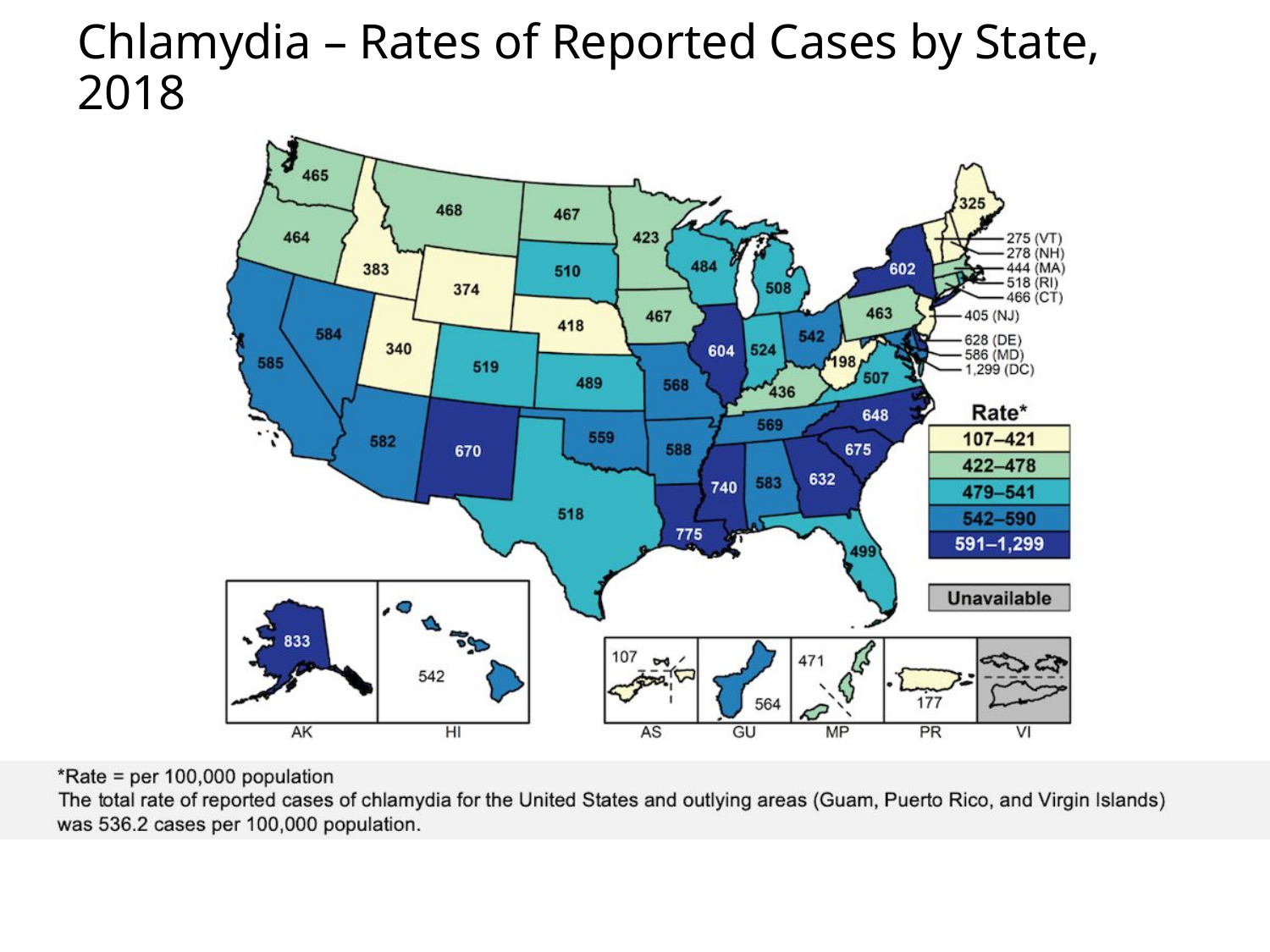

# Chlamydia – Rates of Reported Cases by State, 2018

## Slide 9
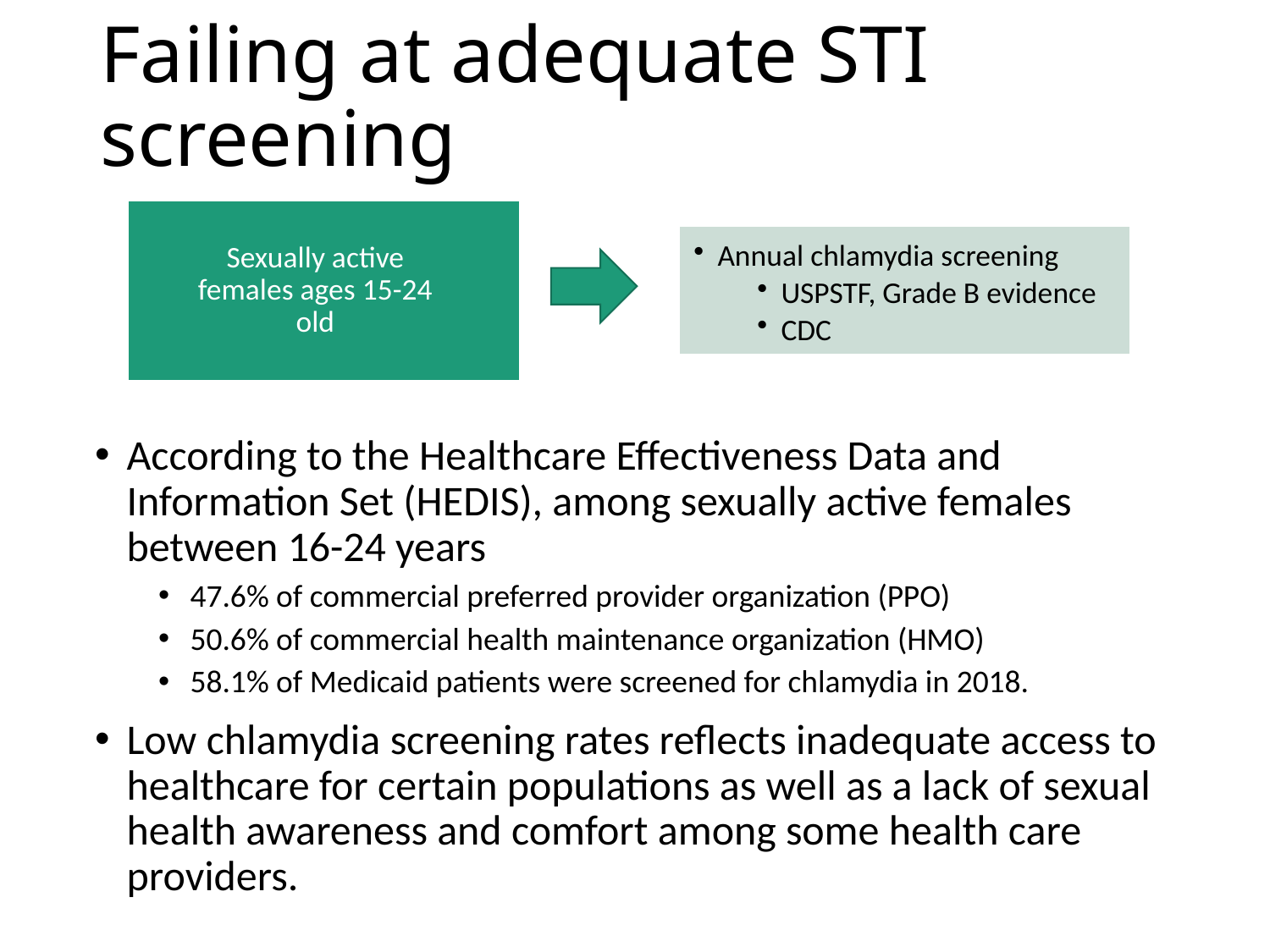

# Failing at adequate STI screening
Sexually active females ages 15-24 old
Annual chlamydia screening
USPSTF, Grade B evidence
CDC
According to the Healthcare Effectiveness Data and Information Set (HEDIS), among sexually active females between 16-24 years
47.6% of commercial preferred provider organization (PPO)
50.6% of commercial health maintenance organization (HMO)
58.1% of Medicaid patients were screened for chlamydia in 2018.
Low chlamydia screening rates reflects inadequate access to healthcare for certain populations as well as a lack of sexual health awareness and comfort among some health care providers.

## Slide 10
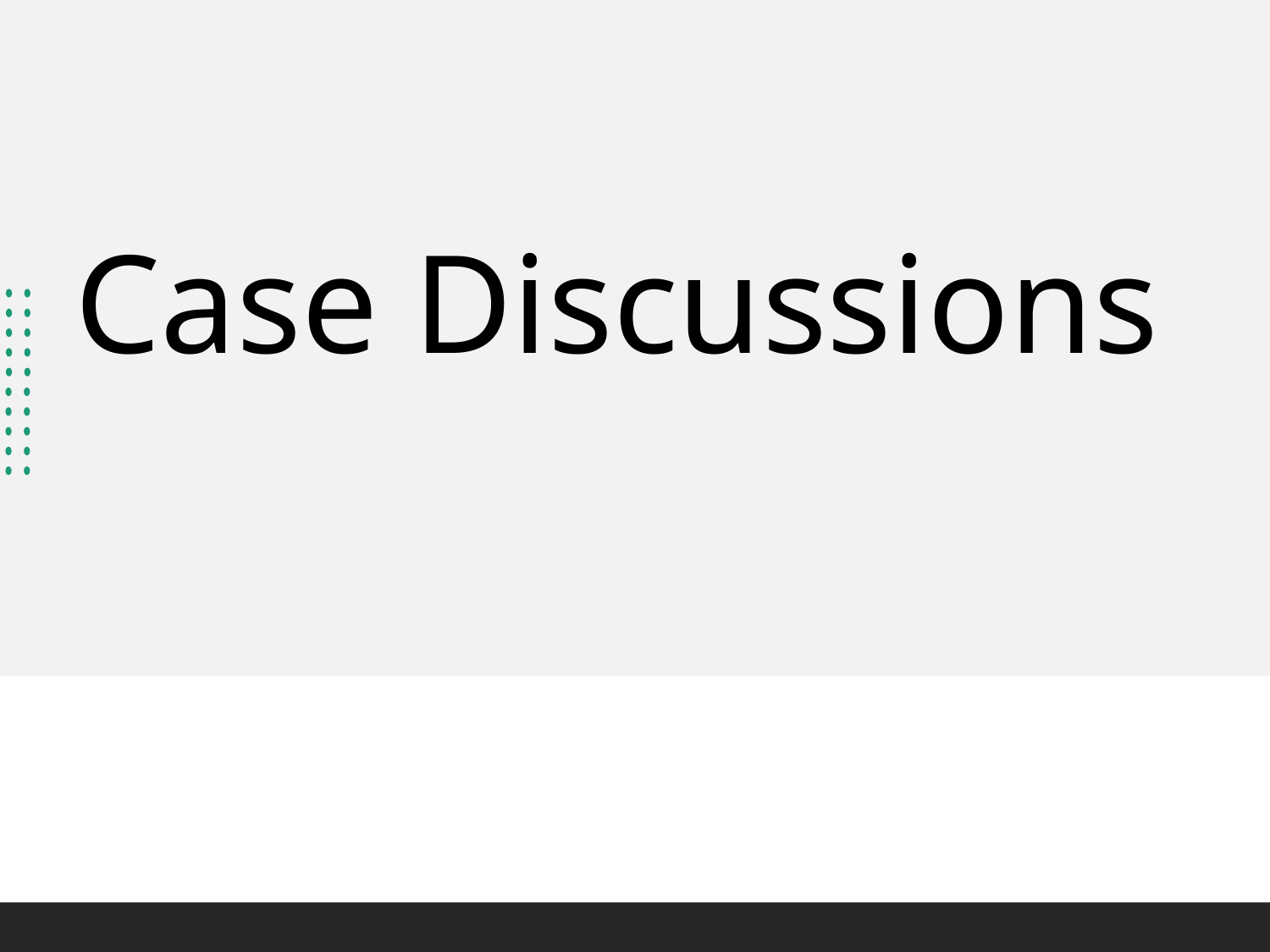

# Case Discussions

## Slide 11
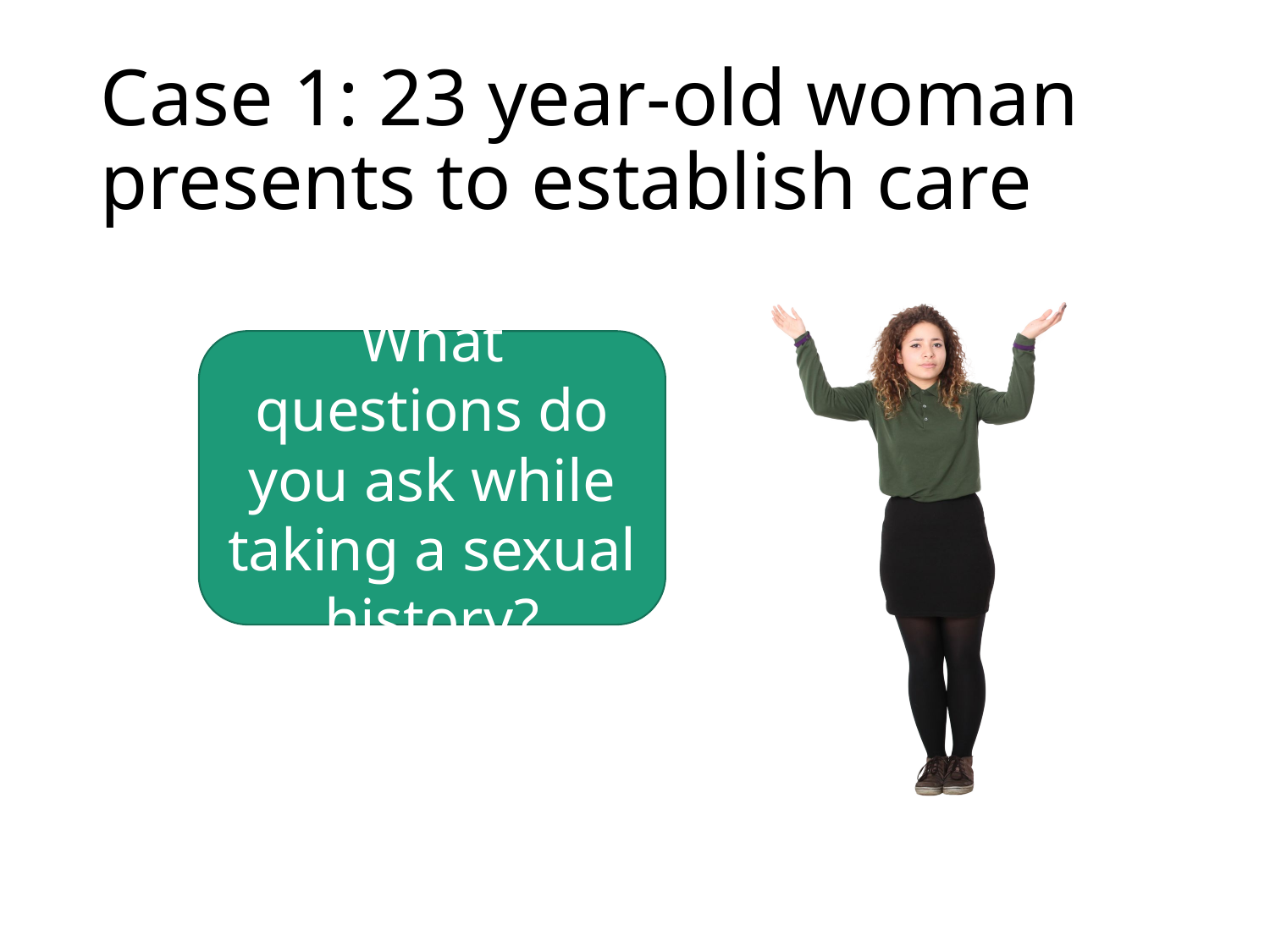

# Case 1: 23 year-old woman presents to establish care
What questions do you ask while taking a sexual history?

## Slide 12
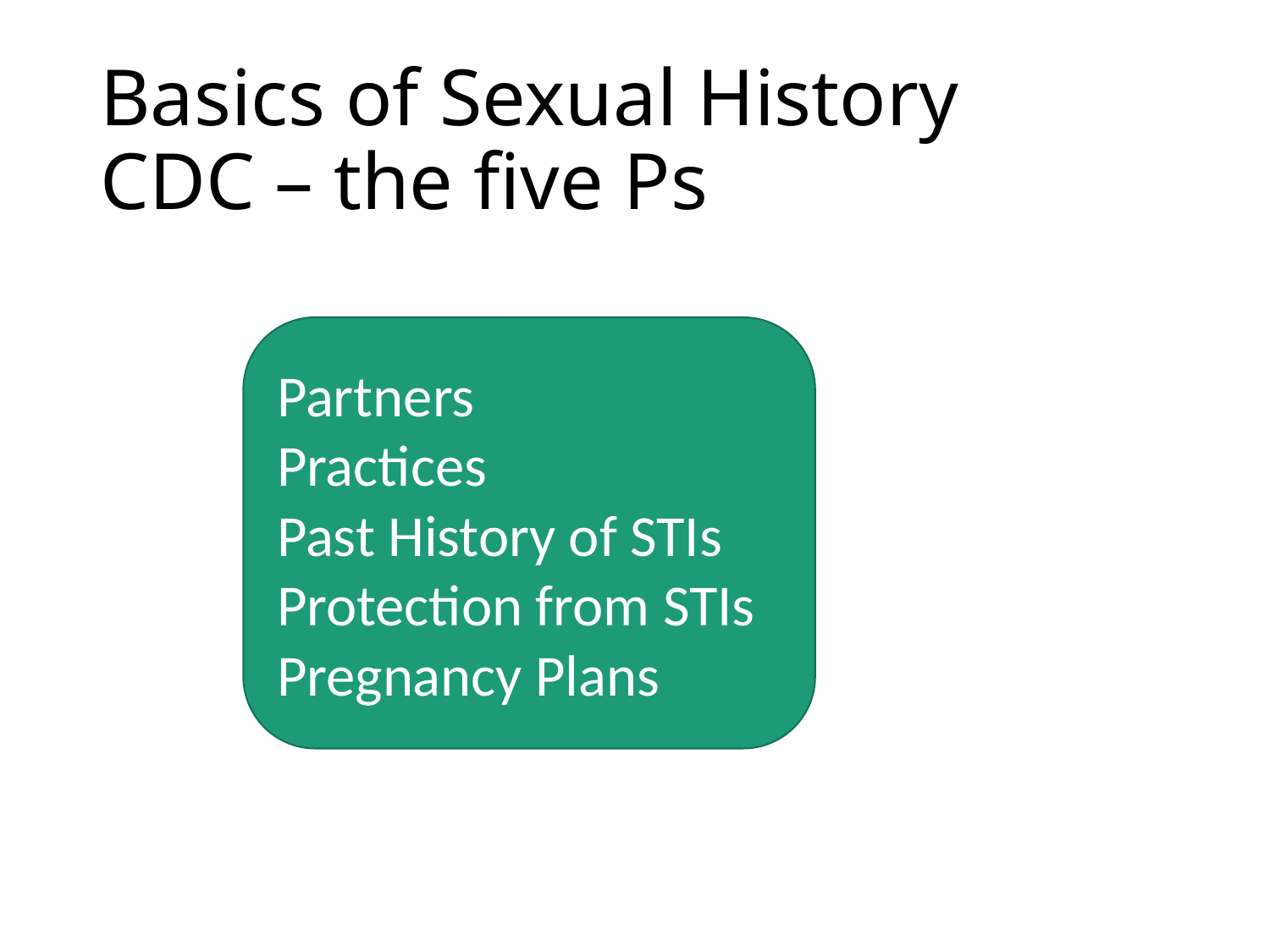

# Basics of Sexual History CDC – the five Ps
Partners
Practices
Past History of STIs
Protection from STIs
Pregnancy Plans

## Slide 13
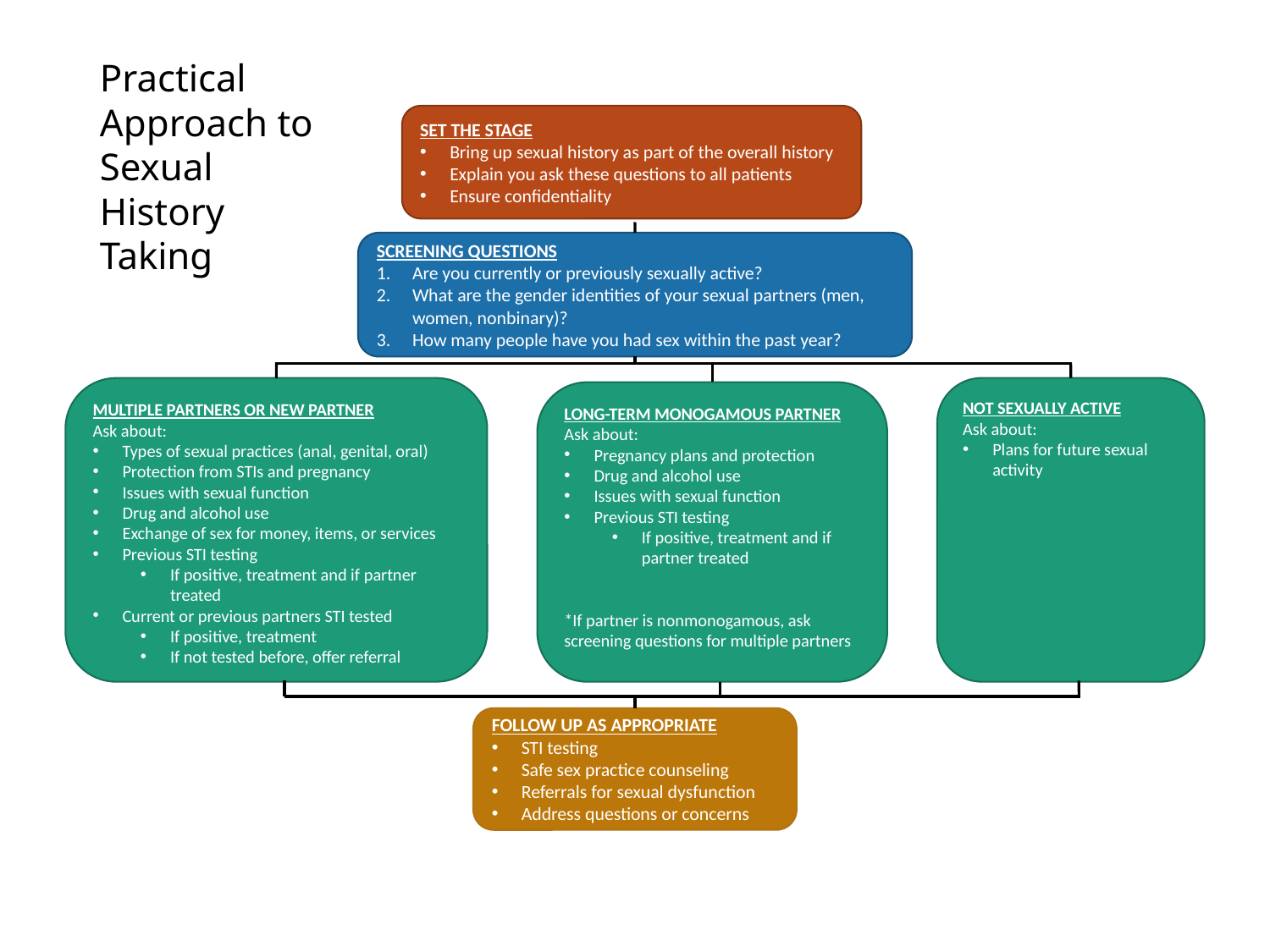

Practical Approach to Sexual History Taking
SET THE STAGE
Bring up sexual history as part of the overall history
Explain you ask these questions to all patients
Ensure confidentiality
SCREENING QUESTIONS
Are you currently or previously sexually active?
What are the gender identities of your sexual partners (men, women, nonbinary)?
How many people have you had sex within the past year?
MULTIPLE PARTNERS OR NEW PARTNER
Ask about:
Types of sexual practices (anal, genital, oral)
Protection from STIs and pregnancy
Issues with sexual function
Drug and alcohol use
Exchange of sex for money, items, or services
Previous STI testing
If positive, treatment and if partner treated
Current or previous partners STI tested
If positive, treatment
If not tested before, offer referral
NOT SEXUALLY ACTIVE
Ask about:
Plans for future sexual activity
LONG-TERM MONOGAMOUS PARTNER
Ask about:
Pregnancy plans and protection
Drug and alcohol use
Issues with sexual function
Previous STI testing
If positive, treatment and if partner treated
*If partner is nonmonogamous, ask screening questions for multiple partners
FOLLOW UP AS APPROPRIATE
STI testing
Safe sex practice counseling
Referrals for sexual dysfunction
Address questions or concerns

## Slide 14
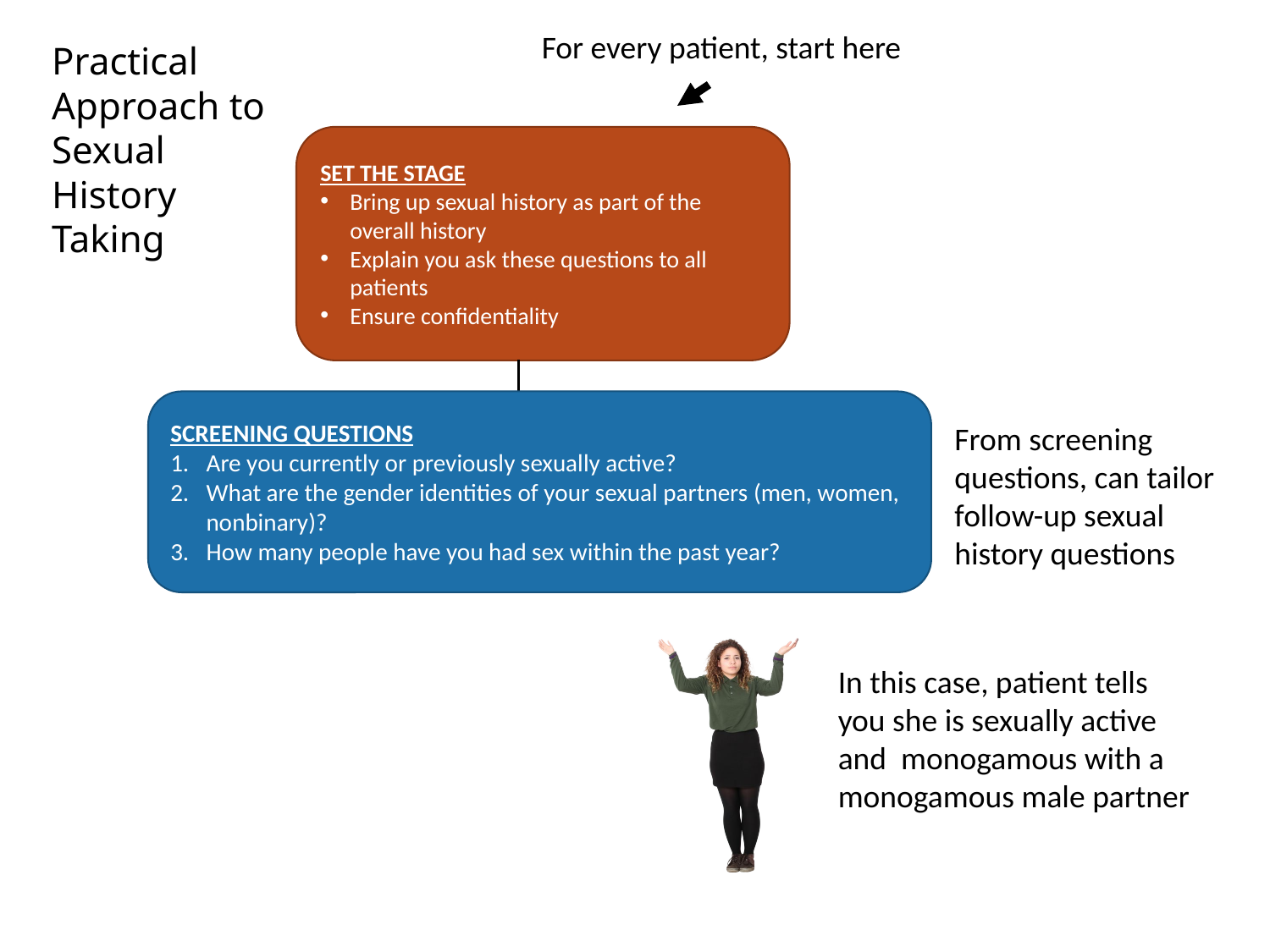

For every patient, start here
Practical Approach to Sexual History Taking
SET THE STAGE
Bring up sexual history as part of the overall history
Explain you ask these questions to all patients
Ensure confidentiality
SCREENING QUESTIONS
Are you currently or previously sexually active?
What are the gender identities of your sexual partners (men, women, nonbinary)?
How many people have you had sex within the past year?
From screening questions, can tailor follow-up sexual history questions
In this case, patient tells you she is sexually active and monogamous with a monogamous male partner

## Slide 15
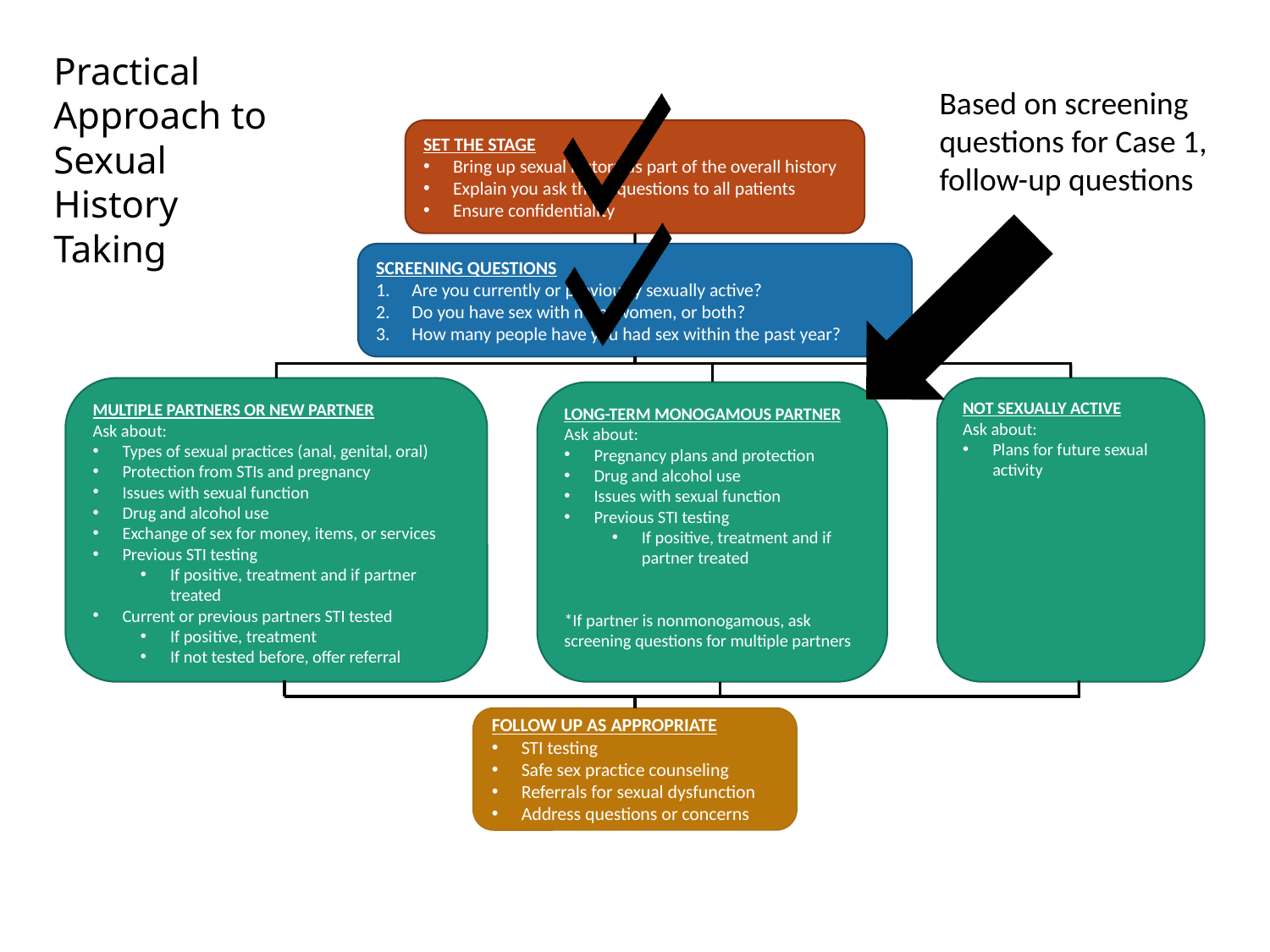

Practical Approach to Sexual History Taking
Based on screening questions for Case 1, follow-up questions
SET THE STAGE
Bring up sexual history as part of the overall history
Explain you ask these questions to all patients
Ensure confidentiality
SCREENING QUESTIONS
Are you currently or previously sexually active?
Do you have sex with men, women, or both?
How many people have you had sex within the past year?
MULTIPLE PARTNERS OR NEW PARTNER
Ask about:
Types of sexual practices (anal, genital, oral)
Protection from STIs and pregnancy
Issues with sexual function
Drug and alcohol use
Exchange of sex for money, items, or services
Previous STI testing
If positive, treatment and if partner treated
Current or previous partners STI tested
If positive, treatment
If not tested before, offer referral
NOT SEXUALLY ACTIVE
Ask about:
Plans for future sexual activity
LONG-TERM MONOGAMOUS PARTNER
Ask about:
Pregnancy plans and protection
Drug and alcohol use
Issues with sexual function
Previous STI testing
If positive, treatment and if partner treated
*If partner is nonmonogamous, ask screening questions for multiple partners
FOLLOW UP AS APPROPRIATE
STI testing
Safe sex practice counseling
Referrals for sexual dysfunction
Address questions or concerns

## Slide 16
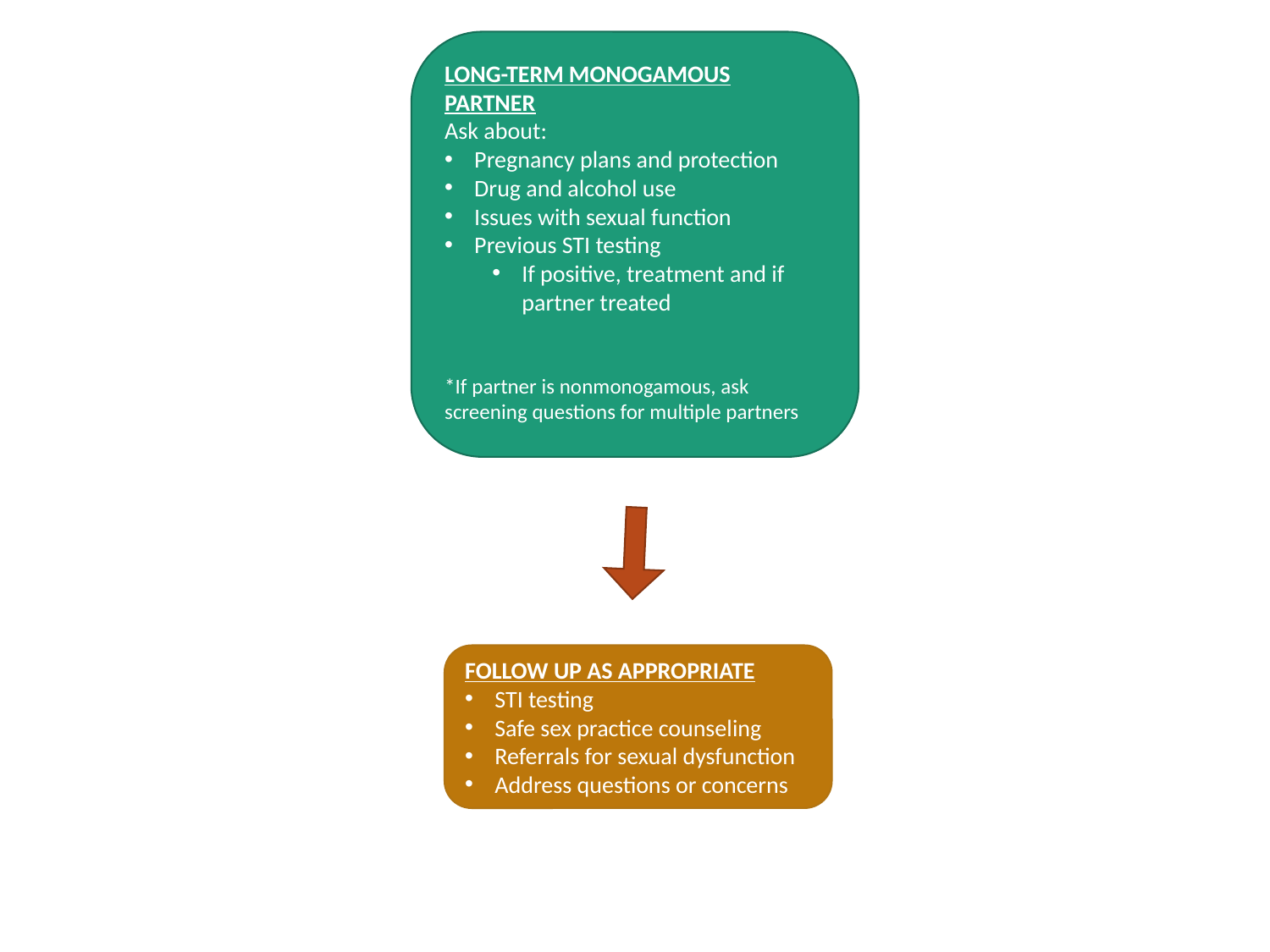

LONG-TERM MONOGAMOUS PARTNER
Ask about:
Pregnancy plans and protection
Drug and alcohol use
Issues with sexual function
Previous STI testing
If positive, treatment and if partner treated
*If partner is nonmonogamous, ask screening questions for multiple partners
FOLLOW UP AS APPROPRIATE
STI testing
Safe sex practice counseling
Referrals for sexual dysfunction
Address questions or concerns

## Slide 17
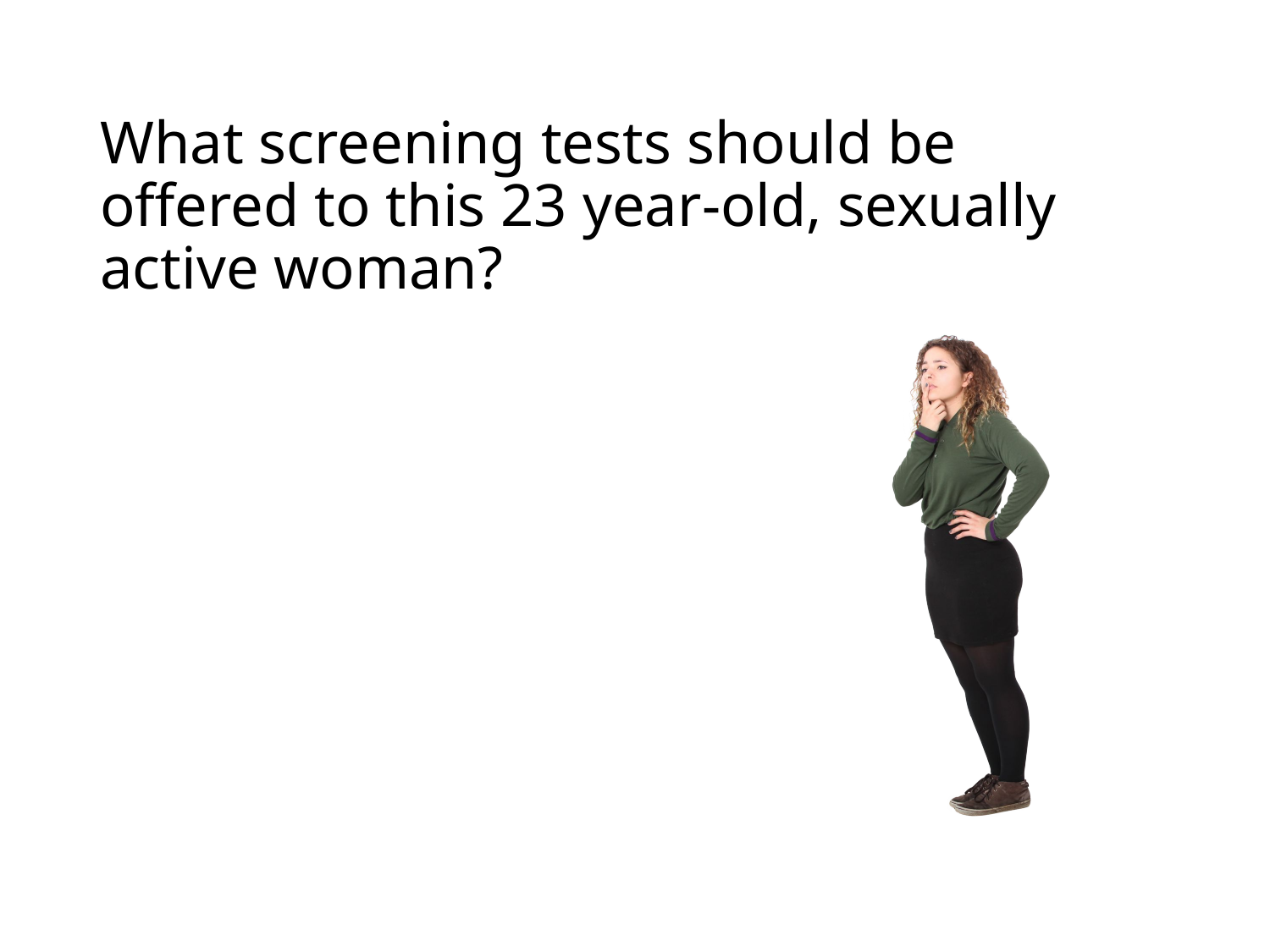

What screening tests should be offered to this 23 year-old, sexually active woman?

## Slide 18
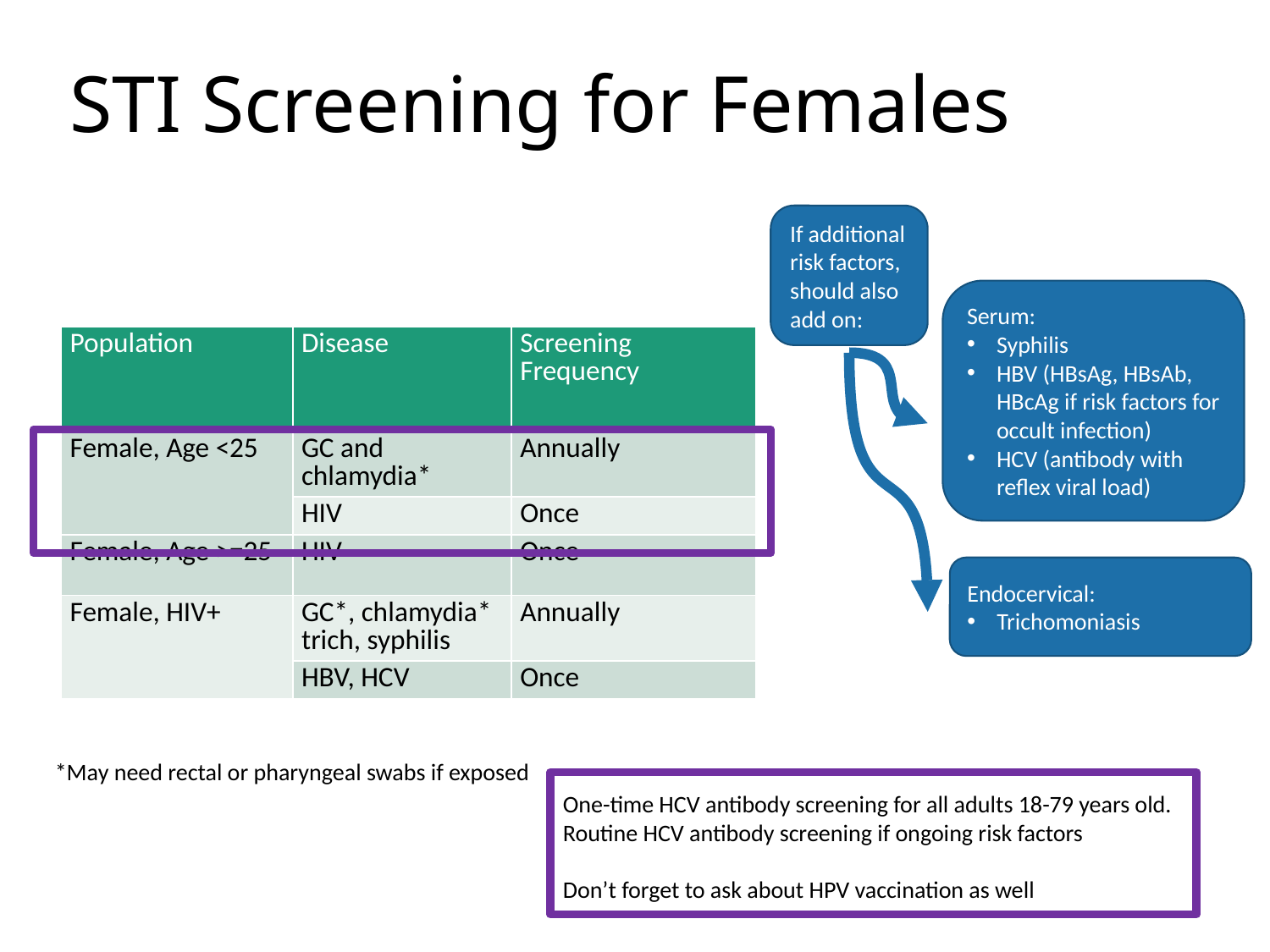

# STI Screening for Females
If additional risk factors, should also add on:
Serum:
Syphilis
HBV (HBsAg, HBsAb, HBcAg if risk factors for occult infection)
HCV (antibody with reflex viral load)
| Population | Disease | Screening Frequency |
| --- | --- | --- |
| Female, Age <25 | GC and chlamydia\* | Annually |
| | HIV | Once |
| Female, Age >=25 | HIV | Once |
| Female, HIV+ | GC\*, chlamydia\* trich, syphilis | Annually |
| | HBV, HCV | Once |
Endocervical:
Trichomoniasis
*May need rectal or pharyngeal swabs if exposed
One-time HCV antibody screening for all adults 18-79 years old.
Routine HCV antibody screening if ongoing risk factors
Don’t forget to ask about HPV vaccination as well

## Slide 19
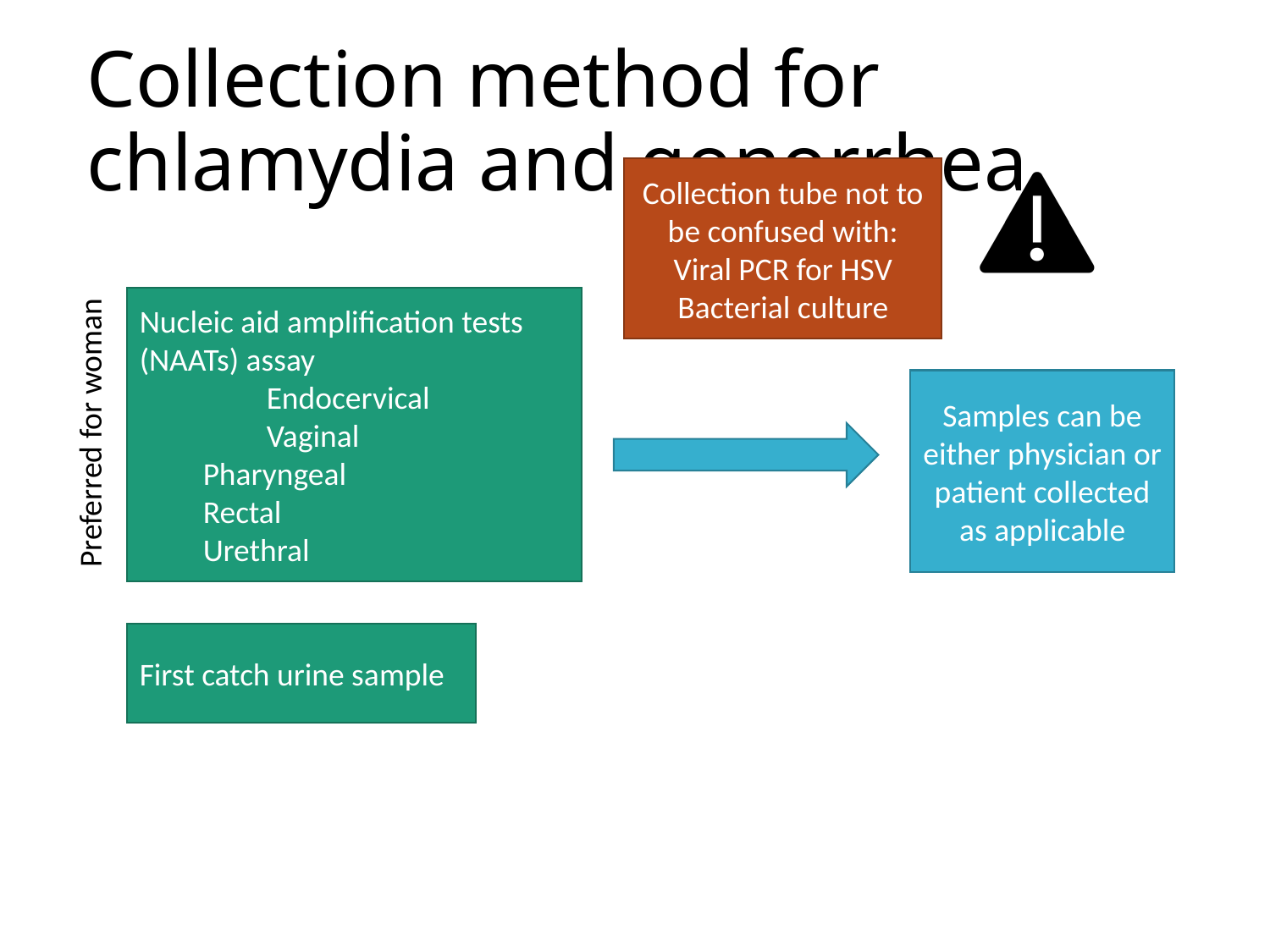

# Collection method for chlamydia and gonorrhea
Collection tube not to be confused with:
Viral PCR for HSV
Bacterial culture
Preferred for woman
Nucleic aid amplification tests (NAATs) assay
	Endocervical
	Vaginal
Pharyngeal
Rectal
Urethral
Samples can be either physician or patient collected as applicable
First catch urine sample

## Slide 20
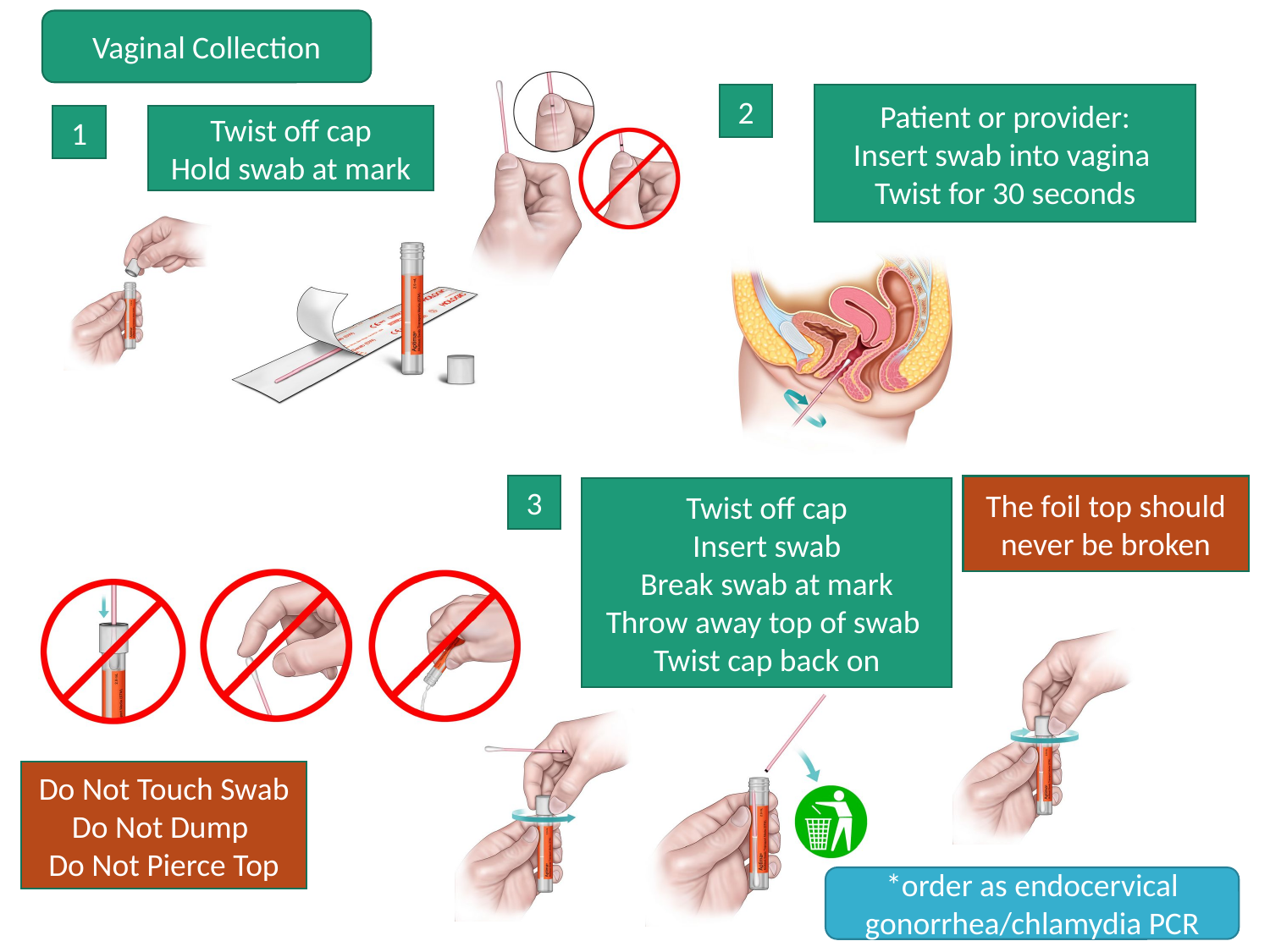

Vaginal Collection
2
Patient or provider:
Insert swab into vagina
Twist for 30 seconds
1
Twist off cap
Hold swab at mark
3
The foil top should never be broken
Twist off cap
Insert swab
Break swab at mark
Throw away top of swab
Twist cap back on
Do Not Touch Swab
Do Not Dump
Do Not Pierce Top
*order as endocervical gonorrhea/chlamydia PCR

## Slide 21
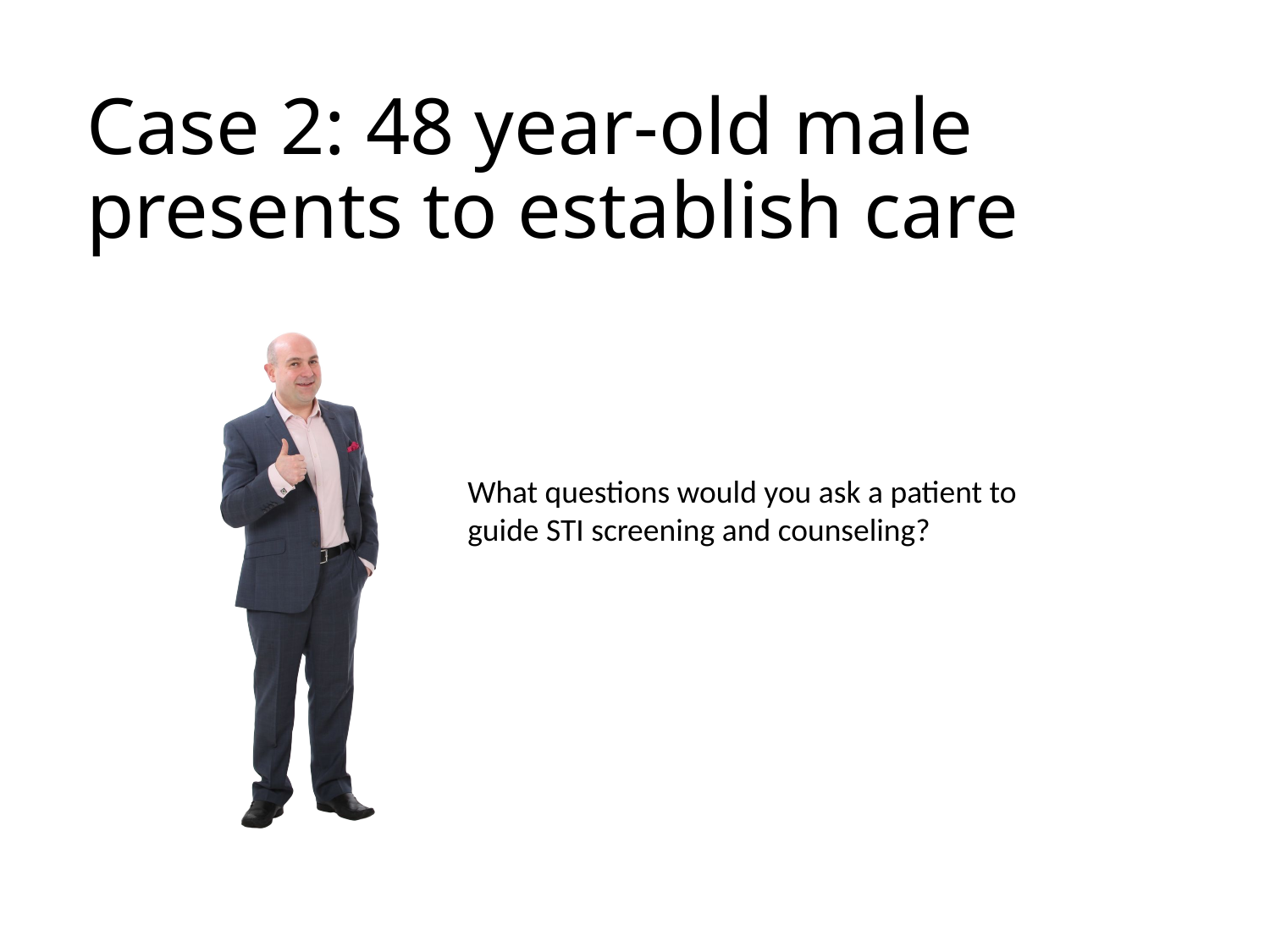

# Case 2: 48 year-old male presents to establish care
What questions would you ask a patient to guide STI screening and counseling?

## Slide 22
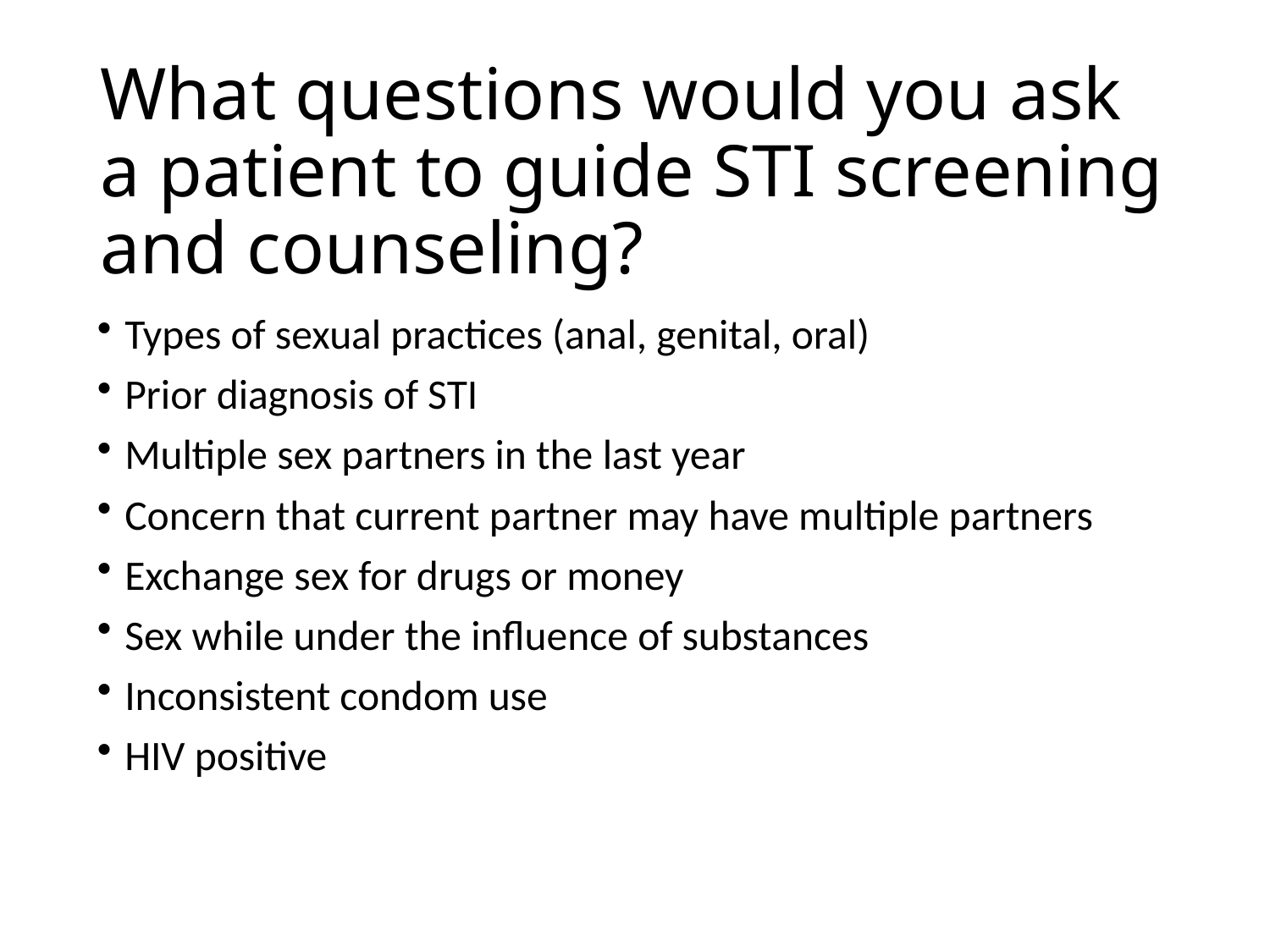

# What questions would you ask a patient to guide STI screening and counseling?
Types of sexual practices (anal, genital, oral)
Prior diagnosis of STI
Multiple sex partners in the last year
Concern that current partner may have multiple partners
Exchange sex for drugs or money
Sex while under the influence of substances
Inconsistent condom use
HIV positive

## Slide 23
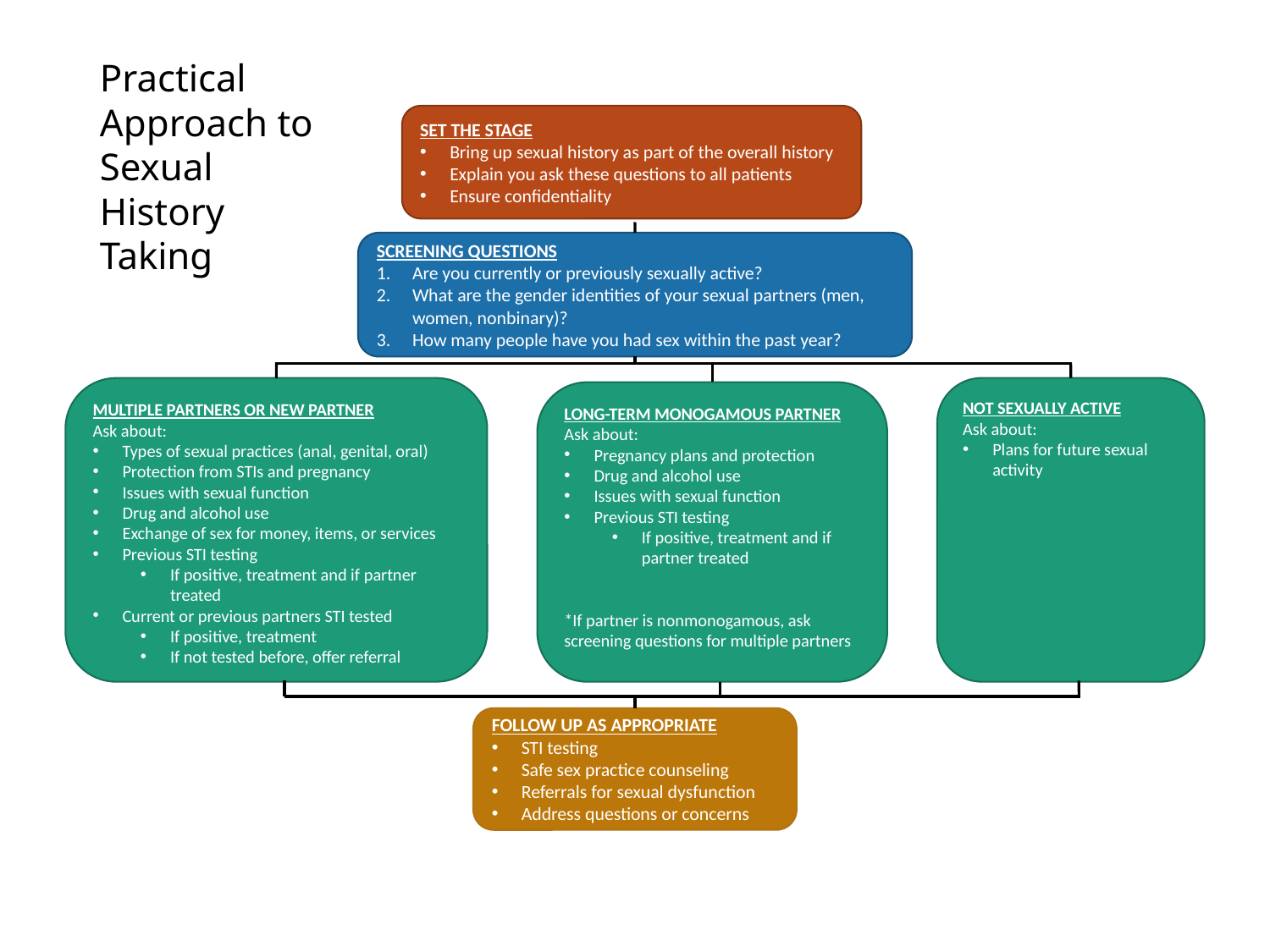

Practical Approach to Sexual History Taking
SET THE STAGE
Bring up sexual history as part of the overall history
Explain you ask these questions to all patients
Ensure confidentiality
SCREENING QUESTIONS
Are you currently or previously sexually active?
What are the gender identities of your sexual partners (men, women, nonbinary)?
How many people have you had sex within the past year?
MULTIPLE PARTNERS OR NEW PARTNER
Ask about:
Types of sexual practices (anal, genital, oral)
Protection from STIs and pregnancy
Issues with sexual function
Drug and alcohol use
Exchange of sex for money, items, or services
Previous STI testing
If positive, treatment and if partner treated
Current or previous partners STI tested
If positive, treatment
If not tested before, offer referral
NOT SEXUALLY ACTIVE
Ask about:
Plans for future sexual activity
LONG-TERM MONOGAMOUS PARTNER
Ask about:
Pregnancy plans and protection
Drug and alcohol use
Issues with sexual function
Previous STI testing
If positive, treatment and if partner treated
*If partner is nonmonogamous, ask screening questions for multiple partners
FOLLOW UP AS APPROPRIATE
STI testing
Safe sex practice counseling
Referrals for sexual dysfunction
Address questions or concerns

## Slide 24
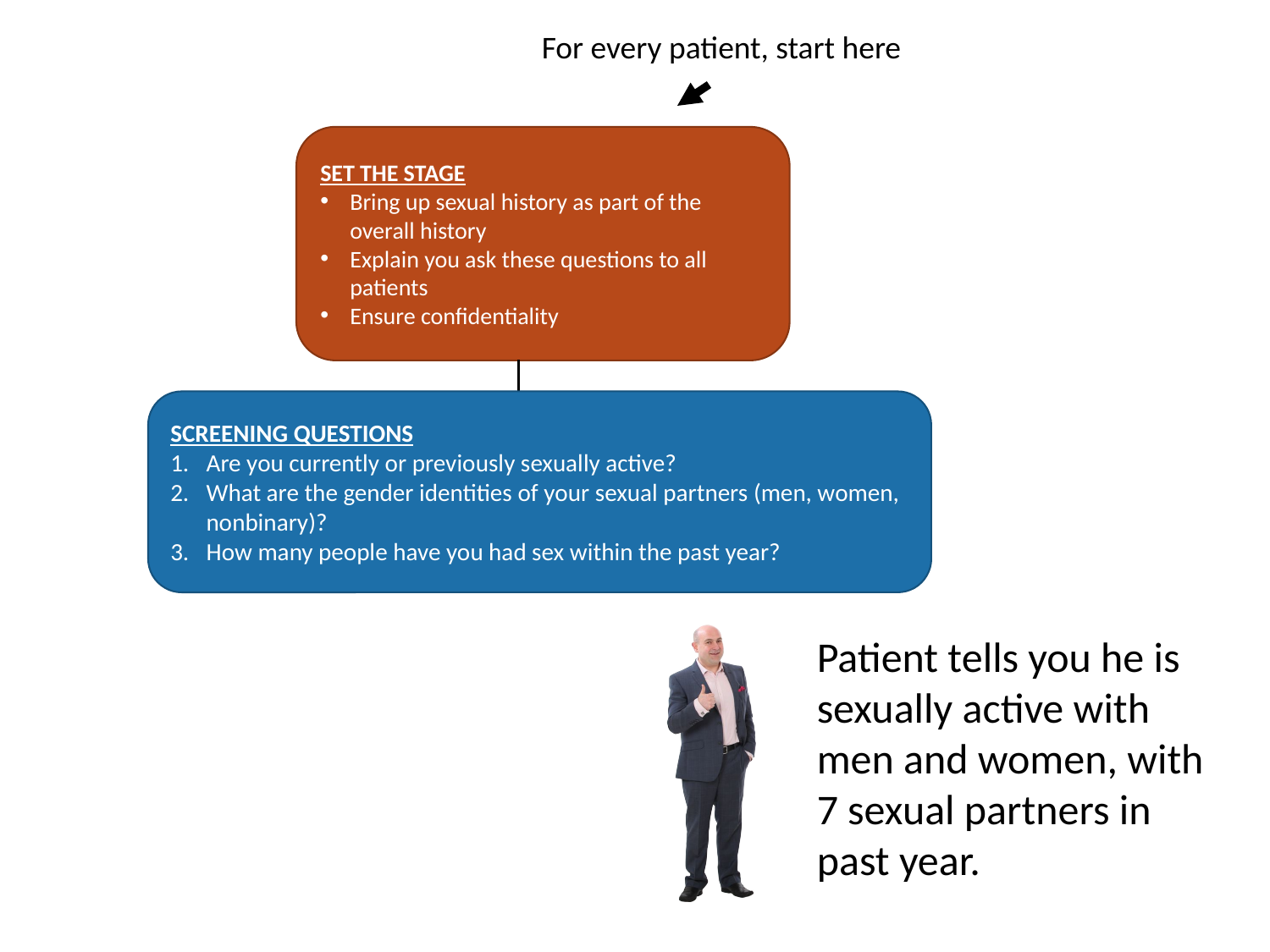

For every patient, start here
SET THE STAGE
Bring up sexual history as part of the overall history
Explain you ask these questions to all patients
Ensure confidentiality
SCREENING QUESTIONS
Are you currently or previously sexually active?
What are the gender identities of your sexual partners (men, women, nonbinary)?
How many people have you had sex within the past year?
Patient tells you he is sexually active with men and women, with 7 sexual partners in past year.

## Slide 25
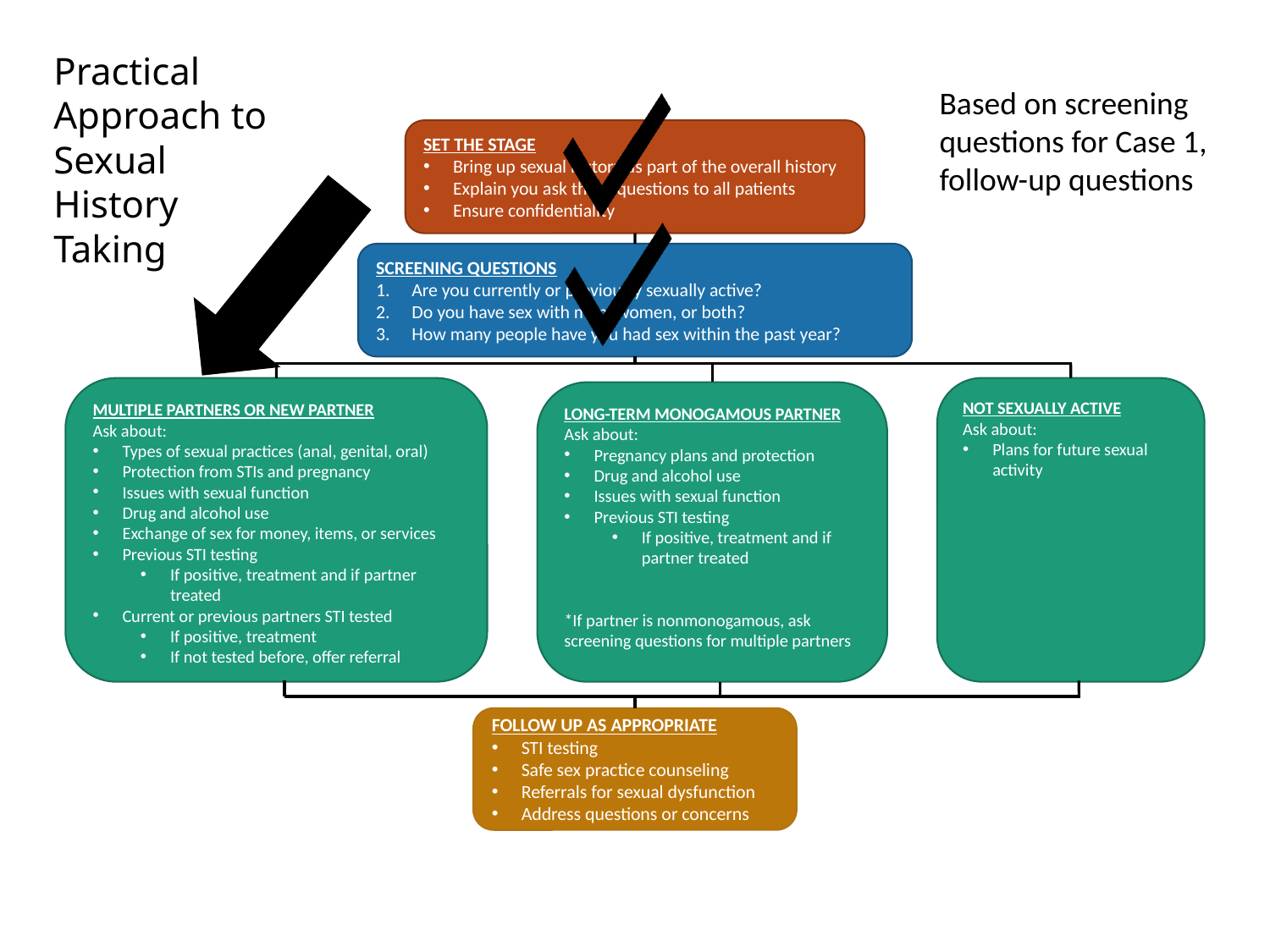

Practical Approach to Sexual History Taking
Based on screening questions for Case 1, follow-up questions
SET THE STAGE
Bring up sexual history as part of the overall history
Explain you ask these questions to all patients
Ensure confidentiality
SCREENING QUESTIONS
Are you currently or previously sexually active?
Do you have sex with men, women, or both?
How many people have you had sex within the past year?
MULTIPLE PARTNERS OR NEW PARTNER
Ask about:
Types of sexual practices (anal, genital, oral)
Protection from STIs and pregnancy
Issues with sexual function
Drug and alcohol use
Exchange of sex for money, items, or services
Previous STI testing
If positive, treatment and if partner treated
Current or previous partners STI tested
If positive, treatment
If not tested before, offer referral
NOT SEXUALLY ACTIVE
Ask about:
Plans for future sexual activity
LONG-TERM MONOGAMOUS PARTNER
Ask about:
Pregnancy plans and protection
Drug and alcohol use
Issues with sexual function
Previous STI testing
If positive, treatment and if partner treated
*If partner is nonmonogamous, ask screening questions for multiple partners
FOLLOW UP AS APPROPRIATE
STI testing
Safe sex practice counseling
Referrals for sexual dysfunction
Address questions or concerns

## Slide 26
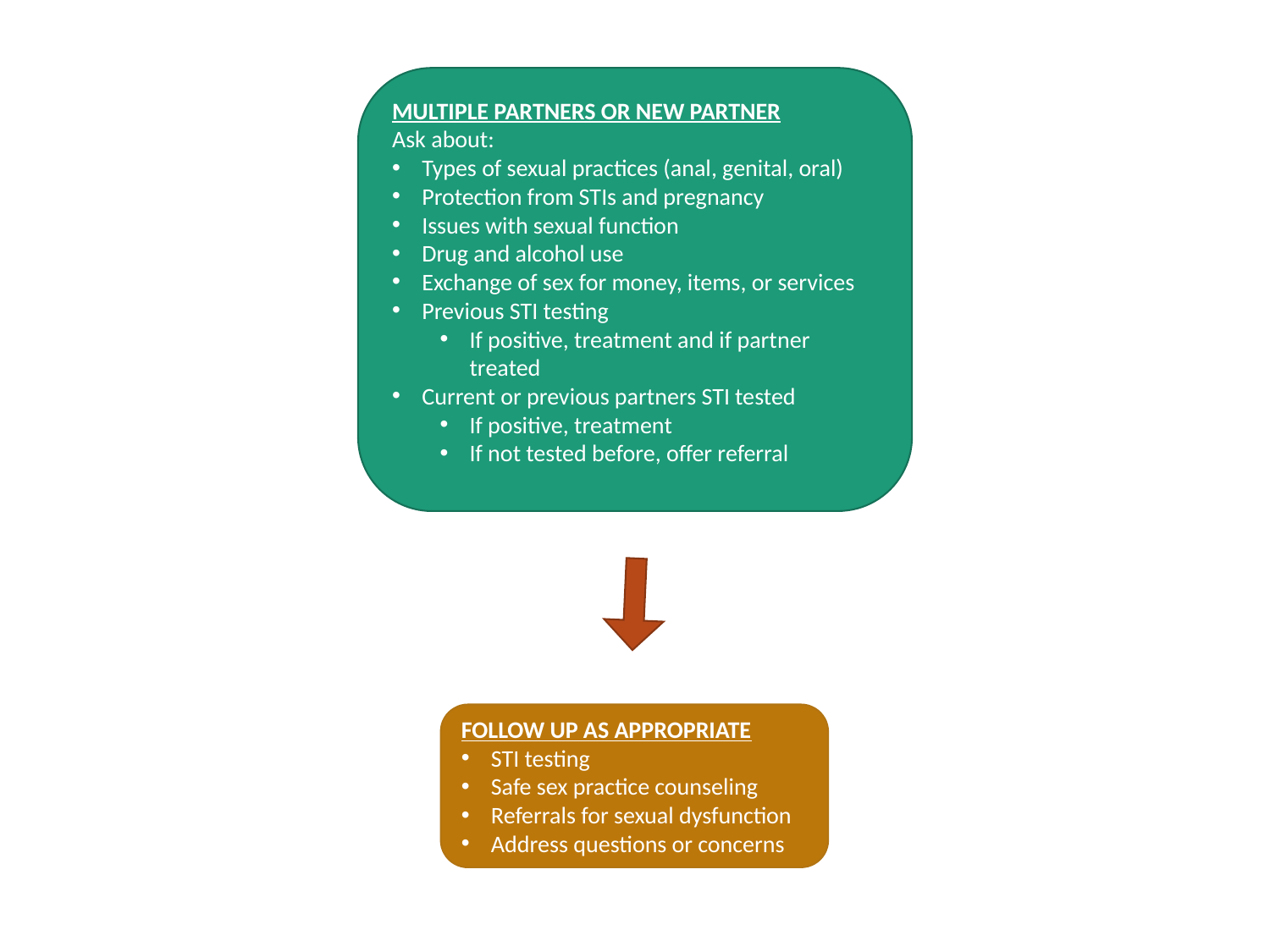

MULTIPLE PARTNERS OR NEW PARTNER
Ask about:
Types of sexual practices (anal, genital, oral)
Protection from STIs and pregnancy
Issues with sexual function
Drug and alcohol use
Exchange of sex for money, items, or services
Previous STI testing
If positive, treatment and if partner treated
Current or previous partners STI tested
If positive, treatment
If not tested before, offer referral
FOLLOW UP AS APPROPRIATE
STI testing
Safe sex practice counseling
Referrals for sexual dysfunction
Address questions or concerns

## Slide 27
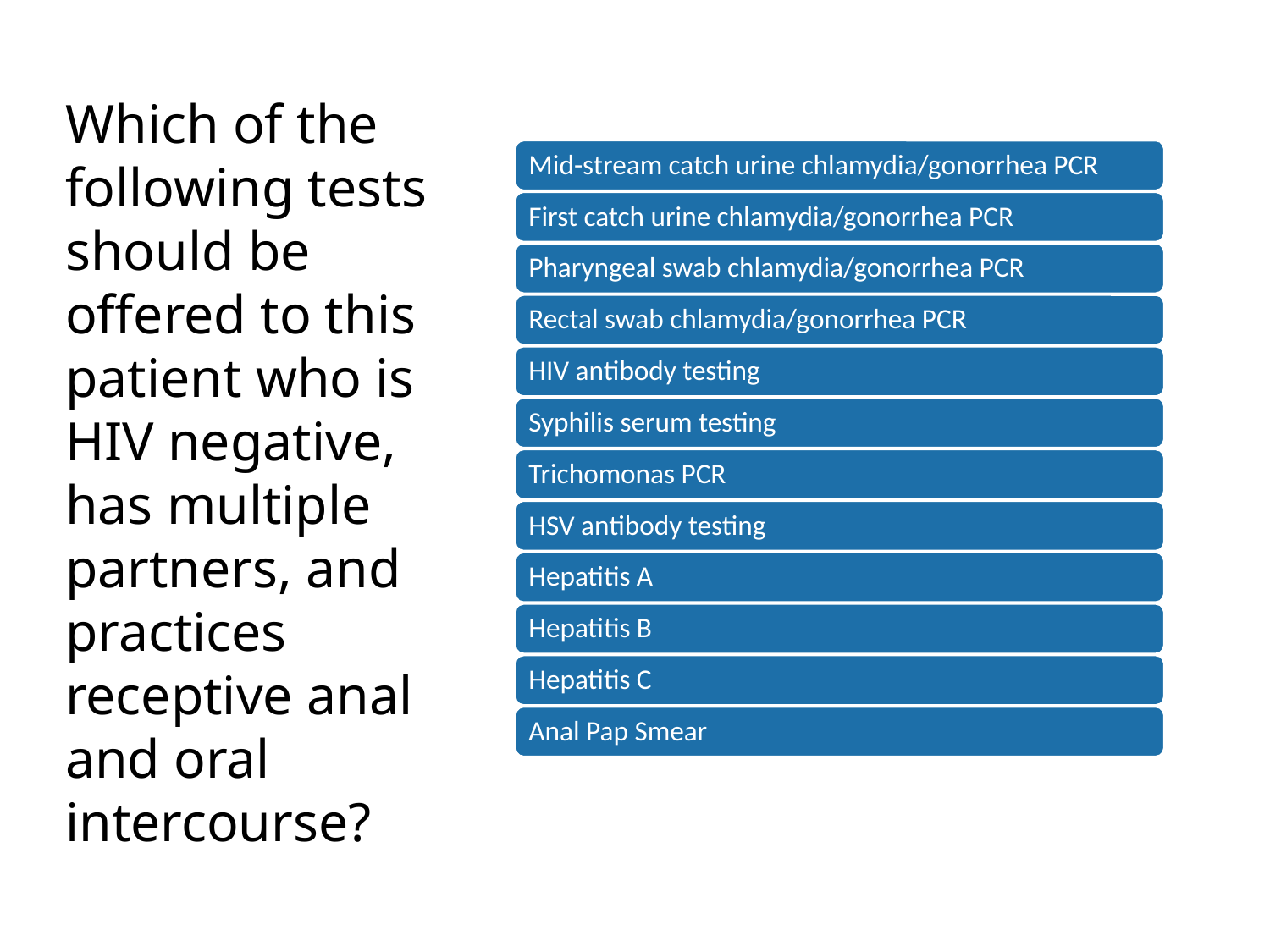

Which of the following tests should be offered to this patient who is HIV negative, has multiple partners, and practices receptive anal and oral intercourse?

## Slide 28
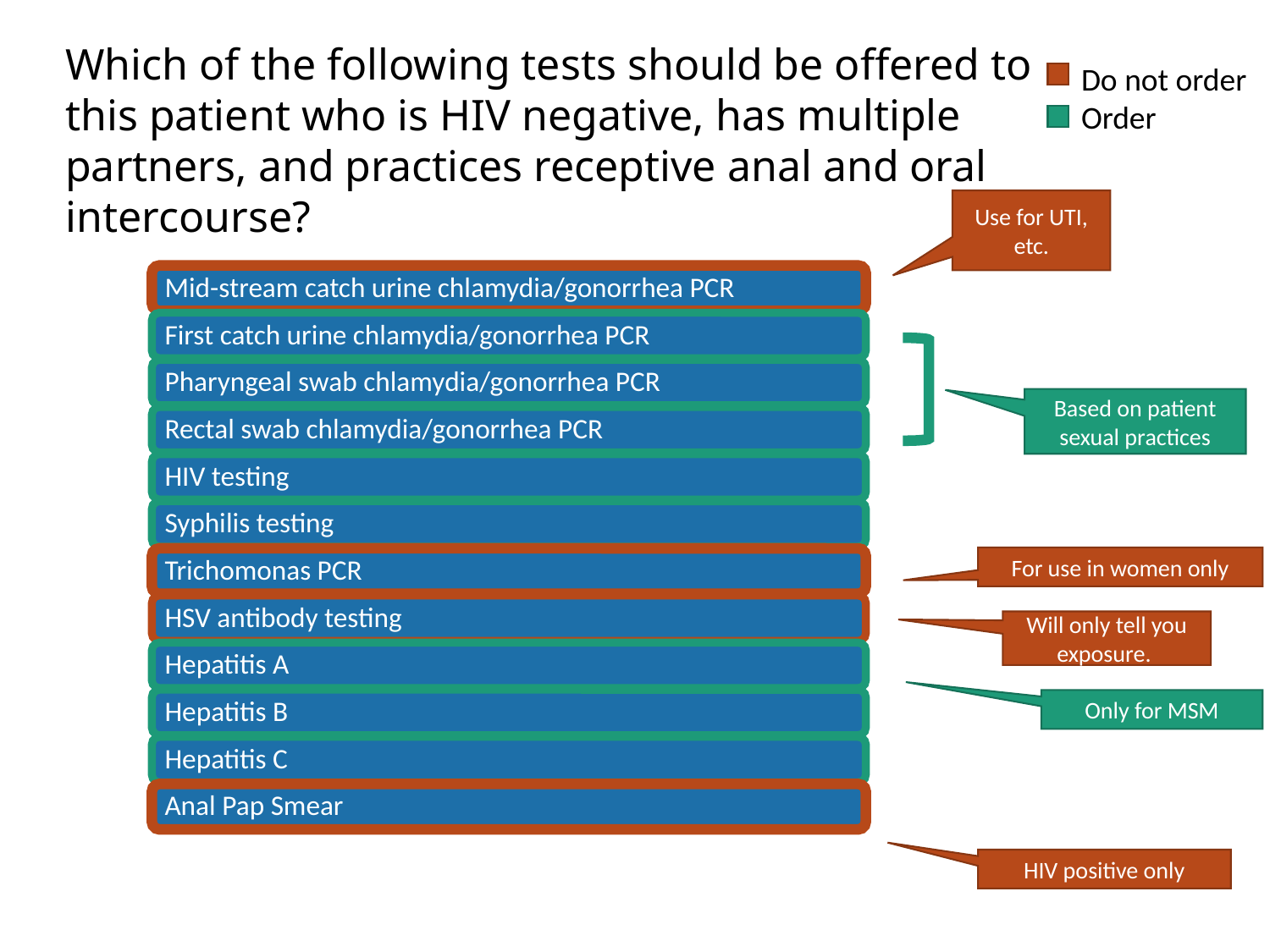

Which of the following tests should be offered to this patient who is HIV negative, has multiple partners, and practices receptive anal and oral intercourse?
Do not order
Order
Use for UTI, etc.
Based on patient sexual practices
For use in women only
Will only tell you exposure.
Only for MSM
HIV positive only

## Slide 29
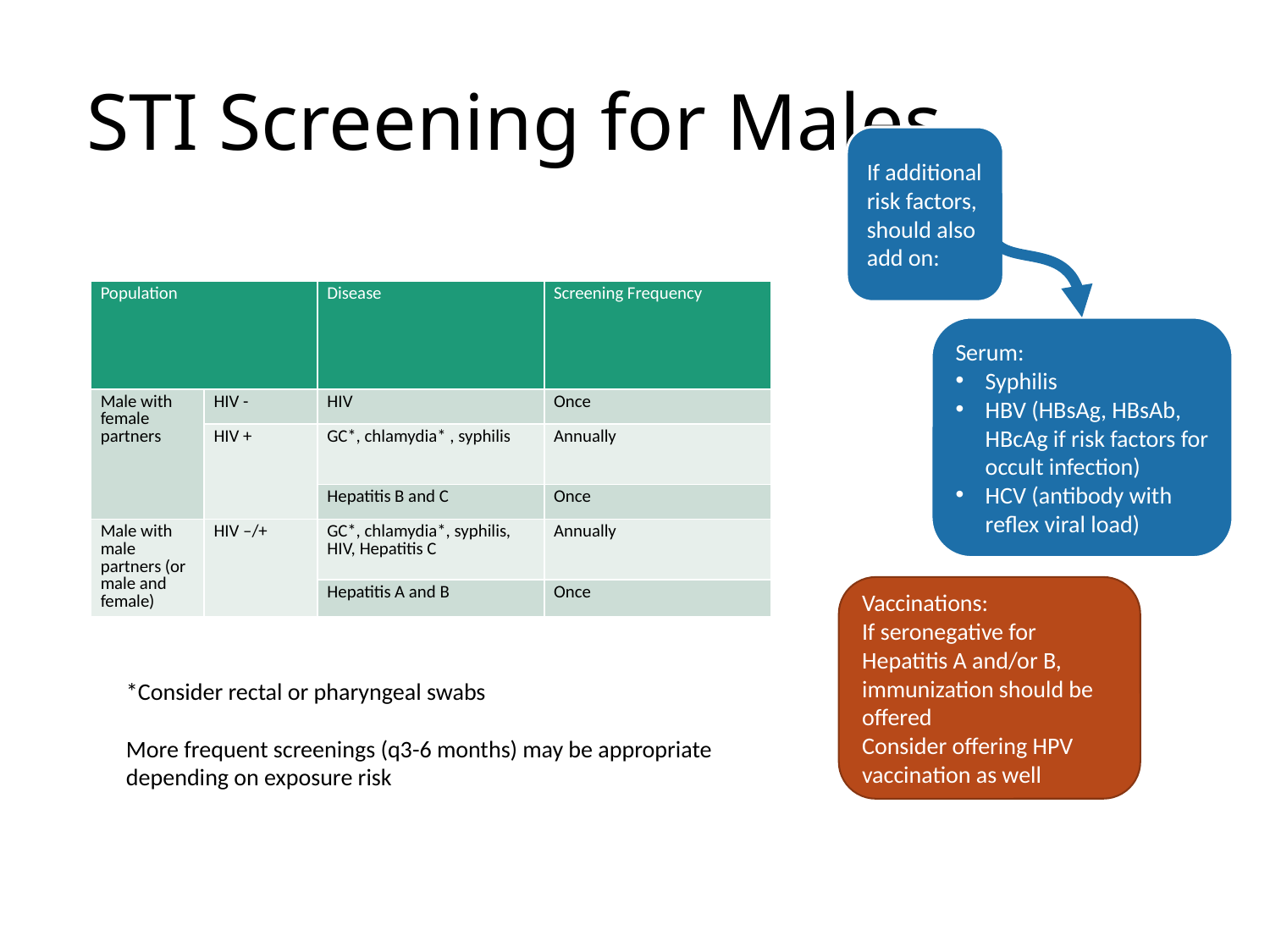

# STI Screening for Males
If additional risk factors,
should also add on:
| Population | | Disease | Screening Frequency |
| --- | --- | --- | --- |
| Male with female partners | HIV - | HIV | Once |
| | HIV + | GC\*, chlamydia\* , syphilis | Annually |
| | | Hepatitis B and C | Once |
| Male with male partners (or male and female) | HIV –/+ | GC\*, chlamydia\*, syphilis, HIV, Hepatitis C | Annually |
| | | Hepatitis A and B | Once |
Serum:
Syphilis
HBV (HBsAg, HBsAb, HBcAg if risk factors for occult infection)
HCV (antibody with reflex viral load)
Vaccinations:
If seronegative for Hepatitis A and/or B, immunization should be offered
Consider offering HPV vaccination as well
*Consider rectal or pharyngeal swabs
More frequent screenings (q3-6 months) may be appropriate depending on exposure risk

## Slide 30
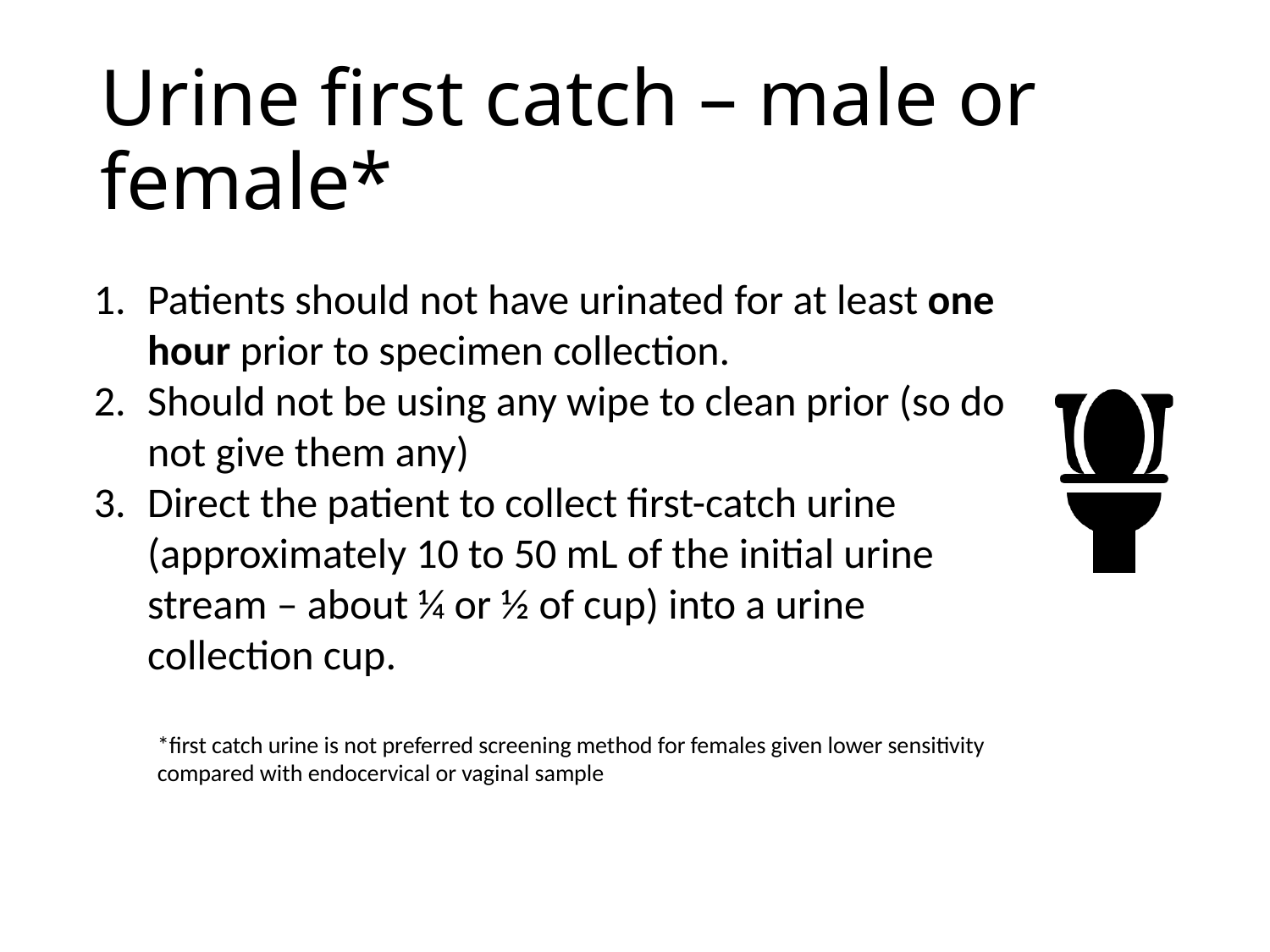

# Urine first catch – male or female*
Patients should not have urinated for at least one hour prior to specimen collection.
Should not be using any wipe to clean prior (so do not give them any)
Direct the patient to collect first-catch urine (approximately 10 to 50 mL of the initial urine stream – about ¼ or ½ of cup) into a urine collection cup.
*first catch urine is not preferred screening method for females given lower sensitivity compared with endocervical or vaginal sample

## Slide 31
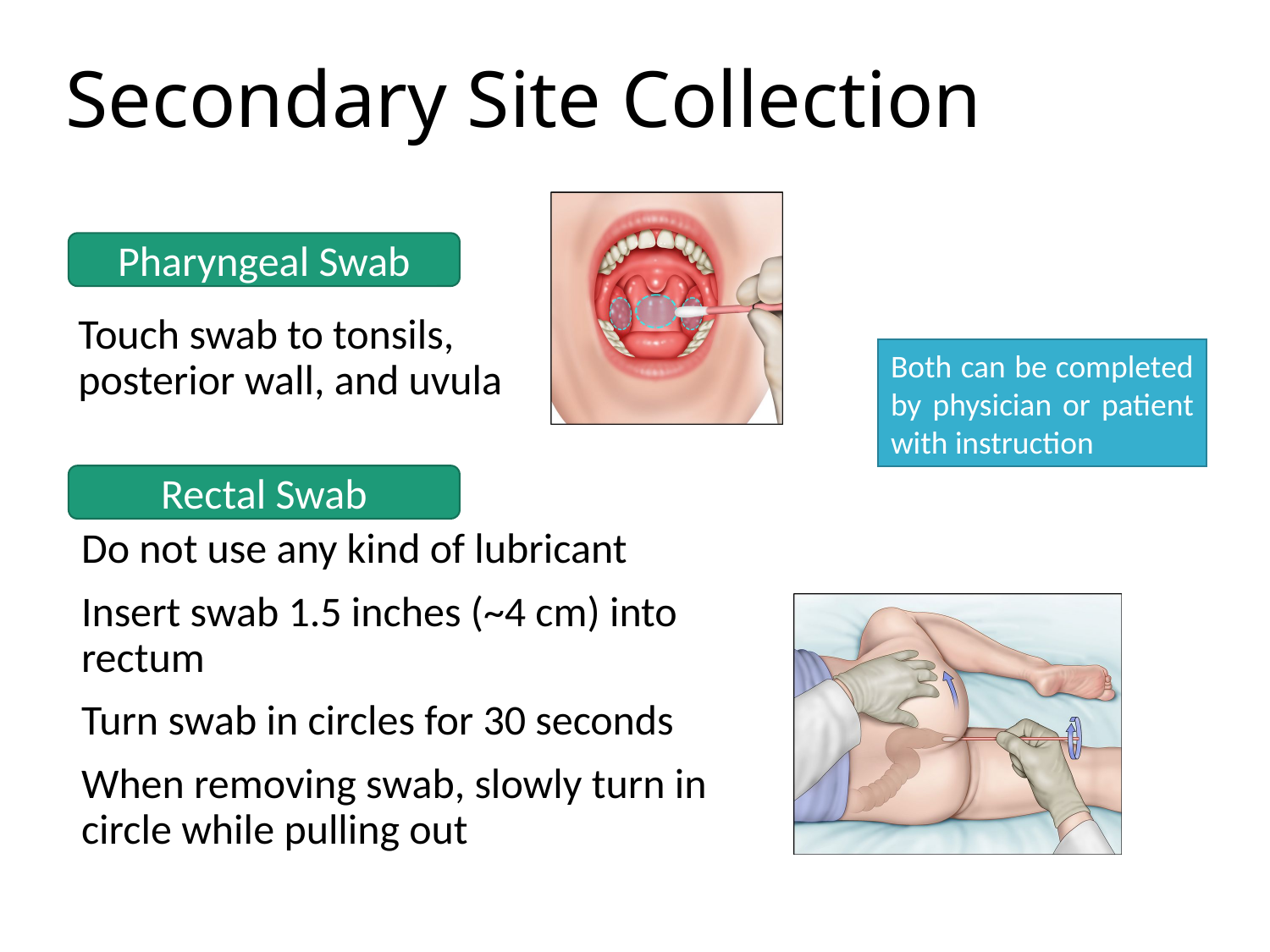

# Secondary Site Collection
Pharyngeal Swab
Touch swab to tonsils, posterior wall, and uvula
Both can be completed by physician or patient with instruction
Rectal Swab
Do not use any kind of lubricant
Insert swab 1.5 inches (~4 cm) into rectum
Turn swab in circles for 30 seconds
When removing swab, slowly turn in circle while pulling out

## Slide 32
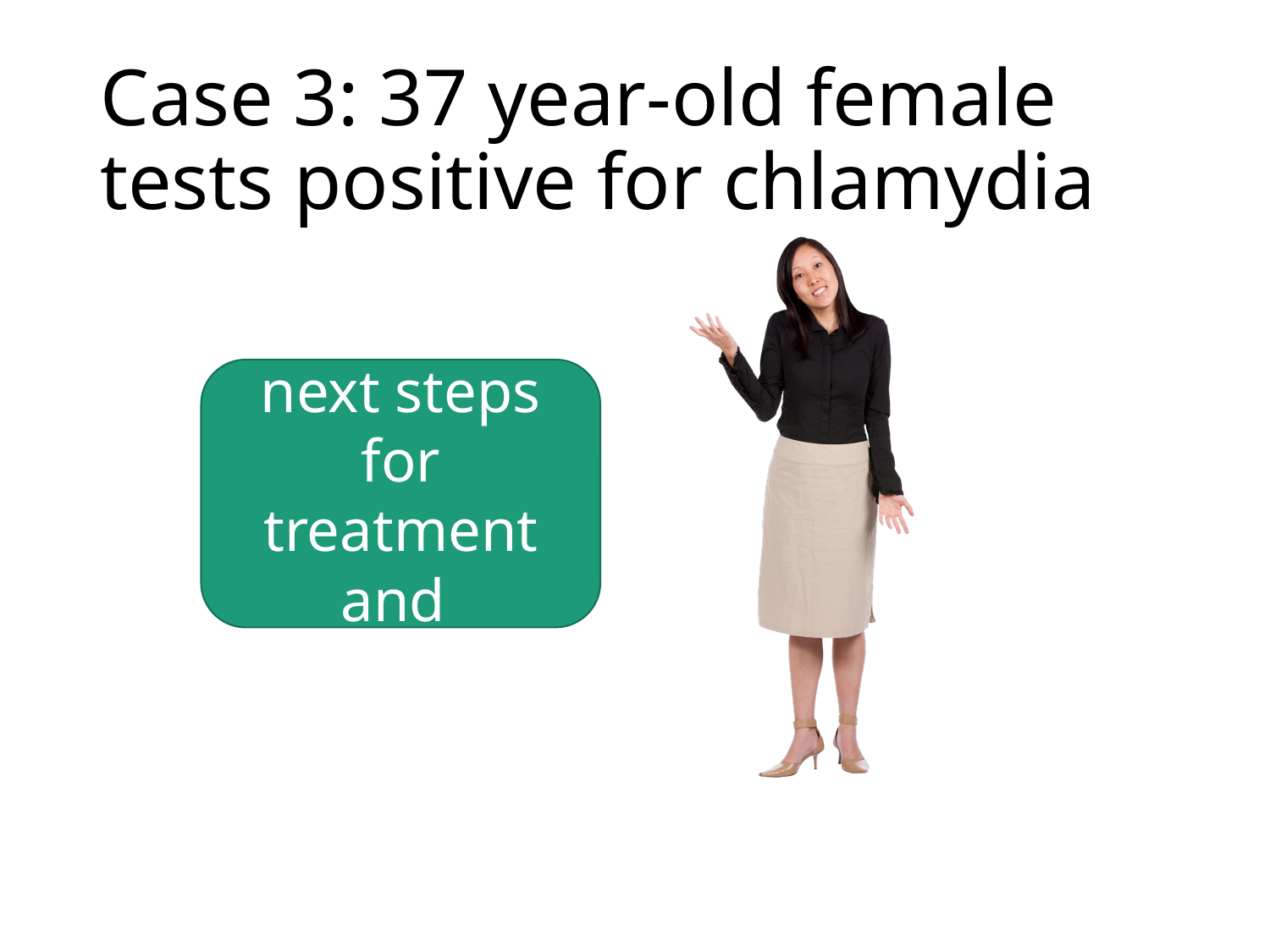

# Case 3: 37 year-old female tests positive for chlamydia
What are next steps for treatment and counseling ?

## Slide 33
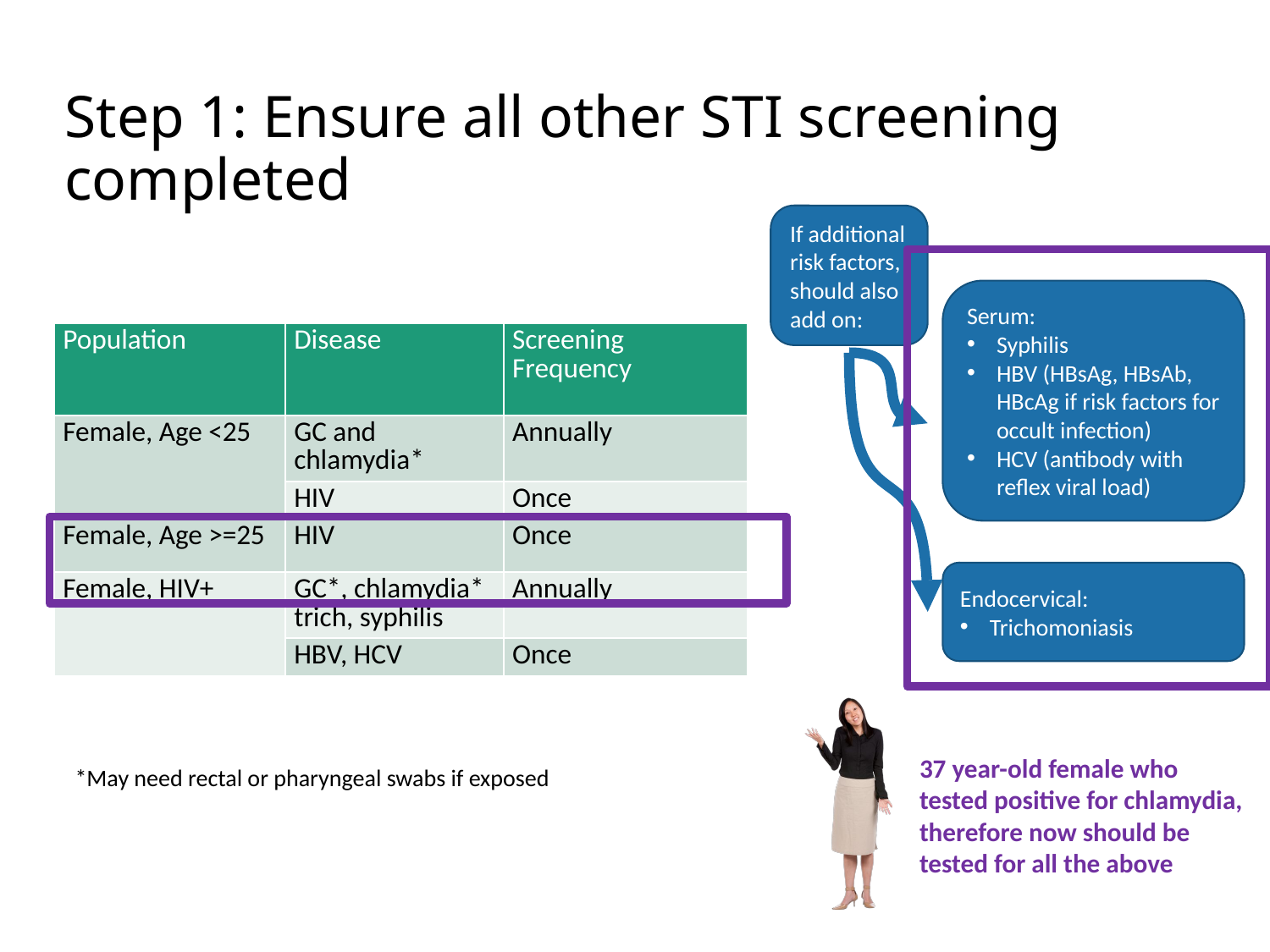

# Step 1: Ensure all other STI screening completed
If additional risk factors,
should also add on:
Serum:
Syphilis
HBV (HBsAg, HBsAb, HBcAg if risk factors for occult infection)
HCV (antibody with reflex viral load)
| Population | Disease | Screening Frequency |
| --- | --- | --- |
| Female, Age <25 | GC and chlamydia\* | Annually |
| | HIV | Once |
| Female, Age >=25 | HIV | Once |
| Female, HIV+ | GC\*, chlamydia\* trich, syphilis | Annually |
| | HBV, HCV | Once |
Endocervical:
Trichomoniasis
37 year-old female who tested positive for chlamydia, therefore now should be tested for all the above
*May need rectal or pharyngeal swabs if exposed

## Slide 34
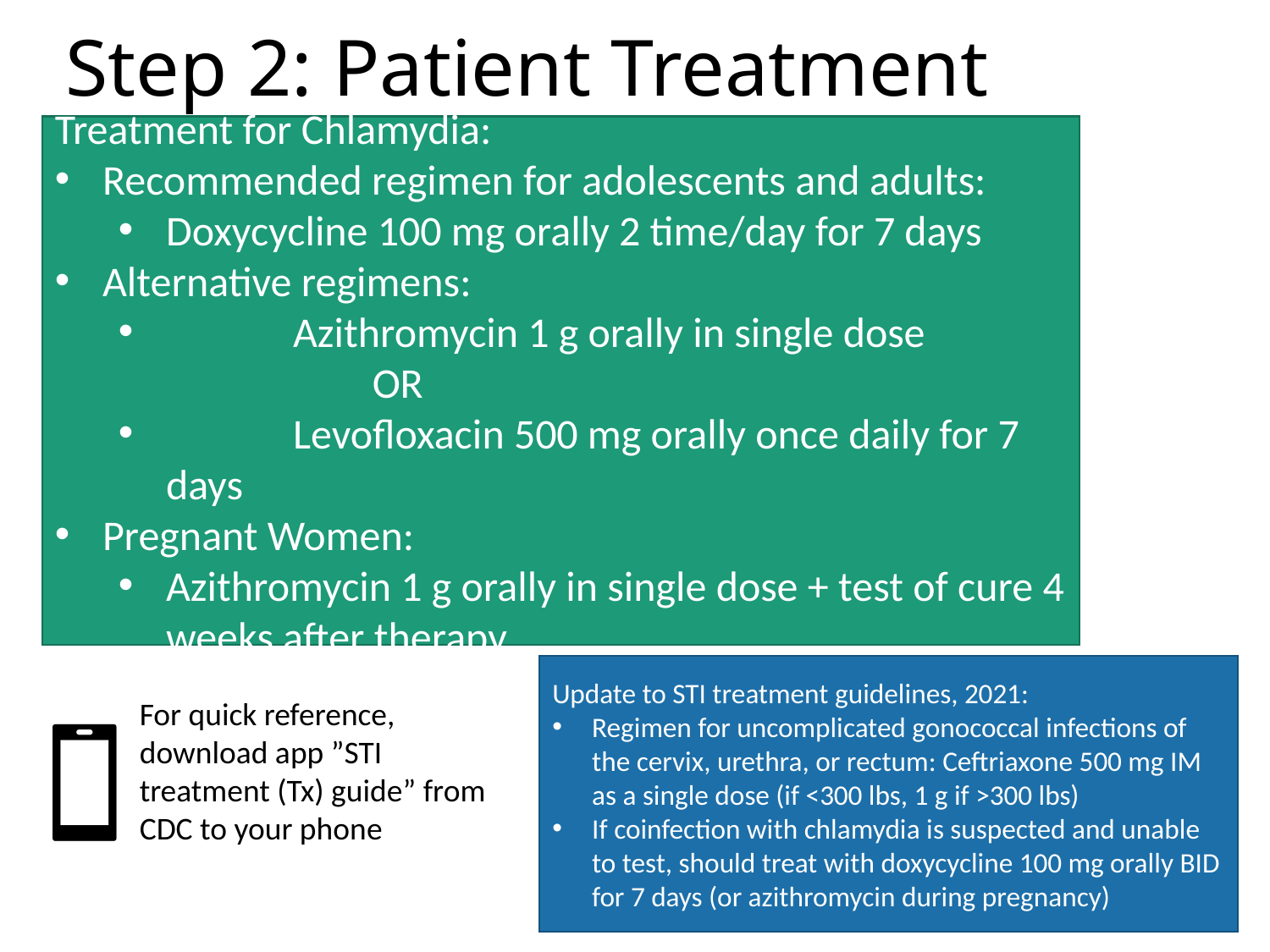

# Step 2: Patient Treatment
Treatment for Chlamydia:
Recommended regimen for adolescents and adults:
Doxycycline 100 mg orally 2 time/day for 7 days
Alternative regimens:
	Azithromycin 1 g orally in single dose
		OR
	Levofloxacin 500 mg orally once daily for 7 days
Pregnant Women:
Azithromycin 1 g orally in single dose + test of cure 4 weeks after therapy
Update to STI treatment guidelines, 2021:
Regimen for uncomplicated gonococcal infections of the cervix, urethra, or rectum: Ceftriaxone 500 mg IM as a single dose (if <300 lbs, 1 g if >300 lbs)
If coinfection with chlamydia is suspected and unable to test, should treat with doxycycline 100 mg orally BID for 7 days (or azithromycin during pregnancy)
For quick reference, download app ”STI treatment (Tx) guide” from CDC to your phone

## Slide 35
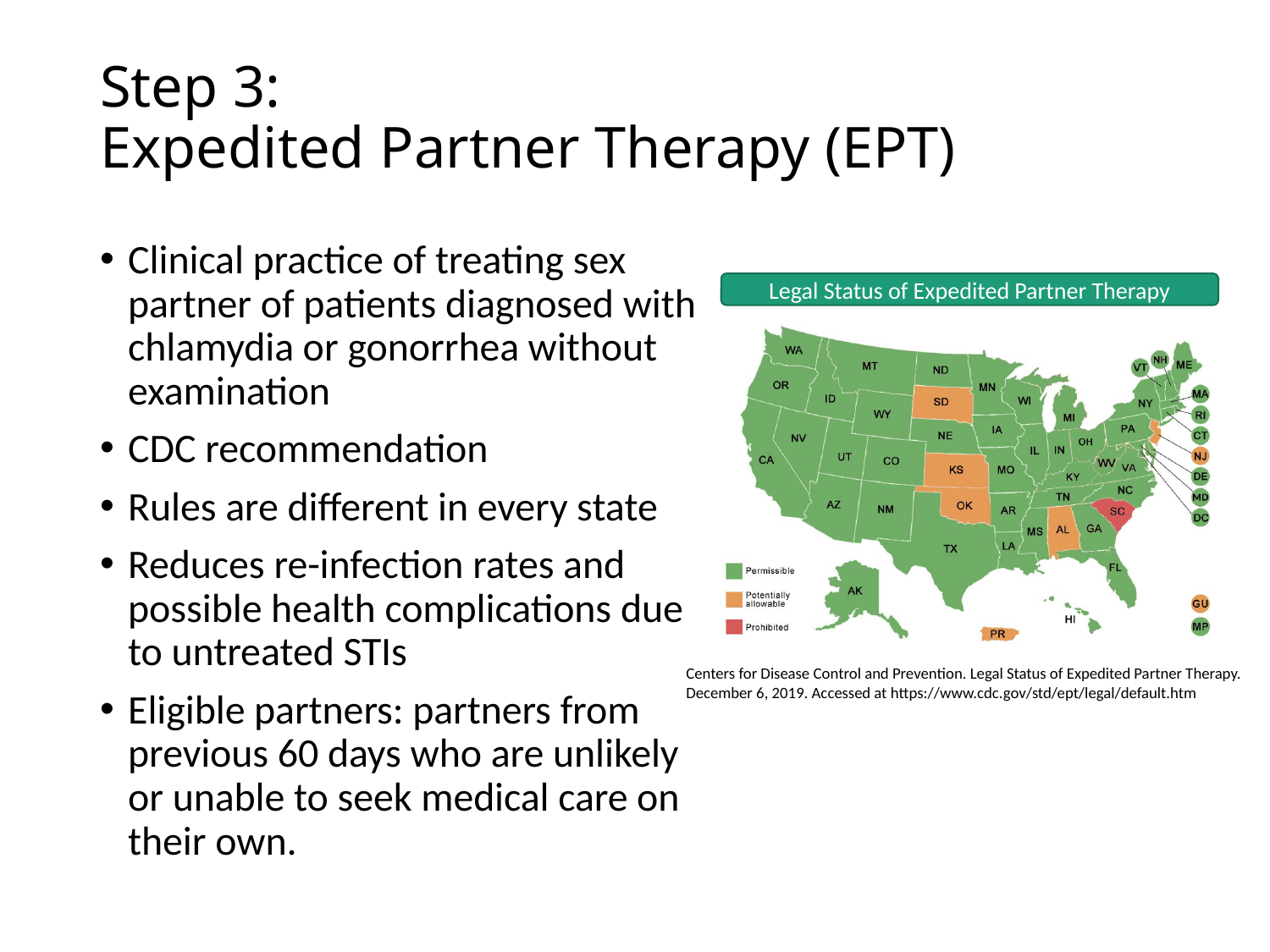

# Step 3: Expedited Partner Therapy (EPT)
Clinical practice of treating sex partner of patients diagnosed with chlamydia or gonorrhea without examination
CDC recommendation
Rules are different in every state
Reduces re-infection rates and possible health complications due to untreated STIs
Eligible partners: partners from previous 60 days who are unlikely or unable to seek medical care on their own.
Legal Status of Expedited Partner Therapy
Centers for Disease Control and Prevention. Legal Status of Expedited Partner Therapy. December 6, 2019. Accessed at https://www.cdc.gov/std/ept/legal/default.htm

## Slide 36
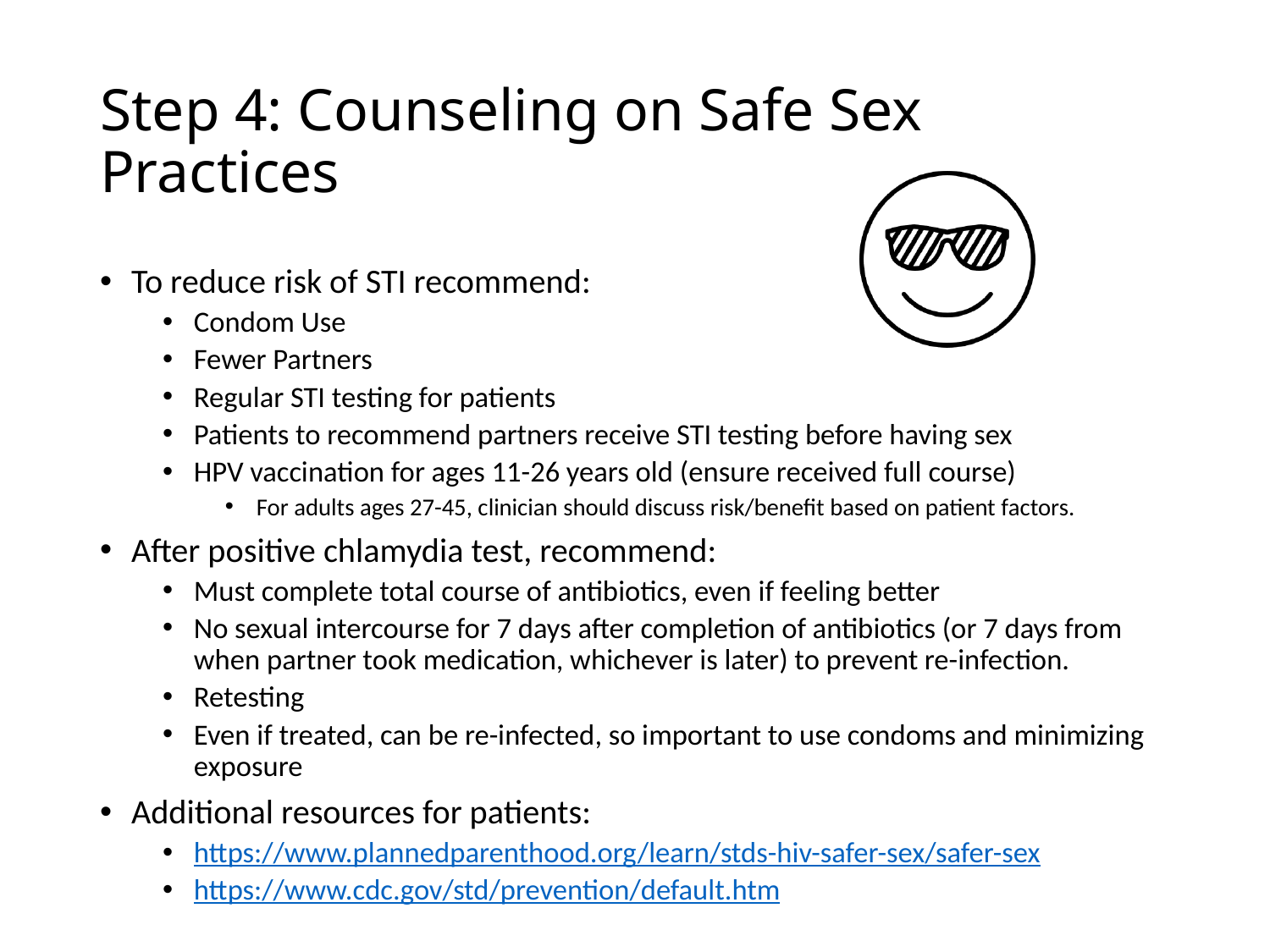

# Step 4: Counseling on Safe Sex Practices
To reduce risk of STI recommend:
Condom Use
Fewer Partners
Regular STI testing for patients
Patients to recommend partners receive STI testing before having sex
HPV vaccination for ages 11-26 years old (ensure received full course)
For adults ages 27-45, clinician should discuss risk/benefit based on patient factors.
After positive chlamydia test, recommend:
Must complete total course of antibiotics, even if feeling better
No sexual intercourse for 7 days after completion of antibiotics (or 7 days from when partner took medication, whichever is later) to prevent re-infection.
Retesting
Even if treated, can be re-infected, so important to use condoms and minimizing exposure
Additional resources for patients:
https://www.plannedparenthood.org/learn/stds-hiv-safer-sex/safer-sex
https://www.cdc.gov/std/prevention/default.htm

## Slide 37
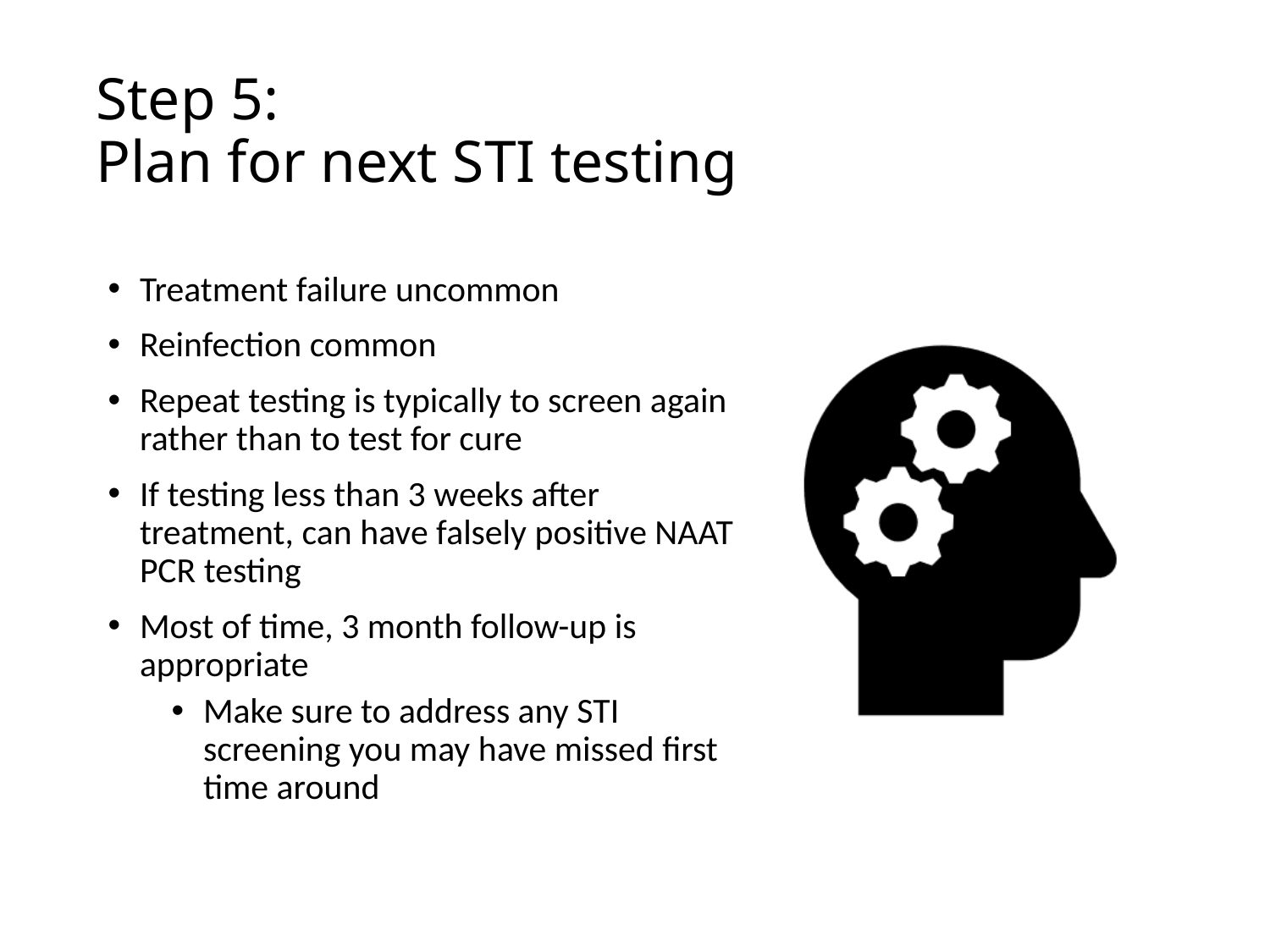

# Step 5: Plan for next STI testing
Treatment failure uncommon
Reinfection common
Repeat testing is typically to screen again rather than to test for cure
If testing less than 3 weeks after treatment, can have falsely positive NAAT PCR testing
Most of time, 3 month follow-up is appropriate
Make sure to address any STI screening you may have missed first time around

## Slide 38
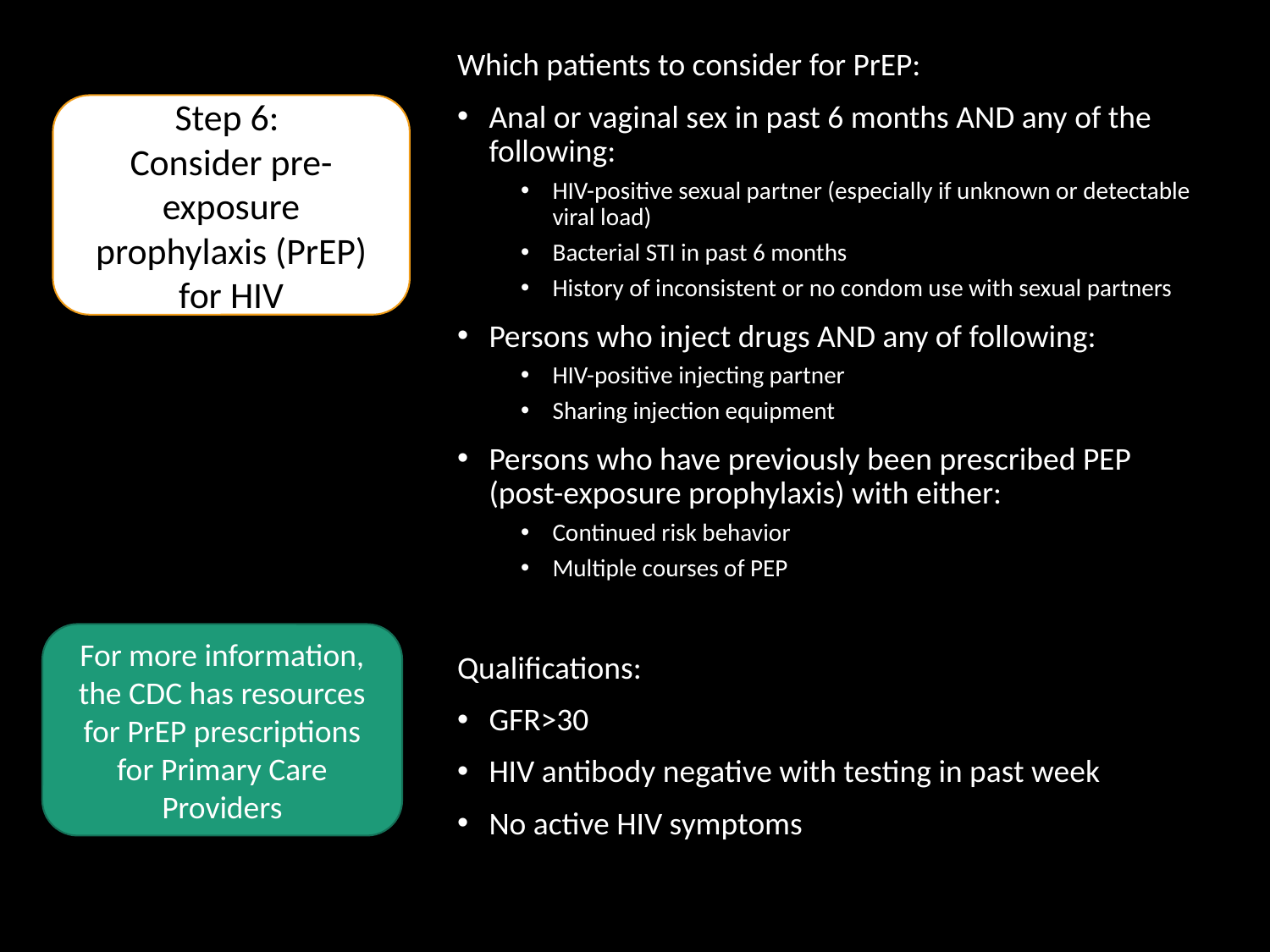

Which patients to consider for PrEP:
Anal or vaginal sex in past 6 months AND any of the following:
HIV-positive sexual partner (especially if unknown or detectable viral load)
Bacterial STI in past 6 months
History of inconsistent or no condom use with sexual partners
Persons who inject drugs AND any of following:
HIV-positive injecting partner
Sharing injection equipment
Persons who have previously been prescribed PEP (post-exposure prophylaxis) with either:
Continued risk behavior
Multiple courses of PEP
Qualifications:
GFR>30
HIV antibody negative with testing in past week
No active HIV symptoms
Step 6:
Consider pre-exposure prophylaxis (PrEP) for HIV
For more information, the CDC has resources for PrEP prescriptions for Primary Care Providers

## Slide 39
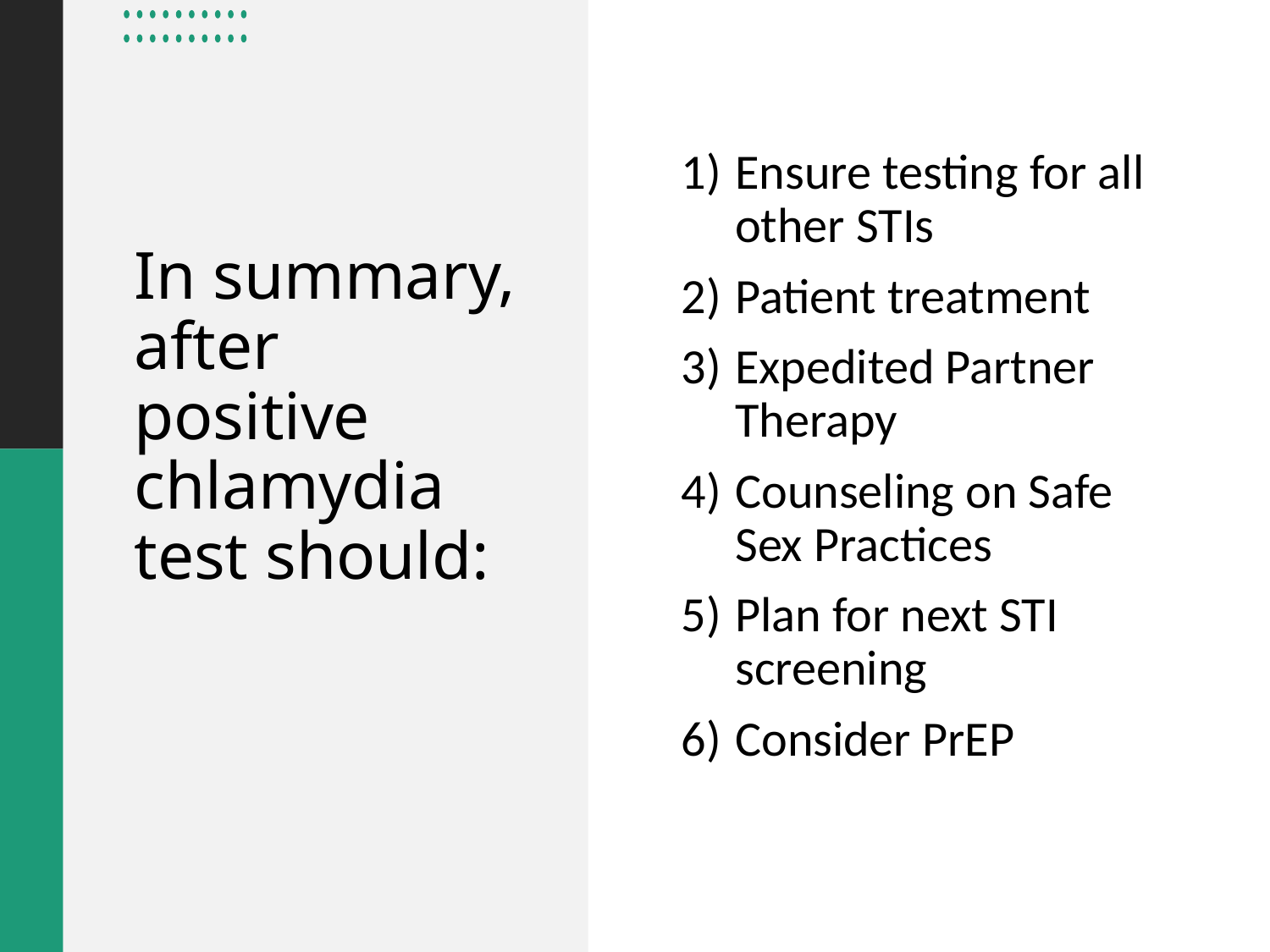

Ensure testing for all other STIs
Patient treatment
Expedited Partner Therapy
Counseling on Safe Sex Practices
Plan for next STI screening
Consider PrEP
# In summary, after positive chlamydia test should:

## Slide 40
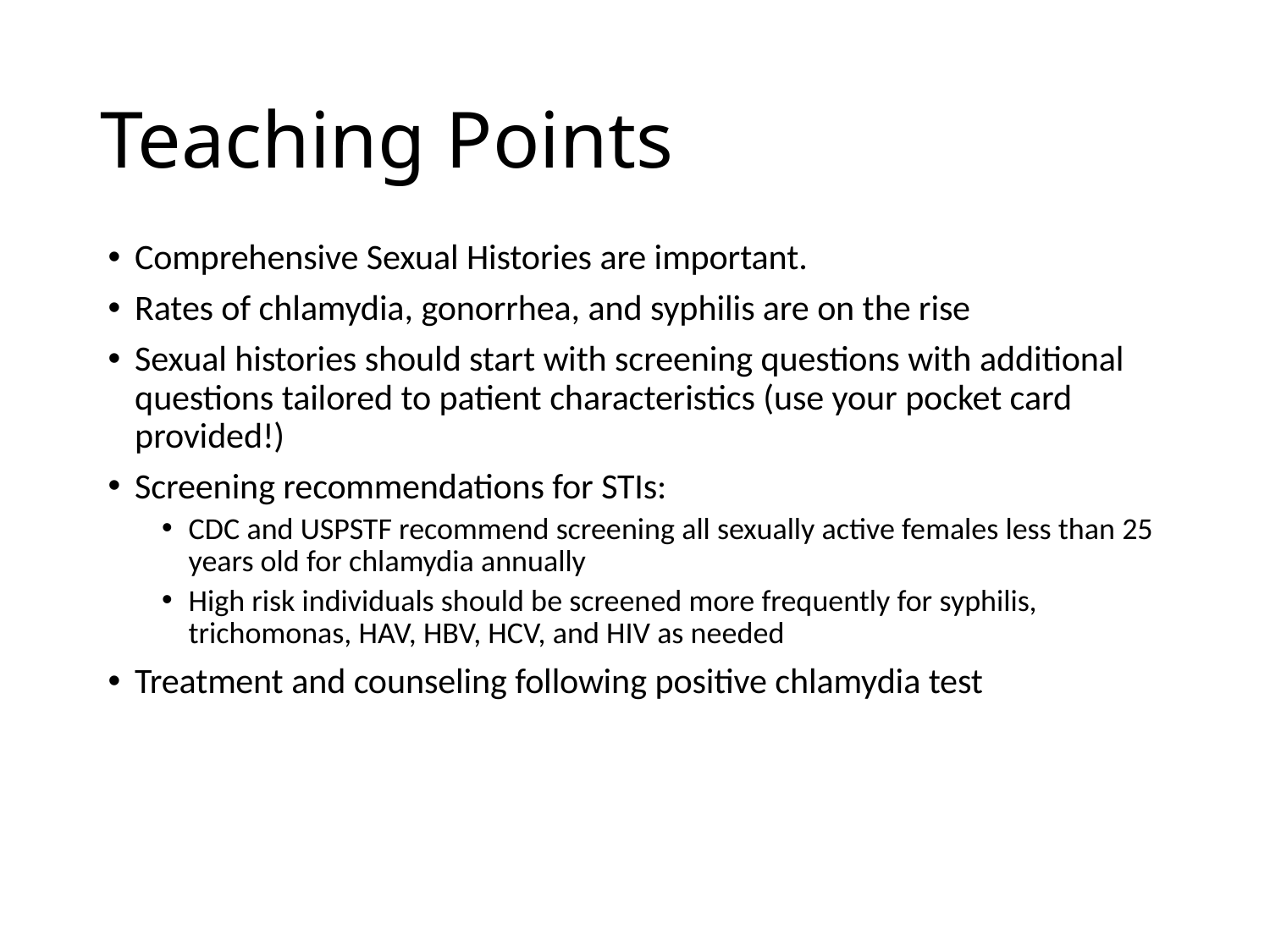

# Teaching Points
Comprehensive Sexual Histories are important.
Rates of chlamydia, gonorrhea, and syphilis are on the rise
Sexual histories should start with screening questions with additional questions tailored to patient characteristics (use your pocket card provided!)
Screening recommendations for STIs:
CDC and USPSTF recommend screening all sexually active females less than 25 years old for chlamydia annually
High risk individuals should be screened more frequently for syphilis, trichomonas, HAV, HBV, HCV, and HIV as needed
Treatment and counseling following positive chlamydia test

## Slide 41
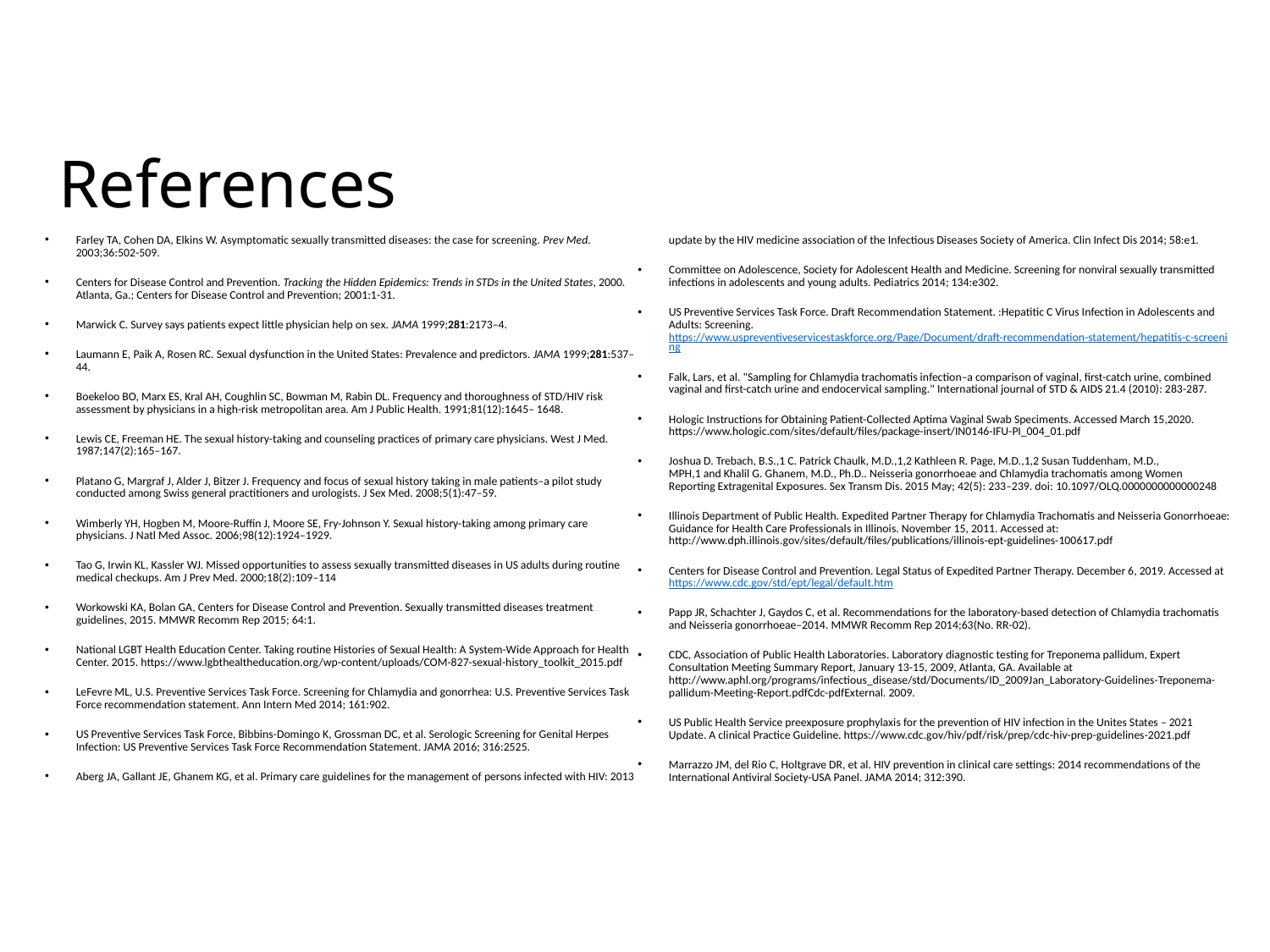

# References
Farley TA, Cohen DA, Elkins W. Asymptomatic sexually transmitted diseases: the case for screening. Prev Med. 2003;36:502-509.
Centers for Disease Control and Prevention. Tracking the Hidden Epidemics: Trends in STDs in the United States, 2000. Atlanta, Ga.; Centers for Disease Control and Prevention; 2001:1-31.
Marwick C. Survey says patients expect little physician help on sex. JAMA 1999;281:2173–4.
Laumann E, Paik A, Rosen RC. Sexual dysfunction in the United States: Prevalence and predictors. JAMA 1999;281:537–44.
Boekeloo BO, Marx ES, Kral AH, Coughlin SC, Bowman M, Rabin DL. Frequency and thoroughness of STD/HIV risk assessment by physicians in a high-risk metropolitan area. Am J Public Health. 1991;81(12):1645– 1648.
Lewis CE, Freeman HE. The sexual history-taking and counseling practices of primary care physicians. West J Med. 1987;147(2):165–167.
Platano G, Margraf J, Alder J, Bitzer J. Frequency and focus of sexual history taking in male patients–a pilot study conducted among Swiss general practitioners and urologists. J Sex Med. 2008;5(1):47–59.
Wimberly YH, Hogben M, Moore-Ruffin J, Moore SE, Fry-Johnson Y. Sexual history-taking among primary care physicians. J Natl Med Assoc. 2006;98(12):1924–1929.
Tao G, Irwin KL, Kassler WJ. Missed opportunities to assess sexually transmitted diseases in US adults during routine medical checkups. Am J Prev Med. 2000;18(2):109–114
Workowski KA, Bolan GA, Centers for Disease Control and Prevention. Sexually transmitted diseases treatment guidelines, 2015. MMWR Recomm Rep 2015; 64:1.
National LGBT Health Education Center. Taking routine Histories of Sexual Health: A System-Wide Approach for Health Center. 2015. https://www.lgbthealtheducation.org/wp-content/uploads/COM-827-sexual-history_toolkit_2015.pdf
LeFevre ML, U.S. Preventive Services Task Force. Screening for Chlamydia and gonorrhea: U.S. Preventive Services Task Force recommendation statement. Ann Intern Med 2014; 161:902.
US Preventive Services Task Force, Bibbins-Domingo K, Grossman DC, et al. Serologic Screening for Genital Herpes Infection: US Preventive Services Task Force Recommendation Statement. JAMA 2016; 316:2525.
Aberg JA, Gallant JE, Ghanem KG, et al. Primary care guidelines for the management of persons infected with HIV: 2013 update by the HIV medicine association of the Infectious Diseases Society of America. Clin Infect Dis 2014; 58:e1.
Committee on Adolescence, Society for Adolescent Health and Medicine. Screening for nonviral sexually transmitted infections in adolescents and young adults. Pediatrics 2014; 134:e302.
US Preventive Services Task Force. Draft Recommendation Statement. :Hepatitic C Virus Infection in Adolescents and Adults: Screening. https://www.uspreventiveservicestaskforce.org/Page/Document/draft-recommendation-statement/hepatitis-c-screening
Falk, Lars, et al. "Sampling for Chlamydia trachomatis infection–a comparison of vaginal, first-catch urine, combined vaginal and first-catch urine and endocervical sampling." International journal of STD & AIDS 21.4 (2010): 283-287.
Hologic Instructions for Obtaining Patient-Collected Aptima Vaginal Swab Speciments. Accessed March 15,2020. https://www.hologic.com/sites/default/files/package-insert/IN0146-IFU-PI_004_01.pdf
Joshua D. Trebach, B.S.,1 C. Patrick Chaulk, M.D.,1,2 Kathleen R. Page, M.D.,1,2 Susan Tuddenham, M.D., MPH,1 and Khalil G. Ghanem, M.D., Ph.D.. Neisseria gonorrhoeae and Chlamydia trachomatis among Women Reporting Extragenital Exposures. Sex Transm Dis. 2015 May; 42(5): 233–239. doi: 10.1097/OLQ.0000000000000248
Illinois Department of Public Health. Expedited Partner Therapy for Chlamydia Trachomatis and Neisseria Gonorrhoeae: Guidance for Health Care Professionals in Illinois. November 15, 2011. Accessed at: http://www.dph.illinois.gov/sites/default/files/publications/illinois-ept-guidelines-100617.pdf
Centers for Disease Control and Prevention. Legal Status of Expedited Partner Therapy. December 6, 2019. Accessed at https://www.cdc.gov/std/ept/legal/default.htm
Papp JR, Schachter J, Gaydos C, et al. Recommendations for the laboratory-based detection of Chlamydia trachomatis and Neisseria gonorrhoeae–2014. MMWR Recomm Rep 2014;63(No. RR-02).
CDC, Association of Public Health Laboratories. Laboratory diagnostic testing for Treponema pallidum, Expert Consultation Meeting Summary Report, January 13-15, 2009, Atlanta, GA. Available at http://www.aphl.org/programs/infectious_disease/std/Documents/ID_2009Jan_Laboratory-Guidelines-Treponema-pallidum-Meeting-Report.pdfCdc-pdfExternal. 2009.
US Public Health Service preexposure prophylaxis for the prevention of HIV infection in the Unites States – 2021 Update. A clinical Practice Guideline. https://www.cdc.gov/hiv/pdf/risk/prep/cdc-hiv-prep-guidelines-2021.pdf
Marrazzo JM, del Rio C, Holtgrave DR, et al. HIV prevention in clinical care settings: 2014 recommendations of the International Antiviral Society-USA Panel. JAMA 2014; 312:390.
